# Supplementary material for: It is beyond remuneration: Bottom-up health workers’ retention strategies at the primary health care system in Tanzania
Source: PLoS One. 2021 Apr 8;16(4):e0246262. doi: 10.1371/journal.pone.0246262 (PMC8031416; doi:10.1371/journal.pone.0246262)
Supplement: S1 Transcripts — (DOC) [file pone.0246262.s001.doc]

HM-1

IQ (watu wanaongea) Kama ambavyo tumekwisha zungumza ….. kama ambavyo tume kwisha zungumza, malengo ya utafiti huu ni kuweza kuibua mikakati na mipango inayo anzia kutoka kwa wadau wenyewe kwenda juu serikalini badala ya kutoka serikalini kwenda kwa wadau katika kutunza rasilimali watu katika sekta ya afya ….. kwa kuanza ningependa kujua una mda gani katika wadhifa ulio nao sasa

RR nina mwaka mmoja na miwezi miwili

IP miwezi miwili

IQ na una mda gani katika eneo hili la kazi? ukiachana na wadhifa tu (sauti ya mashine)

RR nina takribani miaka miwili (asante sana)

IQ na pengine unafahamu maeneo mengi kuna waajiri tofauti wa wafanyakazi wa afya; wapo sehemu ambapo wana ajiriwa moja kwa moja kutoka kwenye halmashauri, kuna waajiriwa wa sekta binafsi na waajiriwa kutoka taasisi mbali mbali wanakuwa wameingizwa katika wilaya kutoa huduma. Pengine katika wilaya yako hali ikoje (sauti ya piki piki) tunapo zungumzia haya makundi ya waajiri

RR kwa upande wa serikali, watumishi wengi wameajiriwa na mkurugenzi, tuseme wapo chini ya mkurugenzi. Wale ambao walikua chini ya wizara au serikali kuu, wengi wamesha staafu kwa mfano watu wa magonjwa ya akili ndo baadhi ya watu waliokua kwenye serikali kuu. Lakini pia ndani ya wilaya kuna baadhi ya hospitali ambazo sio za serikali, za binafsi maanake nao pia wana aajiri, kwa hio kuna baadhi ya waajiriwa ambao wata simamiwa na taasisi na wengne wa serikali kwa maana wapo chini ya mkurugenzi mtendaji .

IQ (mhhh) pengine kuna sehemu tumeaona kuna waajiriwa wa mkapa foundation ambao wanafanya kazi kwa mkataba na mkapa foundation lakini wanakua wako responsible kwa halmashauri na mkataba wao ukiisha wana swap kuingia kwenye halmashauri , kuna wafanyakazi wa namna hio hapa kwenu?

RR haa kwetu hatuna, bado mkapa hajaingia halmashauri ya rombo

IP hakuna taasisi nyingine yoyote ambayo ina wafanya kazi huku kama JHPIEGO, GENDER HEALTH, eehhh MDH ambao nao tunajua wanafanya kazi kama mkapa foundation kwa kiasi kikubwa

RR baadhi ya mashirika kama hao GENDER HEALTH yapo lakini hawana wafanyakazi per se, wenyewe wanatumia ambao tupo nao kwenda kufanya kazi zao . yeah

IQ Nakushukuru sana. Na inapo kuja swala la kuajiri watumishi wapya wa afya hapa katika halmashauri yako, hali ikoje? Kuna kua na uchambuzi akinifu wa mahitaji? Na huo uchambuzi akinifu unawahusisha (watu wanaonga nyuma) watu gani na ni kwa kiasi gani ofisi yako kama ofisi yako inahusika kwenye huu mchakato mzima?

RR katika swala zima la uajiri wa watumishi mara nyingi tunafuata IKAMA na IKAMA ambayo sasahivi ipo up to date ni ya 204 mpaka 2019. Kwa hio sasa ile IKAMA ina kuonyesha kwa mfano, tuna hitaji madaktari wenyedegree kiasi gani kwa ngazi ya wilaya, kwa hio sasa wewe utakua unamba kulingana na mahitaji yako, lakini unavyo omba, sio lazima upate ulicho omba mara nyingi wankupa ambao wapo available kwenye soko, kwa hio serikali itakupangia wale ambao wapo sokoni. Lakini mara nyingi tunaomba , kuna cadre ambazo tunaomba lakini bado hatuna . kwa mfano kuna watu wa, kwa mfano watu wa nini labda, kwa mfano watu wa maabara ni wacahce sana, mara yingi mnakua mnaomba hao watu wa phamarcy au wasaidizi wa cadre za chini za pharmacy, mtakuta mnawaomba lakini, mnawainisha kila mwaka kwenye bajeti zenu lakini basi ndo tunapewa kulingana na availability iliopo

IQ Kwa mfano kwa IKAMA kwa sasa yam waka huu inakuonyesha kwamba unahitaji wafanyakazi wangai kwa ujumla wake na wewe unao wangapi?

RR yaani ni ngumu kukumbuka ile jumla, of course najua inahitajika takribani watumishii kama ….. karibia 600 na kitu laikini ambao wapo ni kama almost half ya hio mia tatu na arubaini na kitu, kwa hio utakuta tuko nusu

IP na Imani mwisho wa mazungumzo yetu naweza pata copy ya takwimu hizo

RR jio nadhani, kwa leo, katibu ndio anakuaga nazo, kwa kesho nadhani ndo uta ipata (kwa maelekezo yako najua nitapata) ndio ndio

IQ na ni eneo gani ambalo lina uhaba Zaidi ukiachana na maabara, kua eneo gani jingine ambalo unaona lime athirika Zaidi

RR kuna watu wa maabra, kama tuliivyo sema, kuna watu wa pharmacy, kwa sasa tuna wafamasia tu wawili, tulikua tuna hitaji zile middle cadres wale ambao wana diploma au certificate za maabara, za pharmacy wawepo kituoni. Kwa mfano unakuta kabisa hawapo. Sasa inakua ni ngumu ku operate

IQ Unafahamu kua kuanzia mwaka jana kumeanza utaratibu wa kupost wafanyakazi moja kwa moja kwenda kwenye vituo kutokea wizarani. Na hii iliokana na tafiti zilizo tangulia ambazo zilionyesha kwamba utaratibu wa kutangaza nafasi za ajira unakua na milolongo mingi na wakati mwingine kuna kua na urasimu unaofanya watu wengi wana potea kabla hata hawaja omba kazi kwa mara ya kwanza. Je pengine utaratibu huu mpya …. Utaratibu wa zamani ulikua na hizo changamoto, sasa tangu utaratibu huu mpya wa kuwatuma watu moja kwa moja kwenye vituo, nyinyi kama halmashauri, ni changamoto zipi ambazo mmekutana nazo? Mmeshapokea wafanyakazi wowote ambao wametumwa moja kwa moja katika mfumo huu mpya ulio anza mwaka jana ?

RR yeah (watu wanaongea nyuma) tumepokea takri bani ukianzia mwaka jana wanakaribia themanini, kwa sababu najua mwaka huu kuanzia julai tumepokea karbia hamsini na kitu kwa hio karibia na sitini, wamesharipoti lakini shida ni kwamba changamoto ni wale wale, ni nurses na baadhi ya Cos lakini kuna cadre zile ambazo tunahitaji lakni ujio wake ni mdogo sana. Alafu kuna wengie kwa mfano hawa medical attendants, wanaletwa yaaani mpaka sasa hivi wamesha zidi ile IKAMA yaani wamesha flood

IQ Na pengine mnapo letewa sasa medical attendants ambao wamezidi, mnachukua hatu gani? Mnafanya utaraibu gani? Mna wa accommodate tu wote au mnasema jamani ebu mtupunguzie idadi hii, tuongezeeni hii, au hakuna tena control ya nyinyi kusema idadi mnayo taka kwasababu sasa wana tuma moja kwa moja?

RR of course sisi tulisema tuwapokee kwanza alafu tufanye utaratibu, tupitie kwa afissa utumishi, tuandike barua kwa katibu mkuu wizara ya afya. Tumwambie kwamba sasa hawa tulio nao sasa wameshatosha tunaomba kama inawezekana mtubadilishie, watufanyie replacement kwa hawa ambao wamewatuma. Yeah, kwa hio tuliona busara tu kuliko kuwarudisha moja kwa moja tu bila barua, sis kama halmashauri tuandike barua kwa katibu mkuu ili tuwez kuipeleka, kama wata tu consider, its okay. Lakini tukiona vipi tunaeza tuka kaa nao tu, naamini kuna wanao phase out kwa hio wanaweza waka tusaidia sisi ku dwell soon

IQ Na pengine wanapofika hapa sasa hawa waajiriwa wapya ambao wanakuwa wamekua posted moja kwa moja, wengine wanatoka mwanza, wengine wanatoka kigoma, wanapo fika hapa mna wa accommodate vipi wakati bado hawajaweza kutulia katika makazi ambayo ni rasmi kwa ajili ya kutumika?

RR mara nyingi wakisha repor hua tunawapa wiki mbili za, kama kwenda kujipanga wakati huo na sisi tuna jaribu kuwa andalia labda ile subsistence allowance, ile ya kujikimu. Mara nyingi ni utaratibu wa wizara ikiwa force tunategemea na yenyewe itupe ….. (watu wanaongea) tunategemea wizara ikisha, iki waforce tu huku iwatume kama ilivyo walimu, walimu wana tumwa kituoni kwa hivyo basi kwenye halmashauri huwa tunatumiwa hela za kujikimu, lakini sasa hivi wanacho kifanya wana waleta lakini hamna hela ya kujikimu. Kwa hio inabibi mpambane tu wewe na mkurugenzi wako muangalie namna ya, lakini kwa kiasi kikubwa tunawasaidia wapate hela za kujikimu mapema

IP kwa hio hela za ujikimu zina toka kweye halmashauri?

RR yeah, inabidi tu itoke kwenye halmashauri kwa sababu kuna (hili ni fungu la halmashauri) eehh, (sio fungu la kutoka …) hapana (mhhh)

IQ Na wanapo fika vipi, mnawaonyesha mzingira ya kazi tu au na mazingira ya jamii kwa ujumla na tamaduni za hapa?

RR kwa kweli mara nyingi hua hatufanyi hivyo, kuwaonyesha ehhh, mara nyingi tunawaonyesha kituo ambacho wanatakiwa kwenda, unampa, anapelekwa pale, anakabidhiwa kwa mkuu wa kituo pale ehhh, hizo nyingi hatufanyi kwa kweli kulingana na….. nyingi hatufanyi.

IQ unatazamaje umuhimu wa kuwaonyesha utamaduni wa mahali husika kama miongoni wa misingi ya kuwafanya waji feel ni sehemu ya jamii n ahata wakae mda mrefu badala ya kufikiri kwenda kwenda maeneo mengine.

RR yeah, nadhani hiki ni kitu kizuri,zile orientation kwa kweli ni kitu cha msingi sema shida tunayo pata wanakuja mmoja mmoja , ingekua wanakuja as a package unaweza kuwafanyia, ila sasa akija moja mmoja uta morient leo kesho mwingine kwa hio inakua…. Ile ndo inatupa challenge (watu wanaongea, sauti ya gari) lakini ni kitu kizuri ambacho kinaweza kika encourage wakabaki nasi kwa mda mrefu

IQ na je tatizo la watumishi kuhama hama kutoka katika wilaya yako kwenda maeneo mengine, hali ikoje?

RR hilo lipo, lipo. Wakati mwigine tunajaribu kulizuia ili yule mtumishi, kwasababu kuna wengine wakikaa mikoa miwili mitatu wanataka wahame, kwa hio kama hakuna sababu za kimsingi basi unamwambia bwana inabidi ubaki hapa. Wengine hawataki kutumia moshi hapa mjini, kwa hio inabidi uwe firm otherwise utawaapoteza wote

IP mhh, na watu wanao omba kuhama ni wengi?

RR ni kweli wanakuja, wanakuja kweli wanakuja wengi

IP ni kundi lipi Zaidi linalo omba kuhama

RR ni hiki kizazi kipya, hawa walio ajiriwa juzi juzi hapa ndo wana hio kasumba ya kuhama, mtu anakuwa kama amekuja tu ku secure kazi, alafu, tena mbaya Zaidi mtu haja maliza hata miezi sita anataka ahame. Kwa hio inakua ile, unaona kabisa mtu alikua anatafuta confirmation apate kazi ili akafanye mambo mengine, kwa hio ndo kitu kama hicho.

IP na ni cadre ipi hasa?

RR mimi kwa kweli ambao nakumbana nao hapa ofisini ni nurses, nurses ndo wana sumbua

IQ na wanao omba kuhama ni wale ambao wanakua tayari wamesha olewa au bado hawaja olewa?

RR wengine hawajaolea, mtu anataka labda ahamie mjini, yaani ile ni kasumba kwamba mjini kupo vizuri na nini

IQ na pengine ni kwa kiasi gani mna jitahidi kuwapa (sauti ya engine) motisha watumishi wenu ili wasiweze kufikiria kuhama, pengine wanahama kwa sababu motisha zipo ndogo

(sauti ya engine na watu wanaongea nyuma)

RR kwa kweli tunajitahidi, kwa mfano wakija, kama kituo alicho enda kina nyumba wengi tunajitahdi apate nyumba, lakini kuna sehemu nyingi nyumba ni changamoto tunajitahidi angalao kama ni hela ya kujikimu wapate mapema basin a mshahara, siku hizi hawachelewi, mwezi wa kwanza wa pili wameshaingia kwenye pay role. Kwa hio mara nyingi wengi wakihamaa ni zile endeavors tu kwamba mtu anaona mjini ni kuzuri, ni ile kasumba tu.

IQ vipi mna mfumo wa kuhakisha kila baada ya mda mtumishi anaweza kuendelezwa kitaalama? Au inakua ni at your own initiative wewe ujitafutie baada ya muda ujue utajipilekaje shule?

RR hiyo, hiyo sasa tunaiweka kwenye mpango wa mafunzo lakini sasa inakuwa… kwa sababu ya fillings zinakua ndogo nayo inakuwa haiwezi ku accommodate watu wengi. Kwa hio wakati mwingine huwezi kumzuia mtu kwenda shule kwa sababu ya nini, haujaweka kwenye… of course tunatakiwa tufuate mpango, mfano kama mwaka huu tumesema wanaenda watu kumi kusoma, tuzingatie waende watu kumi. Lakini mwingine anasema mimi nitajigharamikia naomba niende lakini una angalia pia kama ametimiza mda wa kwenda kusoma. Kuna wengine wanakuja hivi, kwa mfano umeambiwa waajiriwa wapya ana miezi mitatu anataka aende shule, sasa yaani unapata challenge pia kwa sababu ukiwaruhusu wote tena nao wanapata hio surge ya nini… crisis ya watumishi tena.

IQ Na katika mpango wenu wa kusoma ni ….. ambao unasema hautoshi. Kwa wastani hua mna accommodate watu wa ngapi kwa mwaka ?na ni cadre zipi hasa mnazip kipaumbele

RR kwa wastani (watu wanaongea ) nadhani ni kama watuishi kumi, roughly watumishi kumi kwa mwaka ambao yaani ni kama ku wasupport tu, huwezi kusema kwamba una cater ile full sponsorship yani mara nyingi ni kama ku wa support. Na mara nyingi wanao enda sana ukiangalia wote ni clinical officers na nurses (okay) mara nyingi

IQ na kwa ngazi ya degree, ambayo nip engine wanahitaji kwenda kufanya shahada ya pili?

RR shahada ya pili kwa sababu ya yay a gharama kweli inakuwa ngumu, inakua ngumu. Hata ukiweka itakua impractical. Kwa mfano hata sasa hivi ukisema ukawasomeshe MD kwa mfano hivi vyuo vya private kwa kweli inatupa shida sana. Kwa sababu mtumishi ameenda labda Hubert Kairuki ni millioni sita, sasa akikuambia umlipie yote milioni 6 utakuta ni almost budget nzima ambayo mmetenga kwa watumishi wote kwa hio inakua ngumu kwa kweli, watumishi wata lalamika hapa na pale lakini unakuta ndo uhalisia

IQ kuna maeneo mengine ambayo wao hawashindi kupeleka watu kusoma kwa sababu ya fedha, wao wanashindwa kupleka watu kusoma kwa sababu ya kuogopa kwamba watakapo waongezea sifa wataondoka hawata bakia hapo. Kwenu hapa pengine hofu hio bado ni tatizo? Mnaogopa mtu alikua ni clinical officer akiondoka akienda kufanya MD akirudi hapa anawaambia mimi siwezi tena kukaa huko vijijini mimi naondoka kwenda mjini

RR hio kwa kweli sio hufu, hofu kwa kweli ni fedha, kwa sababu kuna halmashauri nyingi mapata ya ndani ni makubwa kwa hio inapo hitaji kwenda kuwasomesha inakua sio tatizo, lakini kwetu challenge kubwa ni capital. Na pia ukiwapeleka wengi kwa wakati mmoja nayo ni ishu kwa hio inabidi ubalance hio.

IP mhhh, pengine hapa kwako una MDs wangapi? Katika wilaya yako

RR MDs ambao wapo serikalini sasa hivi tupo, ni kama watano, watano so far.

IP na mahitaji yako kwa wilaya ni MDs wangapi?

RR inatakiwa minimum tuwe eight, yani tuwe angalao kulingana na IKAMA (sauti ya engine) sina uhakika kama nakosea lakini minimum ni ya nane

IQ Okay, okay ….. ahsante sana. Na ….. umesema wanaopenda kuhama kutoka hapa kwako ni wale wa kizazi kipya na wengi na wengi wanataka kwenda mijini(mhhh) okay. Na pengine katika kukubaliana na hiyo hali mmeweka mikakati gani sasa ili kuhakikisha kwamba hawahami?

RR kwanza, mkakati namba moja ni kwanza kitu cha kwanza ni kuwa elimisha tu kwamba yani hakuna tofauti kubwa sana kiutendaji, kama unahamia mjini of course ni ile kasumba lakini sidhani kama kuna additional advantage sana mjini. Alafu kingie ni kuwakumbusha pia, huwezi , umeajiriwa leo baada ya miezi sita unataka uhame hata hujawa confirmed kazini unataka kuhama. Kwa hio wengi tuna washauri, kaa hata angalao uwe confirmed kazini angalao au angalo ufanye kazi miaka miwili mitatu ndo inawea ikawa sound hata kwa mtu kukuruhusu kuhama wilaya

IQ Kwa haraka haraka, pengine unakumbuka idadi ya wafanyakazi walio hama siku za karibuni?

RR ni kama kuanzia mwaka, tuseme kuanzia julai mwaka uliopita mpaka sasa hivi nadhani kama watumishi watano hivi wamesha hama.

IP watumishi wa tano?

RR mhhh, watano. Lakini of course wanakuwa na sound reasons, unakuta huwezi kumzuia. Labda mwingine ameolwa kule na amesha kaa hapa sana na wametengana na familia yake basi , (watu wanaongea) saa nyingine inakuwa justifiable

IQ mbali na hawa watano waliohama kwa kufuata utaratibu, kuna wafanyakazi wowoe wame amua tu kuacha kazi na kuondoka?

RR hapana, sina, sina, hakuna mtumishi alie fanya… Ila kuna mmoja amesha andika barua kabisa, yeye alienda kusoma kabisa, ni MD alienda kusoma, ni CO alienda kusoma MD. Lakini baada ya kusoma kumbe kipindi hicho alikosa ufadhili, ile halmashauri haiku cater kumsomesha kwasababu ada iikua ni kubwa . so baadaya kusoma kule, kumbe wakati anasoma kule aliomba chuo flani nadhani kama sio Mount Meru sijui, kipo Arusha lakini kimsomeshe, with intention kwamba nadhani waliweka makubaliano kwamba baada ya kumaliza angenda kua mwalimu sijui wa kitu gani huko kwao (okay kuwa tuitor) eeeh, basi alivyo maliza tu chuo akawa ameandika barua kwamba yeye anaomba yani kuacha kazi kwa hiari, sasa ile, kwa sababu ampata kazi sehemu nyingine ambayo anadhani ina.. japo tulimshauri kufanya kazi lakini akasema yeye yupo committed na amesha weka makubaliano hayo kwa hio nilipatisha barua hiyo ikaenda kwa mkurugenzi, nadhani aliweza kum….

IQ eheee, kama tulivyo sema miongoni wa vitu vya msingi ni jamii kushiriki katika mipango hii ya kuhakikisha mipango hii ya kulinda serikali watu inakua ni shemu yake. Ni kwa kiasi gani sasa jamii inahusika hapa katika kuweka mikakati ya kuhakikisha kwamba mazingira ya watumishi wa afya yanaweza kua ni bora na salama kiasi cha kwamba wanavutiwa kuendelea kufanya kazi hapo? Kwa mfano, jamii inaelewa umuhimu wa kua na wafanyakazi wa kutosha katika eneo hili?

RR kwangu mimi, kwa rombo wengi wana uelewa na wanajua kabisa, kwa kweli sijapata tatizo la watumishi kwenda kituoni kuleta malumbano na wana nchi, labda kama yeye ata misbehave lakini otherwise ule mtazamo wa watumishi kwa wananchi ni mzuri, upo very positive.

IQ mhhh, ni, najua katika mfumo wa serikali za mitaa kuna kamati za afya kule chini. Ni kwa kiasi gani hizo zipo hai hapa katika wilaya yako?

RR hizo kamati za vituo zipo hai, karibia kila zahanati au kituo cha afya kina kamati nan do zinazo plan zile day to day activities za kituoni kwao. Kwa mfano kama wanaamua, mara nyingi kuna hela ya CHF za ile bima ya jamii, kwa hio maamuzi ya zile fedha, ile kamati inaamua. Na huwa wanakua na vikao kila quarter, kwa hio kwa kweli hizo ziko hai kwa mda mwingi.

IQ Na kama unavyo sema hizo ni zile za vituoni, lakini nafahamu kuna zile kmati za kata na za afya za vijiji kama vijiji, ukiacha zinazosimamia vituo (mhhh) ambazo hizi za Kata na vijiji moja ka moja zinakua na share kubwa ya wananchi, (ndio ndio). Ni kwa kiasi gani hizo ziko hai katika wilaya yako?

RR hizo zipo hai kwa maana ya kwamba, maanake wanavyo itisha hivi vikao vyao, mara nyingi mganga mkuu wa kituo husika anakua pia ni representative kule kwa hiyo lazima anakua pale kama kuna issue za afya zimejitokeza basi anaweza kuclarify na vitu kama hivyo.

IQ Naamini pia katika ngazi za wilaya zipo pia kamati afya za wilaya, (mhhh) na wakati mwingine zinahusisha hata wana siasa na wadau wengine wa maendeleo. Ni kwa kiasi gani kamati hio ipo hai hapa kwako na ushiriki wako ukoje kwenye hio kamati?

RR hio kamati ipo hai, na tunashiriki, yeah. Kabisa nashiriki kule (interview kacheka, unashiriki kule) eeh kule kama kuna challenges, na wale kwa sababu nao wapo, wengi ni madiwani wapo kwenye kata zao respective, kama kuna matatizo yamejitokeza labda hospiatli basi wanakuja kuyaibua pale

IQ okay, vipi muingiliano wa wanasiasa na watumishi? Kuna tatizo kama hilo hapa kua wanasiasa wanawaingilia wakati mwingine mipaka ya taaluma, hata kufanya wakati mwingine kazi kua ngumu?

RR yeah, hilo halipo sana, lakini occasionally linaweza kujitokeza. Na pale basi linapo jitokeza, sisi kama ….. kama wataalumu tuna simamia taaluma zetu. Tuaweka siasa pembeni, pale ambapo inastahili taaluma iwepo basi tunaisimamia.

IQ je kuna kipindi ambacho wanasiasa hao wanakualika katika mikutano ya jamii? Pengine unazungumzia matatizo kwa ujumla ya afya katika wilaya yako kwenye mkutano wa hadhara wa jamii nap engine kutoa ushauri wa kitaalamu?

RR yeah kuna, ofcourse kuna baadhi ya scenario ikitokea kwa mfano wakasema kwamba tunamuhitaji mganga mkuu aje aclarify issue flani , basi hua naenda. Yeah

IP okay, hua unaitwa kwa dharura au unakua umepewa taarifa kabla kwamba tuna andaa mkutano kama huu na tutatamani uwepo

RR hua wanakuambia before, sio dharura (okay) wanakuambia before, sio dharura

IQ okay, na unafikiri ni nini kifanyike ili kuhakikisha kwamba wafanyakazi hawa hami hami kutoka kwenye wilaya yako kwenda wilaya nyingine?

RR mi nadhani ni kuendelea tu kuwapa elimu, kwamba kunai le, ile mentality kwamba mjini ndo kuna maisha bora. Hio kitu kimetawala. Kwa hio mtu ana mtu anakuja hapa ile kusecure tu ajira alafu sasa baada ya kusecure ile ajira anataka sasa ahamia ile sehemu ambayo alikua ana wish kuishi maisha yake. Kwasababu unajua wizara ina post mtu ilimradi, sio lazima sehemu uliochagua. Sasa mtu akisha pangiwa, anajua anaenda rombo, amepangiwa sehemu ambayo labda siku jipanga katika maisha yangu. Kwa hio mimi naend kureport nikisha pata tu ile cheque namba basi nataka nirudi sehemu niliokuwa naihitaji. Sasa hio ndo tunahitaji kuiondoa

IQ mhhh, nap engine unazitazama changaoto gani mbeleni katika siku zijazo katika kuhakisha kua unakua na watumishi wa kutosha wilayani kwako.

RR yeah, of course changamoto ninazo ziona mbeleni ni maswala ya hizi nini, hizi incentives kwa watumishi wapya, especially kwa mfano nyumba, nyumba kwa kweli imekua changamoto kubwa. Kwa sababu ile pace ya kujenga nyumba na watumishi wanavyo kuja kwa kweli zinatofautina. Kwa hio… (watu wanaongea)

IQ na pengine nyumba mnazitoa kwa watu wa cadre gani hapa?

RR nyumba, preferably tuna angalia, kwa fano labda kituo kina, mara nyingi nusres na madaktari ambao tunahitaji all weather, kwa sababu wale watahitajika mda wote pale hospitalini basi ikitokea nyumba, wale ndo tunawapa first priority.

IQ na kwa maeneo ambayo hakuna nyumba, tuna;nafahamu kuwa standing order inazungumza stahihii kwa designation kwa mfano ya madaktari, kama hakuna nyumba, unatakiwa kuma posho ya nyumba. Je hilo kwa hapa wilayani kwako, hali ikoje?

RR eeeh, hapa wilayan kwangu hapa ukweli mimi najua ukweli tukisema tutoe hela ita iwia halmashauri mzigo, mimi napambana na kuhakikisha wanapata nyumba. Kwa hio kwa kweli madaktari ambao ni ngazi ya degree ambao wapo wilayani hapa wote wana nyumba.

IP wote wanan nyumba?

RR mhhh, nashukuru Mungu purukusheni zinasababisha waelewe ukisha waambia na wanajua kabisa.. na mimi nakumbuka hata tulipo repoti sisi kwa sababu nyumba zilikua bado haujawa tayari, ilibidi tuwekwe hotelini kwa mda ili tungojee ziwe kamili alafu tuka hamishiwa kule. Kwa hio it was very encouraging; wal wana nyumba kwahio …. (mhhhh) kwa hio hilo halina, hatujampata ambae amekosa na inabidi tumlipe, haija fikia huko.

IP lakini mnalitazamaje, kwa mfano mna madaktari watano umesema (ndio ndio), na una uhaba wa minimum of three kama uivyo sema (ndio ndio) kwa kutumia IKAMA (ndio ndio) lakini nina hakika tukichambua hio staffing level na population inavyo kwenda (mhhh) in the next five years utakuta sio nane tena, utajikuta unahitaji kumi na tano kumi na sita (ehee), mnajipangaje kwa kiasi kuhakikisha kua kutakua na nyumba za hao madaktari wote watakao kwepo nap engine kama hakuta kua na nyumba, unautazamaje uwezo wa halmashauri na utayari wa halmashauri kuweza kuwa accommodate hao wafanyakazi kwa kuwalipa posho zao za nyumba.

RR mimi nadhani yote yana wezekana, hilo la nyumba kwa sababu naona bado halmashauri bado inajenga nyumba kwa hio sina wasiwasi na prospctives MDs ambao watapangwa wilayani kwangu naamini wote wata pata nyumba na kama itashindikana kabisa kua hawezi kupata nyumba basi itabidi zile posho zao naamini haita kua kazi kubwa kwa halmshauri kuwa tazama

Okay, nakushukuru sana, that is very encouraging. Na pengine kuna motisha nyingine zozote ambazo mnawapa watumishi wenu mbali na au kuhakikisha tu kwamba wanapata nyumba?

RR zingine ni zile za msingi, kwa mfano kama ni on call allowance kama allowance kwa nurses wale, actually zile ambazo ni stahili zao za msingi wanapata

IQ Mhhh, na hizo zipo, zimeandikwa kwenye mpango wowote au ?

RR kwa mfano hii ya nursing allowance ya, ya kupata on call ipo kwenye mpango kabisa (ipo kwenye mpango) kabisa yeah, lazima uwawekee kwenye mpango (miongoni wa vitu ambavyo nita…) ehee ipo available hata mkihitaji kuiona mtapewa

IQ okay, nashukuru, nap engine kuna tofaui zozote za kichangamoto kati ya waajiri waliokuwa wameajiriwa mwanzoni na halmashauri na wale wano tumwa sasa hivi moja kwa moja na wizara?

RR kimsingi sioni kama kuna tafauti sana (ndio), au kwa sababu sijawahi ku experience mimi personally lakini, kwasababu wote wapo under, under day, whether umeajiriwa na mkurugenzi, amekuajiri wewe akapeeka majina juu au umekua posted mwishowa siku your immediately resposnsible boss, kwa hio hai, it wont makeany difference (okay). Japo ambao labda ambao wana ajiriwa kwa mkurugenzi ambao wanakua chini kabisa ya serikali kuu lazima wajiwajibishe kwa mkurugenzi, sasa wale ndo wanakuaga na changamoto mara nyingi wao wanaonaga kwa sababu yeye anaona hawajibiki kwa mtu yoyote hapa chini (ndio) anadhani anawajibika kwa ngazi ya juu (ndio), hio ndo ina impression tofauti lakini kwa wale ambao wamepangwa wawajibishwe na mkurugenzi

IQ kwa hapa una wangapi ambao wana report serikali kuu?

RR nakumbuka nilikua na mmoja, ambae amestaafu ambae alikua mtaalamu wa magonjwa ya akili (ndio)

IQ na kutumia uzoefu, unaona kwamba ki changamoto, alikua ni tofauti na wengine waliokwepo? Pengine yeye alikua anaona yupo kwenye level nyingine na wafanyakazi wengine hata katika namna alivyo kua anafanya kazi zake hapa?

RR hapana, he was very committed, hata hio kwake haikua ishu ya (okay) kuambatana nayo sana (okay). Aliku au vizuri, sema sasa inategemea na mtu na integrity ya mtu pia, kuna mwingine anaweza akajua kua yupo serikali kuu akaona yupo very superior kua yeye hawajibiki kwa DMO, kwa nani (mhhh)

IQ nakushukuru sana, na unatazamaje sasa mikakati na mbinu unazo tumia au katika kuzui au kujitahidi kuwa wafanyakazi hawahami. Unaitazamaje hii mikakati, unaiona kama mikakati ambayo ni endelevu, kwamba haita fika mahali ikafeli.

RR yeah, mimi naona kama ni mikakati endelevu kama, kwasababu ishu ya kujenga nyumba vitu kama hivo, kama ile pace sisi hatuzidi, kama watumishi wanakuja wengi kuliko ile kasi yetu sisi ya ujenzi, basi wale watu ukiwaeleza ….. wengi ni waelewa, sidhani kama wata despair.

IQ nayatazamaje mazingira ya kazi kwa ujumla, kwa mfano kwa watu ambao wamepata training kwenye vyuo vikubwa ambapo mazingira ya mafunzo ni tofauti sana (mhhhh) na mazingira ya wilayani. Unatazamaje haya mazingira ya kazi ?

RR ni tofauti of course. Mwanzoni hayana tofauti sana lakini ukija ukikaa, uzuri huku tuna good working teams, ile team work iko vizuri, kwa hio wengi wakiingiaa tu wanapata support kwa hio unajikuata una assimilate yani unajikuta sasa unaona ahhaaa mbona panatosha tu (mhhh) ehee yeah, sema sasa kwa watu ambao tupo over ambitious ndo tunaweza kuona sasa kama halmashauri hakuna fedha kwa sababu hakuna ile ya kufanya moonlighting kusema nitatoka hapa niende, ukimaliza masaa ya kazi niingi kituo flani nifanye fanye kazi nipate hela ya kunanilii sasa hio huku kwa sababu ni kijijini hakuna sera kwa sababu sehemu zenyewe ni chache pia, kwa hio hio ndo changamoto kubwa ambayo labda kwa MDs ndo inaweza ikawa ni hofu

IQ unashauri nini kifanyike sasa, katika ku balance na kupunguza hilo pengo kati ya walioko mjini na walioko vijijini ?

RR nadhani tukiendelea tu ku… kama mtu anapenda ku invest sio lazima ku invest sio lazima uinvest katika, sio lazima ufanye kazi ya udaktari nje ya kazi, kama unaweza kufanya biashara nyingine tofauti na udaktari mimi nadhani unaweza tu ku survive in villages

IQ kuna watu wanasema, unapo kua daktari , unapo enda kufanya kazi ya kuuza kuku una behave unprofessionally (kacheka) (sauti ya pikipiki)

RR hio nadhani sio proper (mhhh)

IP kwa hio wewe unashauri madaktari wawe wafugaji na wakulima

RR kabisa, inawezekana kabisa na wata make a goodlife in that.

IQ lakini nimesikia, Rombo ukiwa mgeni huuziwi ardhi, unaizungumziaje hilo?

RR hio inabadilika sasa hivi, sasa hivi tuna uziwa tuna, watu sasa hivi wengi wamejenga ambao hata wengi sio wa rombo (ahaa) ni hapa tu mkuu mjini ndo ilikua hio kasumba ipo, lakini sasa hivi ulishakua mji mdogo, imesha anza kubadilika

IQ nakushukuru sana, unashauri nini kifanyike kwa ujumla katika wilaya yako na katika vituo uilvyo navyo, ili kuhakikisha kwamba rasilimali watu hii ambayo ni adimu haiondoki badala yake inavutia watu kuja

RR mimi nadhani tu tuendelee kupeana tu ushirikiano ngazi za juu na za chini, kwa sababu inawezekana, mtumishi hapa, yupo hapa rombo lakini kumbe ana ndugu yake yupo wizarani. Basi yule ukimzuia kuhama, unakuta tu uhamisho unatoka from above. Lakini sasa tukiifuata tu ie miiko ya kazi, mimi naamini hakuna mtu anataka kufanya kazi kijini. Sisi wote tunatamani mjini lakini inafika mahali unasema ngoja na mimi niwasaidie saidie tu wana nchi (mhhh) mhhh.

I1Mimi rasmi nakushukuru sana, na pengine kama mtafiti msaidizi

I2 Hapana (watu wanaongea) mimi nakushukuru walao kwa mda wote tulio zungumzana DMO ametupa ushirikiano mkubwa, nashukuru

I2 basi mimi nikuombe wewe pia kama una swali ambalo ungependa kuuliza, nitakua tayari kujitahidi kulijibu

RR mimi sina swali per se, lakini nashukuru baadhi ya changamoto nyingi umesha zi capture kwenye questionarraire yako, kwa hiyo nadhani mtatusaidia angalao basi sisi ambao tupo kijijini maana watumishi wengi hawataki kuja kijijini. Utashangaa pale mtu amepangiwa rombo, kuna watu wanapangwa, saa nyingine hata watu wanapangwa, wizara inawapanga sehemu mbili tofauti, labda kisarawe na rombo, kwahio you don’t expect huyo mtu aje rombo. Utamshangaa amekimbilia kisarawe, kwa hio hizi wilaya ambazo zipo pembezoni kwa kweli tupewe angalao nanilii, tuseme upper hand kwa kweli wakati mwingine. Maana kule mtu ana impression rombo ni wapi huko, yani hata hajafika lakini ile impression kichwani, anaona kama ni sehemu ambayo hapakaliki. Lakini akija maisha yanaenda, kuna lami kwenda moshi ni just half an hour kwa hio kuko vizuri.

I1 nakushukuru sana.

HM-2

I1 Nashukuru kwa kunielewa vinginevyo (hasikiki vizuri )ningeshindwa kufuata ….. (wanacheka)

R1 sisi wote watu wa Tanganyika bwana

IQ sasa kwa wafanya kazi wa idara ya afya hapa wilayani, mwajirir hasa ni nani?

RR mwajiri, mkurugenzi ndo mwajiri

IQ kuna wafanyakai ambao labda wameajiriwa, wamchanganyika changanyika wameajiriwa labda na wizara, wengine wameajiriwa na mkurugenzi, wengine wamea jiriwa na mashirika ya maendeleo kama labda GIZ au …. Watu kama hao wapo? Ukiangalia kwa ujumla?

RR nadhani wizara ya afya hapa kwetu (ndio), wanasoeshwa na wizara hao watu, wakisha maliza wakionekana kwamba wamsha qualify, wizara ina post kwenye maombi; wanapostiwa na wizara kutokana na maombi yetu. Sisi tunaomba hao hao wafanyakazi, post chache. Wakisha post kwa mkurugenzi, sasa mkurugenzi ndo anafanya utaratibu nao, yeye ndo anekua mwajiri wa hawa watu, na pamoja na haja ya ajira, anapeleka mkurugenzi, sio kampuni

IQ nashukuru sana, nimependa swali hili, kwa sababu kuna mpango flani au mkakati flani ulipitishwa na watu wa MATROLDA???? Na sometimes watu wa GIZ wanapromote kutoka huku, wana wa sponser kusudi wakimaliza wanakuja moja kwa moja na inakua labda njia rahisi a wao kuaki katika vituo, sijui kama hilo lipo au unafahamu kuhusiana na hilo?

RR kuhusiana na hilo ni kwamba, haya mashirika hayo unayo yafahamu hao wadau, wanachangia, kwa mfano mzima, walituletea vijana ma CO wanne na katibu washa??? Mmoja kwa mkataba, wao ndo walikua wanawalipa mshahara. Baada ya kumaliza mkataba wao, kama wanataka kubaki hapa, wanatakiwa wafuate channel kama zile za watu wengine wanaokuja na sis indo tuna wa absorbkatika kituo, vinginevyo mkataba ukiisha na wao wanaondoka. GIZ hakuna hata mfanyakazi mmoja alioko wilayani kwetu hapa (ndio) wengi ni waajiri tunafanya nae kazi hapa; kalukula. Tuna wafanyakazi watatu hapa wilayani ambao wapo kwa mkataba na EXPAC MAABARA CLERK, maabara clerk mmoja yupo Msongwa, maabara clerk mwingine yupo chingi mwingine watso hao wana mkataba na GIZ bado, basi.

IQ Asante sana, umeniambia mkurugenzi ndie anehusika (yeah), bila shaka kunakua na mchakato mpaka muajiri kuona kwamba kuna mahitaji, ue mchakato ukoje? Wa kuajiri watumishi wa idara ya afya wote.

RR kuna kitu kinacho tumika, kwamba halmashauri kila mwaka lazima iajiri wafanyakazi idara tofauti (ndio), sasa watu wanakaa na kuangalia uwezo wa halmashauri, uwezo huo upi? Na kila idara ina angalia ina mapengo sehemu gani. Kama matatizo ya pengo yapo huku huku, ina angalia alafu mkurugenzi ana angalia, je kila idara ina uwezo wa kupangisha wafanyakazi wangapi, badae ndo tuna compile, tunaweka kwa pamoja kwamba labda idara ya afya tuna hitaji madaktari hawa, wauuguzi hawa wa ngazi hii, ngazi hii, ngazi hii. Tukishamaliza hayo, sasa mkurugenzi anaweka kwa pamoja alfu anapeleka utumishi. Kule utumishi wakishakubai kwamba hawa kweli watu wana mapungufu sasa hivi, huyo mtumishi anapeleka wizara ya afya. Wizara ina waamrisha watu wa wizara ya afya kwamba wilaya ya kilwa, wapelekeni watu hawa hawa hawa, na hao watu ndo wanakuja huku kwetu baada ya kua wamesha tumwa hapa na wizara.

IQ asante mheshimiwa, lakini pia kabla ya kupata wat, huko chini sasa, kabla ya kufika kwako maana mnapeleka kote (mhhhh), ni mchakato gani mnao ufanya.

RR Ahaa, tunakaa, ile kamatii ya uendeshaji, kamati ya idara ya afya tunakaa tuna angalia ma gap yako wapi, (………………) tuna angalia mapengo yako wapi, baada ya kuonna mapengo yalipo basi tuna omba kwa mkurugenzi kutokana na mapengo yale tulio yaona. Lakini sasa inawezekana mkawa na uhitaji wa wafanyakazi ngilakini uwezo wa halmshauri au wa mkurugenzi wa kuwachukua kutokana na budget ya afya, ya sekta ya afya, na idadi hio ndo ambayo tunatumia.

IQ utaratibu wa kusahili wakati una agiza, usahili unafanyikaje? Nadhani (…………..)

RR wakati wa ajira (ndio), mtu anapokuja kutoka ….. wizara wizarani, kutoka wizara ya afya, wanapotuma wafanyakazi hapa, aaaah, wanaomba kibali kutoka kwa mkurugenzi, mkurugnzi anapokua na ile barua, yeye ana idara yake inayo husika na maswala ya utumishi, ambayo ni idara ya utumishi, kwa hio afisa utumishi anaitwa na mkurugenzi na kukabidhiwa huyo mtumishi, kwa hio usahili na kuangalia makaratasi yake kwamba kweli ndo yenyewe na wamekwisha ona kwamba kweli yako sawa ndo yana ingizwa kwenye mfumo, kwa sekta ya mfumo nusu kufahamu kila kitu kufahamu aisha yake yote, ya shule na mengineyo, yakisha ingizwa pale ndo yanatumwa utumishi. Utumishi wakiona yanafaa ndo wanasema huyu mtu anaweza kuajiriwa, wakiona hayafai, wanarudisha kwamba mbona hapa hiki cheti kipo hivi na hivi au mbona labda hana hiki na hiki, kitafuteni hiki, kinatafutwa, kikisha patikana na wale wakisha kubali ndo ana ajiriwa, vinginevyo hawezi kuajiriwa. Imetoka hivyo kwa kijana mmoja wa maabara (ndio) nafikiri miaka miwili iliopita, alikuja hapa alikua hana nanilii ile, kiongozi ya usahili kama yeye anaruhusiwa kufanya kazi, shule kamaliza lakini hana kibali cha kufanya kazi, uumishi walimrudisha. Baada ya kumrudisha wizarani, ikabidi aende kule atafute, akapata ndipo akarudi kwetu akaleta, ikawekwa kwenye (…………) ikatumwa kwetu tukafanya matengezo, sasa hivi ndivyo system ilivyo kaa

IQ je, katika kutafuta waajiri/wafayakazi, watumishi katika idara ya afya kuna maeneo mnayapa vipaumbele? Labda cadreambazo mnazipa kipaumbele kwa hapa kilwa?

RR kwa kweli cadre ambazo tunazipa kipaumbele kwa hapa kilwa ni madaktari na wauguzi, madaktari ni wachache, na wauguzi ni wachache kwa hio tuna kazia sana kupata hao watu. Mimi mwenyewe nimetumwa kutoka wizarani kwa jaili ya kuja kufuatilia hizo cadre, ili tuweze kupata kwa wingi kwa sababu tuna upungufu takribani mkubwa, tuna vituo vichache vinaendeshwa na madaktari, I mean hao ma CO hawatakiwi kuviendesha lakini kwa sababu tuna uhaba, tunatakiwa tufanye hivyo

I1 lakini dokta nitakuomba takwimu ya wafyakazi wa wilaya hasa kwa upande wa idara ya afya.

R1 nafikiri unaweza ukapata kwa katibu, katibu ndo anaweza kukupatia hivyo vitu

IQ labda nisogee kwenye ile swali la namna mnavyo wakaribisha wafanyakazi waajiriwa wapya wa idara ya afya, sijui kwa uzoefu wako. Kwa uzoefu wa miaka mitano wewe umeshapokea kadhaa, kwa kawaida kuna utaratibu gani wa kuwapokea hao watumishi wapya?

RR kwa utaratibu uiokwepo (ndio) watumishi wapya wanapo fika, baada ya kuweza kuprocessiwa utumishi (ndio) wanapewa barua kuja ku repoti kwenye idara wanakua chini ya mkurugenzi. Mkurugenzi akisha malizana nao, sasa anawaandikia barua yak u ripoti kwenye idara yetu. Wakisha fika kwenye idara yetu,sisi tunawafungulia file kwenye idara. Tukisha wafungulia fie, ndio tuna waambia, tuna kaa nao kuwa ambia, kuwaeleza kwamba kwenye idara yetu kuna vitu gani anapaswa avifuate au avifanye, wakati wa kiwa hapa. Wengi wanakua wametoka toka shule, wachache wanakua lakini vili vile inabidi tuwape miongozo ni vipi wanatakiwa kuishi, aishi vipi na jamii, afanye kazi vipi, anatakiwa afanye kazi vipi, na haki zake azipate vipi. Na moja ya haki zake ambazo anapaswa kupata ni ile allowance ya zile siku ambazo atafika hapa kabla ya mshara wake. Kwasababu pamoja na mtu kwamba hajui, ni lzima aelekezwe, anaandika, akisha andika basi inategemea kama ile pesa ofisini ipo ama haipo, kama ipo ofisini akisha andika, ndani ya wiki moja anapata pesa yake lakini kwa bahati mbaya kama ofisini ile esa haipo, inabidi avumilie kidogo.

IP hakuna utaratibu wa kukopesha? Au kukopeshanyie mnakuchukulia vipi?

RP kukopesha vipi?

IR kukopesha labda kama ofisini hakuna, ana survive hivyo.

RR unajua (watu wanaongea nyuma) (……………..), unaweza kukopesha kama ipo, sio kwamba unakopesha, ukaipate wapi? Inakua ni ngumu. Kitu ambacho tunakifanya kwa wafanyakazi ambao wamekosa ile posho kama sisi hatuna pesa ya kumlipa, basi tutamuomba akapumzike kwanza nyumbani. Pesa itakapo ingia ndipo anaripoti, kwamba hii inamkuta yupo kazini. Ila nimeshangazwa, tangu uongozi huu, wa awamu ya tano umeingia madarakani, wametuletea wafanyakazi, ilikua ni huu mwezi wa tatu, wafanyakazi wengi tu wametuleletea na hela yao, wakati ilikua haijatokea hata siku moja, na hela ya watumishi, tunashukuru, walifika tukawapokea wakachukua hela yao.

IP siku ile nimefika hapa uiniambia kuhusu hilo,

RR eeeeh, ilikua kama ni bahati kwa namna moja, lakini wengine kutoka kwenye mazingira hayo hayo. Tumesha waandikia sana kwamba wanafanya makosa (kama walimu) ndio. Wanapompost mwalimu kwnda wilaya flani, wanatanguliza pesa zake za kujikimu, lakini huku idara hii, huku hicho kitu hakipo. Kwa hio ilikua mara ya kwanza safari hii kwa hawa madaktari waliokuja (hapa ndo kazi imeanza) tuombe Mungu, wakendelea hivyo hivyo tutashukuru Mungu. (vinginevyo watu watakuang’ang’ania na cheque kwamba hatuna pesa za kujikimu). Lakini sisi tuna afadhali wanapokuja, tuna nyumba za kupokea wafanyakazi kipindi wanapofika. Akifika anakaa kwenye zile nyumba, akisha kua tayari kutoka kwenye zile nyumba akakae kwake, ndp anapo toka kwenye ile nyumba akakae kwake. Hilo ndo tunalo furahi, hakuna mfanyakazi anaefika akakosa mahali pa kukaa hapa, haiwezkani.

IQ vipi kuhuu utamaduni namzingira ya kilwa kwa watu wanapokuja, utamaduni wa watu kuna watu wanao, kuna utaratibu wowote ambao mnao wa kufanikisha utaratibu (……….)

RR logically ni kwamba (………..), tumetengeneza jina kwamba, wanapo wapa cheo hiki cha kwetu, wakiingia huko mtaani basi moja kwa moja wana fanikiwa maana ndio hao hao wanao fanya nao kazi pamoja na kuishi nao pamoja. Lakini kusema kwwamba kuna utaratibu ambao tumeweka, sidhani kama tumefikia hapo ………………………………………………. (13 30)

IQ mheshimiwa daktari kuna chochote cha ziada unacho weza kuongeza kuhusiana na mchakao wa kuajiri wafanyakazi ukiacha haya ambayo tumezungumza?

RR ahaa, kitu ambacho naweza nikaongeza hapo, ni mchakato wa uajiri, tuna…. Kwa sababu hatuna wafanyakazi katika wilaya yetu, hua tunajitahidi kwenda mavyuoni, kuhamasisha hawa vijana wanao karibia kumaliza kwamba tufike huko mavyuoni kwenda kuongea nao au wale vijana ambao wapo kwenye manispaa yetu kwenda kuongea nao. Tukisha ongea nao, tunawaomba kwamba baada ya mitihani yao waje kufanya kazi na sisi, wanao kubali basi hua wanakuja, tuna watafutia posho wakai akisubiri majibu yake na post, (ndio) tunawapatia posho wanafanya kazi na sisi. Wanafanya kazi. Nafasi zinapo toka, sisi wenyewe tunachukua vyeti vyao na kwenda kuwaombea nafasi kwenye wizara ya afya kwamba apangwe kituo hiki, maana tumesha muwahi, ni wa kwetu. Na hata hawa walio graduate tumeongea nao, tumepata kama watu wa tano mwaka huu. Basi tuna wasimamia vizuri, majibu yao yakitoka, tutakwenda wizara ya afya kwenda kuwachukua ili kuendelea kufanya nao kazi.

IQ ni ka utaratibu kazuri ka kutafuta watu kama hao, DFP wanafanya hivyo, kufuata watu mashuleni na WHO pia, lakini haka ka utaratibu unakaonaje kwa kipindi hiki ambacho wamekua hyper kweli, wanagoma au wanaingia motivated?

RR aaah, hapana, kwasababu kama mmekwisha ongea nae mapema, naye ye amesha kuja hapa, na amepokea, kiashiria kwamba yeye yupo tayari kufanya kazi na sisi. Kwa hio wale wote, ukisha ongea nao na kuwapa , hawana shida. Kuna mmoja yeye yupo pale, ametoka hiki chuo cha KAWA (ndio), yupo pale CO kamaliza. Tunasubiri tu matokea yao yakitoka twende tuka hangaike nae (ndio). Lakini wanakubali.

IQ Labda ni, twende sehemu nyingine. Labda ni mkakati gani wilaya kama wilaya inatumia kuhakikisha kwamba watumishi wa idara ya afya wanabaki kwenye vituo vyao vya na kuto hama hama.?

RR mkakati kama mkakati kwamba wale watu wabaki, hatuna mkakati kwa kweli. Ila ambacho ni kuhakikisha kwamba posho zao wanazipata. Lakini kwa wilaya yetu ya kilwa hatuna uwezo wa kuzuia mfanyakazi asiondoke, angali ingawaje….., hatupendi mtu aondoke….. kufuata kibarua chake ili aje asiondoke inakua ni ngumu sana, siwezi kukuficha, hatupendi waondoke, tunajitahidi kuwapa posho kwa wakati waweze kuzipata lakini sasa hizo zote zinategemea na sehemu ya serikali (yeah). Wengine wanaolewa kutegemea na kama ipo kwenye mawazo yake, kwa hio hukataa ajira. Na hauwezi kumkatalia inategemea, kwa sababu hio inakua ipo ndani ya sheria (ndio) lakini hatuwezi kumkatalia (ndio), ni ngumu sana. Lakini mikakati mingine kwamba tunafanya hiki na hiki (eeeh), hio kweli hatuja jiwekea. Labda hiki kipindi tunategemea mwezi wa saba tuwende kwa wenzetu, wilaya ya ….. kuangaia wenzetu wanakusanyaje mapato na hilo la kuhakikisha wana fanyaje watumishi wasiondoke. Kwa kweli wilaya yetu ni wilaya ambayo collection ya pesa ni ndogo sana. Pato la wananchi ni kidogo kwahio wengi wanao hitaji zaidi wanakwenda kwenye wilaya ambazo anaweza akafanya kazi asubuhi, akafanya kazi mchana, akafanya kazi usiku kwa siku moja ndo wengi wanao ondoka, na hao wanao ondoka ndo wale wauuguzi wenye akili zao timamu, wamesoma shule zao vizuri ndo wanao taka kuondoka kila wakati. Ni wachache madaktari wetu wanao amua kuondoka kuwafuata wake zao lakini kwa madai ya kwenda kuongeza kipato, hakuna

IQ hakuna mkakati wowote labdda katika kuwa prevent wauuguzi, wanapata fursa ya kujiendeleza kwa maana ya …

RR kwetu sisi huo mkakati upo, tumeshawapa opportunity kwa ambae anataka kusoma (ndio) awe anasema na sasa hivi nilisha ongea na wauuguzi kwamba nikiona anependa kwenda kuchukua mafunzo ya nusu kaputi KCMC au Muhimbili, au Ifakara, aniandikie barua tumpeleke kule akasome. Hio, hio nilisha watangazia. (na moja ya agenda pia) tulisha watangazia. Kwa fano kama sasa hivi kuna ma CO kama 6 kidogo sina; am not sure, kutoka ma CO hapa, mmoja tayari amesoma akamaliza lakini bado cheti chake (ndio), lakini wapo wengi wanasoma . kwa hio tunapenda huko badae tuwe na madaktari wa kuzalisha hapa wenyewe wengi, alafu madaktari kama madaktari wanao kuja hapa na kufanya kazi kama inavyo takiwa, hawakubali na bahati mbaya tunaweza tusipokee zaidi (IP) ni kweli tunaweza tusipokee zaidi, kwasababu tuipangwa kama watu sita wakati tunakuja huku, lakini nikajikuta nimebaki peke yangu, wengine wote (wakatawanyika) waka kataa. Hakuna daktari atakae penda kukaa huku kwasababu hapendi kuishi na kufanya kazi hospitali peke yake. Anataka akifanya kazi akitoka pale aende kwenye kioski kingine (kwasababu…) lakini bwana akiona hapa hamna hicho kitu, unaishia hapo hapo ulipo (kwa hio ujuzi anao kwa hio angeweza kuuza kupitia hapa), hio ni tatizo

IQ Daktari, kuna swala jingine linahusiana na upokejai, jamii inawapokeaje watumishi wapya. Kwamfano kama wamepata apartment na wanataka wajifunze kuhusiana na jamii. Kuna utaratibu wowote wa kuwapokea watumishi wa afya freshers watakao waona kwa ajii ya huduma. Wewe swala hili unaweza kulizungumziaje?

RR jamii, maana jamii ya huku bwana ni ya watu ambao wana mila zao ni watu ambao wana mila zao, na anapo fika mgeni na kama hawajamzoea, ni ngumu sana kuishi nao, inakua ni ngumu sana kuishi nao na wenyeji wa huku

IP kwa nii ni ngumu dokta?.

RR inakua ni ngumu kwasababu tabia zao na mila zao zipo tofauti na watu wengi, na wageni (ndio), umeona eeeh. Kwa kweli ni ngumu sana alafu huku uumini upo sana, uumini upo sana, wakimuona mtu ambae hajafikia huko kwahio sio mwenzao ni ngumu sana kumpokea kwenye jamii. Lakini kama ni mwenzao wanaoweza kuswali nae inakua ni rahisi sana kumu accept haijalishi kama ni mtumishi wa sekta ya afya. Ipo sana. Kwa mfano ule mda wa ramadhani, hakuna seeu yoyote utakapo nda ukakuta chakula kipo, sehemu zote za chakula zinafungwa (interview kashangaa), ndio kunafungwa hata watu wanaofunga wanakimbia (kunakua kama Zanzibar), hivyo hivyo kama Zanzibar, kwamba hakuna cha mgawawa hakuna cha nini. Umeona eeeh, ni ngumu sana kuishi huku ….. labda kwa sisi wengine ambao tumezoea.

IP na mazingira haya ni magumu zaidi vijijini au ukija …

RR ni wilaya nzima, wilaya nzima

IQ alafu swala jingine nilitaka nijue ni vitu gani ambavyo vinasababisha ama vinachochea, kwanza tujue kama wafanyakazi, tunajua kwamba kuna wafanyakazi wanahama wale ambao wanashawishiwa na rafiki zao ambao ni wauuguzi.na kiasi flani tunajua kwamba mtu anatoka hapa kusudi aende mahali ambapo motivation ya pesa ipo, kusudi asifanye kazi kituo kimoja. Kuna sababu nyingine labda ambazo wewe dokta unazifahamu zinazopelekea wafanyakazi wa afya kuhama kutoka hapa kwenda sehemu nyingine

RR sababu nyingine, wilaya ya kilwa ni wilaya ambayo inaogopeka sana kuliko kukubaika, mambo ya uchwai, watu wana ogopa. Mimi mwenyewe natoka huku lakini wakati nakuja huku niiambiwa wewe huku kuna uchawi.

IP ndio, hata miminimeambiwa kwamba hapa kuna hayo mambo ya uchawi uchawi,dokta inatokea hio?

RR eh bwana sana, nikwambie … kule veta mtu mmoja, wao kwa wao (ndio) alikua ameenda sehemu aka kaa sasa kwa bahati mbaya akawa amedondosha pesa yake (ndio) na wamekaa kabisa nawalipo ondoka, mwenzake akachukua ile pesa akaweka mfukoni. Alipo ondoka akakumbuka kwamba pesa kadondosha (ndio) akarudi pale akauliza samahani kuna pesayangu nimedondosha umeiona? Hatujaiona. Akasema kama hiajonekana basi, itaonekana baada ya siku moja mbili, yule mtu baada ya siku moja amekufa yule aliechukua zile pesa, anaongea kuhusu zile pesa kwamba kwanini umechkua pesa zangu (ndio) hio ipo, ni mbaya sana. Kuna nyingine tena ilitokea, kuna mtu mmoja duka lake hafungi (wanacheka), siku moja Yule bwana, yeye ni mfanya biashara kwa hio alikua ameenda kufanya kazi zake, akakamatwa, polisi wakamkamata, walipo mkamata wakampiga pingu, akawa anawaambia polisi jamani hata zangu. Kufika hapo kituoni alipo kua OCD akasema “mfungue huyo kijana pingu”, jamaa akasema “aah, tuna mfunguaje huyu kijana pingu?” akaambiwa wewe hii kesi peleka mahakamani. Yeye kijana anasema jamani mimi sio mwizi. Jamaa aka … Yule OCD akakubali kumfungua zile pingu, akatoka kwenye pingu “asante, lakini mmenishika kwa kosa ambalo sijalifanya”. Yule jamaa kesho yake, alikua akiingia tu kazini ana … ndani ya wiki moja jamaa aliondolewa hapa kilwa, kaja kuondoka. Mwingine TRA hapo …………………………………………………………………………………………. 26 kwa hio mtu anapo kuja huku, akisha simuliwa , hapataki na wala harudi tena

IQ sasa dokta hao watumishi wanao hama hama, wanahama kutoka vijiji vya kilwa kwenda mijini au wanahama kwenda wilaya nyingine tu iwe ni mijini au ni vijijini?

RR wengi ni mijini, wachache sana wanao amia kwenda vijijini

IQ na kwa uchunguzi wako au kwa ufahamu wako wanahamia kwenda vituo vya serikali au?

RR vya serikali, anaomba kama kawaida, mwaka huu kama wawili wameomba waondoke.

IQ na kiwango cha uhamaji unaweza ukakipa asilimia ngapi kwenye…?

RP kwa mwaka au kwa wafanyakazi wote?

IR yeah, kwa wafanyakazi wote, lakini ibase walao kwa mwaka

RR tunapata watumishi wapo, wachache sana wanakuwa hawapo, lakini wengi wanapo hama, mtu unajua mtu, kwa mfano mkuu wa mkoa aliepita (jina la mkuu wa mkoa) alikua amesema hivi, “ wafanyakazi, mkoa mzima una shida ya wafanyakazi, tusiruhusu wafanyakazi kuhama. Kama mtu anataka kuhama, anaruhusiwa kuhama ndanii ya wilaya sio nje ya wilaya au ndani ya mkoa, sio nje ya mkoa .” baada ya kuichukua hio na sisi tukaifayia kazi hapa. Tukaambiwa angalieni walimu, walimu wale wakitaka wahae wanabadilishana na mtu mwingine, tukaambaiwa hata sisi idara ya afya tunatakiwa kufanya hivyo hivyo. Sasa mtu unamkatalia kuhama, anatoka hapa anakwenda mkoani, akifika kule mkoa, wale wa mkoa wanakupigia wewe simu, kwa nini hutaki kumpitisha huyo barua yake ya kuhama. Ukimpitishia hapa, akienda kule mnaambiwa kwa nini mna pitisha wwat wengi. Kuna mtu kaolewa, anataka kuhamia rufiji, kwa hio amekuja hapa kutaka kuhama, mimi nimemwambia kwamba atafute mtu abadilishane, hatai. Kwa hio tuna changamoto kubwa, changamoto kubwa. Huku unaambiwa hiki, huku unaambiwa hiki, changamoto ni kubwa. Lakini sasa utafanyaje, ndio maisha. Lakini kwa kweli tunategemea sasa hivi kwa mfano mwaka hu tangu nimeingia, tuna wafanyakazi ….. moja, mbii tatu, wafanyakazi watatu wamehama. Wafanyakazi watatu wamehama na tuna wawili hapa ambao ndio barua zimefika (ndio), kwa kipindi cha mwaka huu.

IP na sababu ya hawa ni tofauti tofauti?

RR sababu ni tofauti tofauti. Mmoja t undo alipata mt wa kubadilishana nae, akahamia mbeya, lakini wengine wanakwambia, mama anaumwa ni visingizio tu, mara nataka nirudi kwa wazazi. Lakini watu hawataki kukaa huku (kiti kinasogezwa), mwingine naumwa, kwa mfano, kuna mmoja hapa ameniletea barua kwamba yeye ana hypertension (ndio) , ana hypertension kwa hio ameambiwa ahamie karibu na hospitali…. Kubwa ili aweze kuhudumiwa kila baada ya mda mfupi. Daktari katoka muhimbii kule kwenye masomo yake, sasa mimi nikawa namwambia paoja na kwamba wame diagnose kwamba una shida hii, hakuna sehemu ambayo ume convince kwamba unatakiwa uhame mkoa. Ndo sasa ukijaribu kukataa mwingine anakua hakuelewi, ndo mapambano hayo sasa

IQ sasa tuangalie jambao jingine hapa, jamii ina mikakati yoyote, jamii hii ya kiwa, wana mikakati yoyote ya kuhakikisha kwamba watumishi wa afya wanao tmwa au wanao wahudumia, wana baki hapa kilwa kwenye vituo vyao wasiangaie vituo vingine?

RR kwa kweli jamii it plays a big role, ndo maana umesema ni jamii, wakazi wawilaya hii wageni hawataki, labda mwenyeji watampokea sio wageni na hii itachukua mda mrefu sana kubadilisha hawa watu. Wakuu wa idara watatu kupitia madiwani, wamewakataa kwamba hawawataki, na waondoke hawatakiwi kukaa wilaya yetu. Wakuu wa idara hao watatu, afisa utumishi akawaambia watoke kutokana na hao hao madiwani, leo mtu wa serikai anasema hivyo, kwamba tume kaa tumeangalia hivi hivi hivi. Hao kwenye jamii wao wanataka kwamba ndugu zao ndo waje wafanye kazi, wakati huo huo, ndugu zao hawana shughuli hio, kwa hio ni ngumu sana. Sasa hivi tnatakiwa angalao kwwenye kila kijiji kuwe na zahanati, lakini sasa unajenga kajengo kakutolea huduma, hawataki kujenga nyumba ya kukaa mtumishi, wanataka ukisha jenga jengo uwapelekee mtumishi atakayo kaa,ndicho wanacho penda lakini uwatafutie mtaaamu uwapelekee, hicho kitu hawana. Serikali ilisema kila kijiji wanze huo mchakato wenyewe alafu serikali itatafuta namna ya kuwajalilizia, lakini sasa ukisha weka kajengo tu, wanakataa nyumba ya mfanyakazi, unaona eeeh. Ahaaaa

IQ hapo hapo, inahusiana na hilo swala, unasemaje kuhusu uimara wa hizi kamati za afya kwenye ngazi za kata au kwenye ngazi za vijiji?

RR aaaaah, uimaraaa upo, sisi tunawajengea, tumefanya hivyo kama miaka mitatu, sisi tunawajengea uwezo kuwafundisha ili wawze kujua wanapaswa kufanyaje (ndio) yeah. Kwa mfano sasa hivi imeshavunjwa, kutengenezwa kamati mpya, wamesha andika barua ka hio watu wameshachaguliwa, tuwajengee uwezo, waelekeze, wanatakiwa waje lazima tuwaambie wanatakiwa kufanya nini

IP aah, kumbe mwaka huu ni mwaka wa uchaguzi, kwa maana ya kamati

RR kamti, vile vile tunabadilisha kamati kwa mfano hata bodi ya afya, imesha chaguliwa mpya tayari, na tunategemea ijumaa watakua wamepatiwa kazi ya kufanya.

IP kwa hio, kata zetu hizi, kamati hazipo? Au zipo zile za zamani, kama zimevunjwa manake hazipo.

RR zipo, tulisha fanya mchakato, mchakato ulisha isha (ahaaa) yeah, inategemea na unawaharakisha vipi. Tumesha anza kupata taarifa kwamba huku tayari, huku bado, (ahaa) na tunawahamasisha kila wakati, tunawapigia simu, hawa wajumbe wa kata (ahaaa) na ninafikiri ijumaa hio tukisha kamilisha hio bodi, sasa bodi itakua tayari kwenda huko kwenye makata na kuona na kuwajengea uwezo (okey). Wale ambo bado tuna washinikiza wakamiishe.

IP hii taarifa pengine itanisaidia mimi, hivi hapa masoko kuna kamati ya kata tayari?

RR hapa masoko, bado bado, sidhani kama imeshakua tayari ningekua nimeshauri bwana kwamba tumuulize kama tayari au bado. Tumeshapokea barua rasmi hapa, lakini mganga mkuu wa kituo nilikua nagombana nae hapa, anasema kwamba akienda kule kwenye kata anaambiwa bado, bado.

IP kwa hio watumishi ndo wana …

RR wananisumbu sana, wananisumbua sana hawa jamaa, kwa sababu kila anacho kuambia anataka pesa (yeah)

MAONGEZI YA SIMU

DMO Hallo, eeh bwana kamati ya afya umesha ipata bado?

_Naam

DMO Kamati ya afya ya kituo chako, imeshapatikana?

_Bado

DMO Kwa nini mpaka leo bado? Huyo mtendaji anafanyaje?

_Mtendaji kwa kweli hata mimi nashindwa kumuelewa, tumeshaenda, katibu na yeye ameshaenda, baada ya hapo ananiambia kwamba kamati ile irudiwe

DMO Mhhhhh

_kwa hio hapata kua na barua

DMO barua ile ile kutoka kwake?

_Eheee

DMO baasi wewe mtafute kesho, mwambie kama yeye hataki ile daily … aniandikie barua na uje uniambie na mimi nimfuate, kwasababu kama tuna dorora hapo, pesa hazitumiki, umeme hatujalipa kwa sababu yake, inakuaje?

_sawa

DMO sawa,kesho nitaomba majibu hayo rafiki yangu .

_sawa

DMO haya, asante, asante, asante.

RR hao ndo wenyiji wango, unaona jinsi wananisumba, unaweza kapeleka huku, peleka huku (mambo hayaishi)

IQ lakini dokta ukiangali kwa mda hata hizi kamati ambazo zilikuwepo, walikua wakifanya michuano yao ya mara kwa mara labda kugagua mahesabu?

RR michuano hua wanafanya, huwa wanafanya. Dawa zinapofika, lazima wazipokee (ndio) na tunapo taka kutumia hela yoyote ya hospitali, lazima kamati ikae kwa ajili ya muhtasari na kila kit undo mpewe.

IQ dokta ukienda kwenye hizi kamati au bodi za usimamizi za vituo hivi, ama hizi kamati za afya ambazo zimechaguliwa zisaiide kama hizi za kata, zina fanya kazi?

RR kamati zipo, kamati zipo. Ila tu haya matatizo ya mda mrefu, tunasuguana.

IQ yeah, aaam, natakiwa nirudi nyuma kidogo kwasababu (anacheka), lakini kwa kweli nakaribia kumaliza. Nataka tu niulize changamoto zinazo ikumba mikakati ya kukabiliana na hawa watendaj. Awali tulivyo kua tukiangaia ile mikakati ya kuona kwamba, miakakati ya kuhakikisha kwamba watumishi, tukaona kwamba hakuna mikakati formal, ni ile tu ya kuhakikisha kwamba watumishi wanapata posho zao lakini tunaona kuna tendency, wanakuja wanapata mahali pa kufikia, ni moja ya mikakati ya hawa watumishi kwamba hawa hami. Na kuna changamoto gani ambazo mnapambana nazo katika kuweka mikakati yenu ya kuhakikisha kwamba watumishi awahami. Kuna ambayo umeshataja kuhisiana nah ii kuhama, kwa mfano; hawataki kukaa kwenye kituo kimoja, kuto kuwiana na kukubaliana na wenyeji, nataka tu nijue kama kuna changamoto nyingine?

RP za kufanya watu wasihame?

IR changamoto ambazo mnakumbana nazo mnapo sema kwamba okey hatutaki watu wahame hame hapa

RR changamoto zipo nyingi (ndio). Cha kwanza, changamoto kutoka mikoani , Napata changamoto kutoka mkoani. (ndio, changamoto nyingine) aaaaah, kuna priority kutoka juu kabisa, kwamba huyo mtumishi aondoke, unafanyaje sasa wewe kama wewe? Na Yule ni mtumishi wa serikali, huwezi mzuia asiondoke, ukikataa hapo unafanyaje? Huwezi kukataa (wanacheka) inabidi tu ukubali, anaondoka. Changamoto nyingine kuna viongozi hawa wanasiasa hawa (ndio) changamoto vile vile kwa kazi yangu, unaona eeh, na changamoto nyingine, haya mambo ya kishirikina, akishasema kwamba bwanamimi huku siwezi, utafanya nini kwasababu kama hutaki, naacha kazi naondoka. (inabidi uyaishe mambo yaende) eeeeh, mwingine anakuja kukuambia kwamba mimi hapa hawanitaki, natukanwa. Kuna mdada mmoja alikuja hapa walikua na mama mzee kipindi hicho, kwa hio wakatuambia sisi huyo hatumtaki, tunamtaka mwingine tutakae mpenda. Sasa hospitali yenyewe inawafanyakazi wachache, na wao hawamtaki, kwa hio yue mtu aka amua kuondoka. Changamoto nyingine ni kwenye hali ya maisha, chakula hapa ni shida jamani, vyakula vingi ni vya kununua. Wilaya inaagiza vyakula kutoka nje, wafanya biashara hao wanachukua chakula kutoka nje ya wilaya, watu wa huku hawalimagi kwa sababu tunapo kaa huku ni mlimani, zao kubwa ni kahawa, wanangoja tu uvunaji …. Sasa mtu anashindwa kukaa kwasababu hakuna sehemu au njia ya mtu kuongeza kipato, ni changamoto kubwa. Ina fanya mtu akatishe tama kukaa hapa. Wengine wanataka mawasiliano, unawapeleka vijijini kule, hawawezi kuwasiliana na ndugu zake kwa hio anaamua tu kuacha kazi, ana ondoka. Mtu mwingine amezoea umeme, umeme ni wa shida, vijiji vingi havina umeme, ukimpeleka kule atataka kuondoka, atatafuta tu sababu yoyote aondoke. Na angalao saa hivi bara bara ime kubali, hii bara bara hii tumeandaa kutoka hapa, kulikua na kilomita mbili tu hazijaisha ndo zimeisha mwaka huu. Kabla ya hapo sisi tulivyo kuja haoa, ukifika tu, bara bara ya vumbi (……….) (yeah na watu wengi wanafikiri mpaka walipwe) wengi tu wamekuta bara bara ipo hivi, kwasababu zamani ilikua hamna vyombo vya usafiri hapa. Kwa mfano kuna mmama mmoja alikua anatoka dar kwa bus, amepata mimba njiani alafu akajifungua ndio akaja huku. (wanacheka) sio kwamba nakudanganya ndivyo hali ilivyo kua huku. Kwa hio hata wamama wengine wanafika wanakuta bado mambo ni yale yale. Changes zipo na zinaendelea kuwepo kama nilivyo kuambia

IQ na kama ulivyo tuambia kwamba watumishi wote wa afya wana ajiriwa na mkurugenzi, isipo kua wachache tu ambao wapo chini ya miradi, kuna tofauti za changamoto kwa hawa watumishi? Kwamba huyu kwa sababu labda yeye ameajiriwa na, anajiona kwamba yeye ni bora zaidi kwa hio kule trend ya kuhama labda ni kubwa zaidi tofauti na hawa alio ajiriwa na halmashauri?

RR haijalishi sanakwamba, kwasababu wale wote ambao wameajiriwa zile sehemu za EXO wote ni wenyeji wa hapa, kwa hio hamna mtu ambae atakua anafanyia labda DFP atahama, wengi wanao hama wanakua ni, Wakule ndo wanahama wengi. Lakini wengi wa hapa anakua ana fanya kazi kama kawaida na nafikiri nimekuambia kwamba nina wafanyakazi watatu tu (ndio) ndo wameamua lakini si ajabu huko njiani waka amua kurudi serikalini hapa.

IQ na dokta kuna mikakati mingine mipya ambayo mnaplan kuja nayo kuwa retain hawa wafanya kazi wasiondoke?

RR mikakati mipya ipo, ni kwamba, unavyo ona halmashauri kama halmshauri (ndio), nimesha ongea nao, wanatakiwa watoe 10% ya makusanyo ya own source. Ili basi iweze kutujengea uwezo wa kuwa retain hawa wafanyakazi wapya. Kwa hio tunasubiri mwaka mpya wa fedha tuone, kama itawezekana, kama ikiwezekana basi haya mambo tutayafanya ili hawa wageni wanapo fika na wao waone basi kua wanaweza wakaishi.

IQ labda mwisho kabisa, ushauri wako tu kuhusiana na tuicho sema ili…

MAONGEZI YA SIMU

DMO halloo

_hallo, habari

DMO salama tu, habari za kazi?

_(hasikiki vizuri)

DMO mhhh, nakusikiliza, nakusikiliza bwana

_ nipigie baada ya mda

(kelele za mawimbi ya simu)

RR samahani kwa simu, mtu amekuja na kitu chake, alikua anakuja, amepata traffic, amepata accident akapatumia lifti sio kwamba atachukua basi la shilingi elfu moja na kumleta hapa. Sasa ametaka kurudishiwa naui anataka kupewa elfu hamsini yake ya kuja na elfu hamsini yake ya kurudi, wanakataa. Kwa hio kwenye hii sehemu inakuja swala jingine la haki, la haki, unaona eeh. Kwa hio hivyo ni vitu vingine vinavyo takiwa kuonekana kituoni ili kuweza kuwasaidia hawa watu.

IQ na labda mikakati fursa ambayo unaiona inaweza ikatoka chini huku katika jamii ili kuangalia hilo kwa ujuma wake.

RR kwa jamii kwanza inatakiwa wapokee watu, hicho tu. Wakisha wakubali wenyeji (ndio) na wakawaongoza wakaingia wakaacha mambo yao ya kitamaduni wanaweza wakakaa. Kuna jamii tena, wanatakiwa waone kwamba hii wilaya sio ya maskini, ni wilaya tajiri sana ingawa wanalipa peke yake, bei ya chakula (ndio) wanatakiwa waweke sehemu za kugawa chakula, ili wafanyakazi hawa wapate chakula kwa bei nafuu. Chakula hapa ni gharama kubwa kubwa, kuku hapa ni shilingi elfu kumi na tano ndo unapata. Kuku hawa wa kisasa, hawa bei rahisi kuliko hawa. (mhh, itakua ni rahisi) sasa ni nguu mtu kukaa hapa, sasa jamii lazima iangalie ni vipi inaweza kukaa na wageni . kwa sababu bahati mbaya jamii, hakuna wasomi wengi wanaopenda kuja kufanya kazi huku na hawana wasomi. Ndio maana wametaka vijana wao wachukue fedha kwenda kusoma shule, vijana wenyeji wa hapa, na wanapata, ila dedication, hakuna , kwa hio wasomi ni wachache sana kwahio wengine waliobaki wao wawasaidie kwa kuja kuwapa pesa zao, sio pesa zinatoka srikali tu hazitoki mifukoni mwao kuwapa hao wageni, kwa hio lazima walielewe….. nyie mtakapo kwenda kuongea na katibu watusaidie, hawa watu hawataki elimu, elimu wanayo takiwa kupata ni elimu ya chuo kikuu, sio (yeah) na ukisha waambia wanasema tuondoee siasa. Lakini haitoshi kwenda kuwapa elimu hawa jamii, inaweza kubadilika, akini sidhani kama inaweza. Kwa mfano mimi, upande huu walikua wanapenda kukaa na mabusha, mwenye busha mpaka umfuate nyumbani kwake, (ahaaa) eeeh na mabusha ila hawezi kwenda hospitali kwenda kutoa kile kitu. Mpaka dokta sisi tukawahamasiha sanaaa na tukapata shirika moja hivi ndo ukawafanyia… lakini hawakuwahi kuja, walikuja wachache lakini sio wote walio fika. Tulifanya kama operation ya mabusha kama 200 alfu, siku hio tukawafanyia kila kitu, wakalazwa lakini wakaondoka, wengi wale watu. Watu wana imani zao wenyewe, hawataki, labda nyie mtusaidie kuelimisha

I1 nashukuru sana dokta, tumekaa hapa mda mrefu, umechoka lakini umezungumza, mimi siku jua kama ungezungumza mpaka mda huu (wanacheka)

HM-3

I1 rasilimali watu kwenye aaah idara yetu hapa (mlango ukifungwa) ya afya na mmmh imechaguliwa wilaya ya (mlango ukifungwa) hii ya kilwa na kuna wilaya mbili zinahusishwa, wilaya ya kilwa na rombo zote zimechaguliwa kuwakilisha (mlango ukifungwa) (……..) naa zote zinaa tatizo linalofanana <people walking> naa wafanyakazi katika idara hii ya afya huwa hawakai wanaondoka (…….) huwa wanatoa visingizio wanaondoka wengine ni gharama turnover ni kubwa hawahawa. Sasa mjadala wetu huu utajikita hasa kwenye maswala yanayo husiana na wafanyakazi wa idara ya afya<simu inalia> kuhusu kuhama au kubaki kwenye vituo vyao vya kazi.

IQ embu nianze tu kwa kuuliza….. me najua ninyi ni makatibu wa afya lakini inawezekana una wadhifa mwingine tofauti na huu. Wadhifa wako wa sasaivi ni wadhifa gani? Napenda kusikia kutoka kwako……… <kivipi kibinafsi kikazi au kivipi yani > kikazi kwa sababu mimi najua katibu wa vyama lakini inawezekana unawadhifa mwingine zaidi ya huu<…….> inawezekana wewe ni banker wa( …..) je wadhifaa huu unaweza ukawa ni casual? Auuu ( …sijakuelewa) kwa sasa ni health by profession mimi ni(….missing) aaam… na wewe(RR2; mimi ni <…..>) .

IQ; Ni kwa muda gani umekuwa kazini tangu umeeanza, kabla ya kujiunga na wizara au actual kazi sasa ni muda gani?

RR; Nimejiunga na wizaraya afya kwasababu ukiangalia before kabla hawajahamia posta tokea 2002 nimeanza kazi mpaka leo <mpka leo?> mpka leo.

IQ; Naa Kama katibu afya je?

RR; kwanzia tarehe 1-09-2011…<dada yetu?>

RR2; nimeanza kazi mwaka 2009 Kama clinical officer Na 2014 nikawa katibu afya <nashukuru sana>

IQ; naa nilitaka kujua level ya elimu … ambayo mmefikia mmeshatuambia lakini mwishoni, level yako ya elimu ikoikoje?

RR; bachelor of famasia nutrition which is totally nutrition <ivoivo> Na wewe dada yangu?

RR2; level yangu ya elimu ni health system and management <samwan coughing>

IQ; na alikuwepo doctor selinjian flecho mkiwa huko morogoro mkiwa huko ofisini. Sasa tujue ile ilikuwa ni intro tuu ili kuja ku move nakuungana na<…> ya hapa. Ukiangalia, wakati unaangalia wafanyakazi wa afya katika ngazi ya wilaya. Muajiri wa wafanyakazi wa afya katika wilaya ni nani?

RR; district health…. wa wilaya.

IQ; hakuna waajiriwa au wafanya kazi wa afya ambao wameajiriwa labda na eeh idara au kuwa specialist moja kwa moja kwa kutoa msaada au wameeajiriwa na mashirika ya maandeleo labda aaah mashirika maengine ambayo kwakuwa nayo .?

RR; yapo kama Bima, Benjamin William mkapa foundation ambao wanafanya kazi hadi ofisini mpka huku wameisha huku mpaka sasa hatujapata wengine sasaivi so karibu wote kwasasaivi waliobaki utendaji wao ni mbaya.

IQ; aah umesema karibu wote Kwa hao wachache ambao hawaajiriwa wanafanyaje?

RR; Kwa sasaivi Mimi ninavyoona wakiishaa… wote…. Ila unajua hospitali za misheni kama deo zile hospitali za misheni wao kwa utaratibu mkubwa wanawaita help inside mission<talkings>

IQ; nashukuru kwa kunifafanulia lakini pia ningependa kufocus swali hapo tu discuss kutokana na challenges za facilities za serikali………. Binafsi au tradtion… mara nyingi hatuna … labda tuangalie mchakato wakuajiri au uajiri hapa halmashauri ukoje?

RR2; .. anaombea hilo swala la kuwajiri watumishi wa kada ambao wanaopewa kwahiyo huwa anaomba kibali yani mkurugenzi halafu kibali anapewa <tvtalkings> baada ya kipindi hicho wanakuja wanakaa na baraza la madiwani <ndio> ili kama kubariki watu waanze…<mmhh> kwahiyo baraza la madiwani can say yani kukubali na kuangalia kweli kama ndo watumishi tunaowataka baaade, wanatoa mchanganuo watu wanapply wakienda… wakishaomba wanafanyiwa usahili kwa kuongezea kama nilivyosema chuo cha utumishi kimepangiwa wizara ya afya kule wizara kwahiyo wizara zinatoaga matangazo kwamba kuna watu hawa wanahitajika kuomba nafasi za kazi kwa hiyo katika idara ya afya watumishi wote wanaomba nafasi za kazi wizara ya afya. Ndo inajukumu ya la kuajiri watu, kuwachambua labda kama huyu ni MD uyu aende kama kilwa aende wapi aende wapi. Sikuizi kwenye mtandao ma jina yanawekwa kwamba kilwa watu ni hawa kwahiyo wale watu wanasoma kwenye mtandao wamepata kazi wapi na wapi <door closing> kwahiyo wizara ya afya ndo inawagawa watu sasa wakifika kama kilwa sasa mkurugenzi kama mkurugenzi ndo anawaajiri sasa kwa barua za ajira sasa kwamba tumewaajiri.

IQ: ahaa sasa kabla hamjajua mnajuaje kwamba kanda hii kanda hi indo tunahitaji ulemchakato ukoje?

RR; sisi HI<health inspector> wa kijiji nahakikisha umepata sehemu ya makazi sasa unapoenda kumuombea kibali si ndio umezungumzia kibali si ndio<ripoti ikija inasema inataka wafanya kazi wapya kada au kijiji taarifa hizo unazipata kwanani kwamba tunahitaji kadhaa> kwanza sisi kama makatibu na watumushi huwa tunakaa tunafanya hiki na hiki tunajua kwanza tunakuwa tuna cabinet tunajua wako wangapi tupo wangapi wanahitajika wangapi. Tuna kaa tunaangalia katika hii kada wanatakiwa kuwa wangapi … mfano kama watu 20 tunaandika deficit watu 20 ndomaana tunakwambia tunaomba kibali kwamba jamanii wanahitajika watu kadhaa na ikishapitishwa tunaipeleka utumishi kwa hyo utumishi kwamba kilwa kama kilwa tunahitaji watumishi 7<ndioo>

RR2; kwahyo nikupitia ilecopencetric capsel health plan kujua watumishi kada hadi kada

IQ; ahaaa kwa hiyo kwa sasaivi unaweza kufahamu kwamba watumshi wapo wangapi kwa kada ya … iko ngapi?

RR; sifahamu

IQ; tunaweza pata hizo takwimu mwisho?

RR; unaweza kupata.

IQ; wakati huo unaendelea kufikiria hapo tuendelee na swali lingine. Watumishi kama tuseme wamesha letwa na wizara au wameshaakuja mmeshapangiwa na wizara hawa wanakujaa wamefika kwa wakurugenzi? Ni kwa namna gani mnamna wapokea hawa watumishi?

RR; tunawapokea kwanzia tangia day one kwamba wameshapangiwa kilwa wanakuja kwa ofisi ya mkurugenzi watakapo repoti wanarudi wanasubiri kuwekwa kwenye payroll hata kama akisimama kaziwanakudiscover kama unafika. Wanapofika hospitali kwetu tunawapeleka pale kuwapa magodoro wanakaa kwa muda flani baadae wana…

IQ; Ni kwa namna gani mnahakikisha kuwa orientation kuhusu jamii ya hapa ikoje? Kuna kautaratibu cha kuwa orient kuwaonyesha jamii ya hapa inavyoishi?

RR; huwa tunawapa situation ya mambo ya hapa na jinsi ya kufanya kazi kwamba walivyo ni hivihivi na hivi yani tunawaonyesha kwamba jamii ipo hivi na hivi na wakati huohuo … kwahiyo huwa wanalipa kwa katibu wa afya<coughing>.

IQ; kunachochote cha kuongeza kuhusiana na mchakato wa kuajairi wafanyakazi wa afya ungependa kuongezea?

RR: tangia nmekuja hapa?

IQ; eeeh

RR: kuna ….. kama channel wizara kama wizara ni kuleta watu na wakurugenzi wakikubali huenda kuomba kibali cha kuwaajiri watumishi wale ambao wamepangiwa kama watumishi wake. Kwahiyo bila kile kibali yani kile kibali yani kinaruhusu yule mtu kufanya kazi kwamba mkurugenzi anasema nia watu hawahawa na hawa kwahyo ni lazima kile kibalikinaruhusiwa mtu anakuwa aware kwamba kun hayhaya na haya …. Kwahiyo ikitokeaa mkurugenzi akifanya hivyo……

IQ; kwa hiyo kuna maeneo ambayo au kaya ambazo mmezipa kipaumbele wakati wa kuomba kibali cha kuajiri…?

RR; Kwa picha ya haraka <eeeh> ya kwakufirika kwa mamlaka na wakala wa bima huwa tunawapa kipaumbele kwakweli

IQ; kwanini mnatoa kipaumbele kwa hawa

RR; …….

IQ; aahaaa na mara zote hichi kipaumbele kinatekelezwa au hakitekelezwi au inakuaje?

RR; mara nyingi huwa hakitekelezwi kwasababu huwa wanaweza wakaomba idadi flani lakini wakapata pungufu au au isiyo kamilika…<phone ringing and door closing>

RR2; mfano Kama madaktari…. Unaona hawatutoshi kwasbabu ni vigumu sana kutatua…<phone vibrating> uhaba wa watu 47… kwa hiyo mara nyingi huwatunaomba ma Clinical officer lakini chakushangaza kwakweli hii 2014-2015 alikuja mmoja tuu. Mimi nilikuwa afisa wa kati…. Tukakutana na mmoja hukuhuku.

IQ; sasa huwa wanatoa sababu gani kwamba hawapo au hawataki kuja au kuna lawama zozote wanazotoa? Au bado hamjafwatilia?

RR; …..

IQ; sawa nimekuelwaa sasa tuachane namchakato wakuajiri aaah tuangalie kwa wale ambao washafika wafanyakazi wamepangiwa na wamefika tuangalie mchakato au namna ya kuhakikisha kwamba wanabaki katika vile vituo……. Eeeh kuna mkakati gani katika wilaya wakuhakikisha wafanyakazi wao wanadumu katika vituo vyao vya kazi hapa?

RR; mkakati wa kwanza ni kuhakikisha tunawapa motisha ni kuwa lipa … xtra duty na allowance nyingine ili waishi na wakianza maisha yao inawakidhi wanakuwa wanapata moyo wakukaa zaidi.

RR2; lakini pia mkakati mwingine unaotumika ni kuwapatia training au mafunzo ambayo ni ya msingi yanayo husu kazi zao na supportive permission kuona kamana ili wasijiskie vibaya au kuona kuna ugumu unapewa supportive supervison kwa vitendo mpaka anaweza maana wengine wanaona hiki kitu ni kigumu mtu anaelekezwa kwamba ni hivi ni hivi, wengine wanafurahi kwasababu mwingine anaweza akakata tama ya kubaki kwasababu anaona anashindwa kuperfom. Lakini ukienda ukamuelekeza anaona kweli hili jambo ni jepesi.

RR; kinachofanya hili swala hapo nyuma unajua hapo nyuma mtu anaweza kwenda kijijini hapo akakutana na watu wanabeba majani kwa ajili ya nyumba na ukiangalia ametoka mjini kwenye nyumba kali anaona kuondoka mjini ni ngumu. Ndo maana tukakaa tukaongea na hawa Benjamin William mkapa foundation kutusaidia kujenga nyumba ziwe za watumishi. Ambapo watumishi wanapoajiriwa wanaenda vijijini wakaona nyumba Kama hizi zinaweza kuwatia kidogo ari ya kubaki.

IQ; vipi kuhusu jamii upokeaji wa jamii inavyowapokea hawa watumishi wa afya wote pale ikoje?

RR; … pale ni lipo ipo positive kwa mfano kuna … kumpeleka watu wa pale walifurahi sana kwamba wamepata doctor ila in realty ule mkaka kama doctor<door slamming> akapokelewa na mwenyekiti wa kijiji wakamuonyesha yupo salama wakishamfanyia usafi ataenda kuishi kwenye nyumba yake kwa hiyo jamii ilikuwa no problem. Inawakaribisha wote

IQ; ni kwa asilimia ngapi unaweza sema kwmba jamii iko pro active wana appreciate wanapopata watumishi wapya wa afya? Au kadiria tuu

RR; siwezi kukadiria Lakini naona kama wanafurahi wanapoletewa mtumishi ambaye walikuwa wanakosa ile huduma pale kwasababu kuna sehemu nyingine inaitwa ruhaka walipofikia hatua wameleta muuguzi na … walifurahi kweli mpaka wakawa wanamuuliza Yule mheshimiwa diwani aliyewaletea kuwa anakuja lini Yule daktari wetu kwahiyo sehemu nyingi huwa wanafurahia ujio wa watumishi<noise>huwa wanafurahi sana.

IQ; kwasasa tuangalie swala la uhaba ile hali ya uhaba wa wafanyakazi hapa kilwa kutoka kituo kimoja kwenda kituo kingine na ndani ya wilaya au kutoka kilwa kwenda maeneo mengine?

RR: watumishi kama watumishi kutoka kilwa kwenda sehemu nyingine hyo ipo kama walivyohamishwa mwezi wa 7 na 12. kila inapofikia mwezi wa saba na kumi na mbili utakuta wametuma kama watatu na wa 12 watau au wanne hiyo ipo hiyo wengine wanatoka wanaenda mbali kama mbeya kama mwezi uliopita huu sijui wangapi walihamishwa.

IQ; je wananchi wanahama kutoka kilwa kuja maeno ya mjini au maeneo ya vijijini?

RR; wengine ndo mwingine anaomba atoke yuko hapa anataka ahamie maeneo ya kambarage kulee wengine wanaomba uhamisho toka kilwa kwenda sehemu nyingne au mkoa mwingine.

IQ; Na ni vitu gani vinachochea mpaka watu wanahama au wanahama kutoka kituo kimoja kwenda kingine ndani. Kwanza tuanze Na wale wanaohama kutoka kilwa kwenda maeneo mengine ni vitu gani mnaona vinachochea.

RR; vitu vikubwa ni kama ndoa pamoja na unakuta labda mtu ni msichana anakaa baada ya miezi mitatu anafunga ndoa anaanza kusumbua anataka kumfwata mume wake wale ambao wanabarua tunawakubalia wale ambao hawana barua wanaenda kwa mkurungezi wanaomba uamisho wa labda kwenda tanga… hiyo ni sababu ya kwanza .

RR2; saa nyingine huwa hawaongei ukweli yani kweli ndoa nisababu ya kuhama huku lakini utaona kabisa yani huyu mtu hayuko willing kufanya kazi kilwa yani anatafuta sababu nyingi Ili kuhama watu wa hivyo tunaona maombi yao yakopending kwasababu walizozitoa walisema haziridhishi. Kwahiyo ni Kama vile amechoka kufanya kazi kwahiyo anataka asogee huwa wanapenda sana kufanya hivyo.

IQ; sasa ninyi mmeamua kuvusha wanataka kuacha wenyewe. Sasa pale wanapoondoka kwenye vituo wanahamaje sababu zinakuwa mara nyingi ni nini?

RR; siunajua mazingira, mazingira ya vijijini huku mwingine anakuwa hamna network hana commitment… sasa unakuta mtu amaeajiriwa alikuwa mbeya au dar kuna network na sasa anapofika kule anaona hapafai kwa hiyo unakuta anataka atupiwe ambapo kuna network inashika vizuri anapatikana kwa sababu ukienda sehem nyingine unakuta hapatikani…<door closing> kwahiyo inakuwa ngumu mtu akifika anaona mmhh duu anandika barua ya uwamisho atoke pale<kwahiyo ni hali kama ile mtu inaweza ikamfanya akawa vile.

RR; lakini pia lingine nilishakutana na mmoja hapahapa akawa anaomba atoke kijiji alichopo atleast asogee maeneo ya barabarani kutokana na zile baa za mnara hazionyeshi kule kwenye simu sijui mambo ya kishirikina… akawa anaona zinamshinda kwahiyo hilo nalo lipo kwa kilwa.

RR; mfano kama kuna watumishi huko mtagazi wakifika yani kuna ugumu huwafesi wanasema serikali haina ushirikiano ila kunamambo wanayafesi ambayo niya kitamaduni zaidi mfano mtu anaweza akalala akaamka anaumwa mwili mzima sasa akikueleza hivyo first anataka ahame kule asogee mjini kwasababu hiyo tuu halali usiku umeoona eeh anaota sijui mapopo bawa usiku mtu anajiskia ameingiliwa kimwili kwahiyo imani za kishirikina ni moja ya sehemu zinawafanya watumishi waje huku kuomba barua za uamisho.

IQ; kwa hawa wanaotoka nje ya kilwa wanaajiriwa kenye nini kwenye sehemu binafsi au taasisi za umma. Sasaivi kumekuwa kuna kama upepo watu wanatoka kwenye private wanaenda kwenye public…. Aaaam tutaomba umetupatia hapo kwamba kuna kampuni za uhamaji zaidi hivi karibuni... Nlikuwa nataka niombe maana uchunguzi umeshatupatia…. Sasa tuangalie tunaona jamii inajenga sehemu kubwa katika kuhakikisha watumishi wa afya wanaobakia au wanaokuja wanapaswa wakae nao vizuri… sasa labda niulize swali NI kwa kiasi gani jamii serikali ya hapa kilwa wanaweka mikakati ya kuhakikisha kwamba watumishi wapya hawa ambao wanapelekwa kwenye vituo hutokea huko…. Kuna mikakati yoyote mnayoiona yakuweka…?

RR; sina uhakika ni mikakati gani labda tuu niendele huwa ana give permission kama makatibuu<noise>… information nimeskia kwa mtu kwamba kuna sehemu mwanakijiji alikuwa anasuggest kuacha ujenzi wa nyumba ya kuishi ya mtumishi wa afya kwasasa halina uhakika sana kwasababu hatujaliona kwa macho ni kama uvumishi….

IQ; ivi katika jamii yetu wanaufahamu kwamba katika kata yetu tunatakiwa tuweje au kaya inatakiwa iweje?

RR; kwenye jamii kama jamii …. Watumishi wenzetu.

IQ; lakini pia tunajua kuwa… kwenye kamati kwenye kada kuna kamati zilee…. Eeeh kwanza kabla hatujaendelea hizi kamati zipo? Ebuu tujaribu kuangalia uimara wake katikaa kuhakikisha au katika kuweka mikakati ya kuhakikisha hawa wafanyakazi warudi katika vituo wanarudi au wana… kamati zinafanya nini?

RR; …..Kamati kuhakikisha wasihame<eehh eeeh> ….. Kwahiyo unavyowapeleka kule uwaambie kamati kama kamati kuhakikisha nyumba za watumishi zinapatikana na pia kuhakikisha kama watumishi wanamatatizo au malalamiko wanakuwa na vikao vya kutatua, kuhakikisha wanakuwa na vikao hivyo mara kwa mara ilikujua challenges inakuwasiliana na wale ili kuwasaidia.

IQ; mfano kamati hii ya kata huwa wanafanya vikao mara ngapi kwa mwaka au?

RR; vituo vya kamati pale huwa sanasana wanafanya vikao kila baada ya siku tatu.

IQ; ivi kamati moja ya kilwa kwa mfano kamati ya kata inaundwaje?

RR; sisi kama hospitali tunapeleka matangazo kwa viongozi wa kata wao wanabandika matangazo kwamba tunahitaji watu wazima kadhaa kufwata ule muongozo tunao. Kwahiyo ukiwapelekea ule muongozo watauambatanisha na yale matangazo watu wanajitokeza wanaandika barua wakisha andika barua kamati inachaguliwa na uongozi wa hiyo kamati pale ya kata, wakichaguliwa baadae wanakuja wanabandikiwa<a bit of silence>.

IQ; ahaa kwahiyo lazima wapate muongozo kupitia ofisi ya kata na Je hiyo miongozo inatoka wizarani au wilaya na kata husika je utaratibu hapo ukoje?

RR; miongozo tunapewa kumbuka kwamba ni mijadala mirefu….<echo noise>

IQ; aaah kuna kitu nlikuwa nataka tukiongelee pia kwasababu ninaona kamati za kata, kamati za vijiji, kamati zile za vituo. Ni Kwa namna gani kamati zinashirikisha wananchi katika kupanga mipango ya afya? Je ni kijiji ni kituo au kata?

RR; Kwa maeneo ya hapa huwa wanafanya kwa kutumia ile ya … ya kata kwenye mahala hapa na pale huwa watu wanafanya lakini kwa utaratibu unatofautiana na kata zingine…….

IQ; …. Sasa tujifunze changamoto zinazo tokana na hii mikakati mbalimbali ambayo tunaiona katika ngazi za wilaya ngazi za kata? Ni changamoto gani ambazo mnaziona kwenye hii mikakati ya kuhakikisha uwakilishi wa jamii?

RR; mikakati mingi haitekelezeki zaidi kutokana na pesa hazifiki kwa wakati, mfano wilaya ya kilwa kama hauifahamu vizuri unaweza kuta umejitokezea liwale, rufiji kuna baadhi ya vijiji vipo ndani huko mpka uje ukutane na barabara ni kama km 25 kwahiyo ni mbali. Kuna baadhi ya maeneo hayafikiki ukitengeneza mkakati huu kwamba tukawapatie watumishi wetuu hiki na hiki kiwasaidie ili wafanikiwe ni ngumu kwasababu ni ndani mno na ni ngumu. Kama unavyojua pesa ya serikali haitoki hivihivi unakuta mkafanye supervisin mwezi.

IQ; Na kulikuwa na mkakati wa kuchii…. Je kuna changamoto zozote mmepambana nayo ya uhaba katika huu mwaka?

RR; kwa kazi nyingi ambazo huwa tunafanya kama za hapo kwenye nn ndo zinatolewa.. Tunapiga dawa na tiba mfano kama 10 minutes za kwanza tunatoa dawa au ngao au labda mnataka kufanya training ambazo sindano zinaendesha php sasa kutokana na uhaba wa supplier …..sasa kama haipo kila kitu kina stop mpaka itakapo kuja ndo maana …

IQ; kuna mkakati mwingine ulisema wakujengea nyumba watumishi … je kuna changamoto na huo mkakati?

RR; zipo mfano kama hizo nyumba ambazo ni za serikali au wizara mpaka kuzipata anabidi ahalalishwe kwahuyo government officer. Kwenye ule mpango huwa tuna deni la assemble construction la nyumba za watumishi na ile fedha huwa haitoshi, isipokuwa zile nymba ambazo zimejengwa na wafadhili kama Benjamin mkapa na aketin goal<talkings> huwa tunashindwa kwasababu tuu ya fedha <talkings in the back>

IQ; je kuna jambo lolote la ziada ambalo ungependa kulizungumzia kuhusiana na mikakati ya kuhakikisha watumishi wa afya wanapata hivo vitu vyao ambavyo unadhani.

RR; kwa upande wangu navyodhani kama ingekuwa inawezekana ningependa yani wale watumishi ambao wanaenda kwenye sehemu ambazo ni ngumu kwa kufanyia kazi zaidi maswala ya kiimani. Yani ukipata tuu mshahara wawe angalau wanatoa, yani sijui niseme hapo kuna balaa kumnyima fedha sijui fedha ipi hiyoo sijui fungu gani sijui ila angalu anapata ila ni nyingi ukiachana na mshahara maaana kama mtu anatumia 20000tshs kutoka kijiji kimoja hadi kwenda mjini mbali kwa mshahara wenyewe ndo hivii ila kwakuwa nauli ni 20000 kwahiyo ukipata nauli hapohapo inavyowezekana awe amehakikisha maeneo ambayo ni magumu ya kufanyia kazi. Mfano akipata mshahara mfano akae…. Anatoka mwisho wa mwezi anapata mshahra na katikati ya mwezi kuna hela inakuja kwa ajili ya kuhakikisha …

IQ; bado sasa furaha yao ni kupata palepale sehemu ya kukaa ambayo ni karibu, je kuna mkakati wowote ambao unaweza kutoka huku chini kwa mfano tunahisi kwamba utakuwa unakaa ambao unaweza ukatoka huku chini, labda katika ngazi ya jamii ambayo unaweza ukasaidia… wafanyakazi?

RR; mikakati ambayo ni iseme ni kama hiyo ambayo … tumeona… mfano kijiji au halmashauri ya kijiji ikawa imeamua kujenga nyumba za watumishi sembuse watumishi wangeenda wakesema nyumba hazipo za kuishi. Wale wanakijiji wakaamua kujenga nyumba kwenye field moshi wamejenga nyumba za kuishi.

RR2; ujue hapa kilwa unaweza ukategemea halmashauri ya vijiji kwamba hizi ni kwa low income earners kwahiyo unavyosema kwamba usitegemee sana inategema na wakati na situation ya muda huo….<doors slamming people walking> yani na kwamba … siyo nyingi kule<doors n people walking a lot of silence>

IQ: sasa tunaelekea kumaliza sasa ambavyo tushafika sasa. Nini ushauri wenu sasa kama makatibu kwamba nini kifanyike au ili kusudi kusaidia wafanyakazi wa sekta ya afya wasihamehame wakipangiwa huku kufanya kazi na wakishafika huku?

RR; …. Generally watu wakiangalia huku kusini wanaona kidogo yani mazingira ya huku<people talking in the back> kwamba akija kero ya maji au nini kero kero zingine kama hiyo network ya simu na vingine havipo……. kama wanapata motisha. Kama ninapo kaa nina watu kama kadhaa serikali … watu wanafanya kazi usiku na mchana kama maslahi yao yanakuja kwa wakati hayacheleweshwi na ni wengi sana kwanza hamna nyumba.

RR2; yaaah ni hayohayo tuu kwa sabau social income gapes are very big yani Yule jirani na hao watu wanye maeneo hayo hamuwezi kujicompare…sidhani mtu kama ataweza kuomba kuhamia huku … wengine washazoea kwenda club hizo maisha ya vijana unayajua ni tamaa hata kuosha vyombo hajui ashazoea sehem za kula. Pia kuna huduma zingine kamaa za posta mtandao ya simu kama hivyo watsapp zikiwepo huko sidhani kama watu watakataa kubaki kama transportation barabara magari yakifika unakuta mtu akipata tatizo au dharura kwamba anaweza hata kupata usafiri na kufika dar es salaam ambapo ndo central. Basi mimi sioni kama watumishi sidhani kama watakuwa hawapendi kubaki

IQ; katikati ulisema wanapata mishahara yao wanapata mishahara yao wapi wanapelekewa mikopo au?

RR: huduma za kibenki ziko mbali sana na vituo vyetu vya afya na ni lazima atoke kule alipo aje huku wilayani kweli kwa kupata mkopo ni benki tuu wilaya ya kilwa. Kwahiyo kuna watu wanafunga safari Kama mshahara tarehe 25 anahakikisha trh 23 yupo hapa. Ilitokea mwezi uliopita mshahara ulichelewa kufika watu wakakusanyika wakakaa wiki . Una kuta mtu anatoka kule tarehe 22 au 23 amekaa 24,25,26,27,28,29,30 huku alipofikia ni guest house.

IQ; ivi…. Je kulipwa kule na kufwata mshahara na ukilinganisha na ule mshahara alioupata unatija?

RR; nauli yani hiyo yani imekaa vibaya yani mshahara anaoupata ni mdogo, nauli kubwa sasa ile wiki ukae umalize kila kitu ulichonacho pale, wanapeana ATM card tuu unakuta anaenda na kadi hata sita za wenzake walioko hukoo wanampa na password ili kupunguza zile gharama kwamba akifika inabidi alale sijui afanyaje . Kwahiyo wanampa mtu mmoja ambaye atalala huku achukue mshahara wa wenzake arudi, lakini pia kuna wengine ambao wanatoka huko wenywe vituoni na wanapata mishahara yao vizuri.

IQ; ishawahi kutokea changamoto anaomba lift kwa kumpa password akachukua hela akaondoka. Ishawahi kutokea hiyo au bado?

RR; sijawahi sikia maana wanapendana mpaka basi.

IQ; je hiyo changamoto kubwa ya gharama ya kufwata mshahara takwimu mlizoweza kuchukua zilikuwa ngapi na kama mnaongeza naomba mniwekee bado tunataka tufahamu zaidi kuhusianana na …. Namna ambavyo tunaweza tukasaidia kama kwenye vituo?

RR; mambo amabayo mnaweza kusaidia ni kwenda wizara ya afya na kuwaambia huku chini watumishi wanavyoishi haswa upande wa madaktarii, nursing medium ma clinical officer tupate angalau wengi yani tuna kidogo tumesumbuka kupata hawa….. Japo tunaona challenge pia kupata ma MP au DP wengi huwaga kwa majina yalee.. Mfano mnakuta mmeletewa watatu kama mwaka juzi nakumbuka tuliletewa watatu kama siyo wanne lakini unakuta wale wanne anaweza akaja mmoja tu kulee au asije kabisaa hata kwa mafamasia unaweza ukakuta wapo wawili au watatu. Lakini bado naona tunachallenge wana…yani wale wanaochaguliwa hawaji center pale kwahiyo it’s a challenge sana kwamba watu hawafiki katika vituo walivyopangiwa… tunakuwa na madaktari wachache tunafanyaje? Na tunawahitaji wengi wapo dare s salaam… wote ila una kuta mmoja au wawili lakini wote wanakuwa dar

IQ; nikweli unasema sokoinne wanawabania……..?

RR; kweli yani ni kwamba…. Theatre kwasababu yeye ni mgeni yuko tayar akabane…… au …. Hawa watu wa uhakiki wakija wanakuja wenyewe…..

IQ; Ili baadae wafanye yani biashara tuu….kwasababu………huku na private….

RR; ……. Tuseme wilaya nzima

IQ; kuna utaratibu ulikuwa unasema lazima wapatie serikali… walikuwa wana <horn> wamedisaidi kwamba wakati walikkuwa wana wa promote hawa wakisoma……

RR; hiyo wakionyesha mfano walikuwa wakisomesha wakunga na ma CO kwa ajili ya kufanya kazi nyumbani kwao so na kwa imani but siyo mara moja kwamba ile italipa wauguzi

RR2; jamani lakini siyo kwamba hela ipo pamoja na…..< a lot of noise people talking in the back>….

IS; aaaah nashukuru sana… tumemaliza main talk ya wiki ila imebaki calculation…….. kwahiyo Kalikulaga alibakisha mkuu wa wilaya?

RR; ………

IS; ……..

mwisho

HF-1

IQ una mda gani wa kuwepo hapa kwenye hiki kituo cha kazi?

RR nadhani itakua kama mwezi wa sita.

IQ mwezi wa sita, na kipindi chote hiko umekua ndio mkuu wa hapa?

RR ndio…..

IQ wewe kama mkuu wa hiki kituo yapi ni majukumu yako haswa?

RR majukumu yangu ni mengi pamoja na kusimamia kituo, kusimamia watumishi wengine, (ndio) kutoa taarifa, kuna fanya nanii, na ku order madawa, unaona ehee, yani shughuli zote zinazohitajika kufanywa nafanya. (mhhh)

IQ na unajitahidi vipi kuhakisha kua wafanyakazi walioko chini yako hawahami, unaendelea kubaki nao, angalao kuziba gap la uhaba wa watumishi

RR kwa kweli hasa nimejitahidi, nimejitahidi hasa ni nyumba za watumishi. Kuna nyumba za watumishi dispensary ile tuliohama, ilikua ni dispensary ya awali kwa hio inabidi ile sasa, tukafanyie ukarabati, tufunge huko window angalao, alafu kitu kingine baada ya serikali kutangamaa, unajua sasa hivi bado zipo kwenye heka heka za uchaguzi ….. ni kutafuta maji ya uhakika. Maana shida iliopo kubwa huku chini ni maji, na mtu kukaa mahali bila maji inakua ni ngumu sana. Kwa hio ni maji, umeme, barabara zipo. Kwa hivo tukipata nyumba za watumishi zenye vyoo ndani, unaweza ukamuweka.

IQ na hapa kwako una watumishi wangapi?

RQ sasa hivi?

IR ndio

RR tupo watatu.

IP wewe na hao wauguzi wawili?

RR eeeeeh.

IQ na ….. maara ngapi umefanya uchunguzi akinifu kulingana na idadi ya watu wanao kuzunguka na idadi ya wafanyakazi ulionao ili kuomba uongezewe watumishi. Au watumishi uio nao wanakidhi mahitaji?

RR hawawezi wakakidhi, kwasababu kwanza kuna nanii, ….. tuseme tuna huyu muuguzi mmoja, muuguzi mmoja ambae ankaa jingo la OPD na MCH huku, nab ado tuna jengo la maternity, ambalo hatuna mtumishi, yaani hatuna mtumishi kwa kweli wa kwenda kuna nanilii. Yani tulikua tunahitaji watumishi wa kuongozea manesi kwa ajili ya hizi clinic na bado tunahitaji hata clinical, kwa hio mtu mmoja ukiwa umetoka, umetoka.

IP mmmh, kwa maana nyingine hapa anaetoa huduma ya kutibu ni wewe peke yako?

RR ni mimi peke yangu.

IQ Na kwa wastani unahudumia wagonjwa wangapi kwa siku?

RR 16, 20, ishirini na kitu (mlio wa piki piki)

IQ na …. Zipi ni changamoto kubwa sasa hapa, ukiacha hizo maji, nyumba umeme. Zipi ni changamoto nyingine kubwa zinazo wakabali nyinyi kama kama wafanyakazi ?

RR kwa kweli, changamoto zingine ni hizo nanilii, tunazidiwa. Kwa sababu kuna upande wa, tuseme MCH kuna shughuli ngumu sana, kuna shughuli nyingi, kuna upimaji wa hiari MCH, kuna vipimo vya waja wazito, kutoa chanjo, kuna kutoa family planning alafu unakuta ni huyu hyu, kwa hio inabidi mtu mmoja huyu huyu afanye shughuli zote kwa wakati moja ….. unaona, maana kuna kutoa huduma OPD kwa sababu hata huyu mmoja tulie nae sio nesi ni muhudumu, muhudumu hawezi aka … (simu inaita)

IP kwa hio nesi unae mmoja? (simu inaita)

RR

IQ Ni hali ya hatari ….. na kwa uzoefu wako tangu umefika hapa (mtu anatmbea) ni wafanya kazi wangapi wamehama?

RR tangu nimefika, (furniture inasogezwa) sijui kama ni kuhama, yani tuseme (kelele za viti) mimi nilikua nakaa sehemu moja, sehemu mbii huko, nikaenda nikasoma, nilipo rudi sikua nimepangwa kwenye kile kituo cha zamani. Sasa na yule wa hapa alikua ame wapi? Ameenda kusoma, kukawa hamna mtu na kule kwa sababu palikua pameshapatikana mtu (okay) kulikua na RN mmoja, kuna kaka mwingine alitoka sehemu nyingine walimuomba, basi ikabidi niletwe hapa. (okay) na alivyo kuja nae, ikaonekana kuna sehemu nyingine huko tuliko toka sasa ambako wana anzisha zahanati mpya, ikabidi apelekwe huko, kenda kuanzisha. Kwa hio hakurudi tena. Kwa hio huwezi sema, yeye hakuama kwa … isipo kua ninavyo sikia, kuna kijana alisha letwa kabla sijaja umri huu, akaja tu aka angalia mazingira (akahama) akakimbia.

IP na kwa upande wa wafanyakazi wengine, hawa umewakuta hawa wawili walioko hapa?

RR hapana, huyu mmoja nilimkuta, lakini huyu mama nilienda kuomba kituo kingine cha nanilii, kituo cha afya (okay) baada ya kuzidiwa baada ya yule nesi aliekua MCH wetu kustaafu (ahaaa) ikabidi sasa tukamuombe (okay)

IP na katika kipindi hicho tangu umekuja hapa hakuna alie hama mbali na huyo alie staafu?

RR hapana.

IQ asante sana unafikiri ni kwa nini wewe ume endelea kuwepo hapa kwa miaka sita, na wengine wapo, hao wengine uliopewa wapo hapa, hawajataka kuhama?

RR (anacheka) ni uzoefu tu, ni uzoefu nadhani, yani uvumilivu kwa sababu sio kwamba eti ni pazuri sana au ni pa nini, hapana, ni uzoefu. Kwa sababu mimi niisha fanya kazi, kwanza siku anzia huku, nilianza shughuli Dodoma , Dodoma japo nako kulikua na mazingira mgamu sana kuliko huku (ndio). Unatoka zahanati moja mpaka upate nyingine nafikiri vijiji vingai, yani ngumu, kupata barabara ngumu, kupata hiki ngumu, kupata maji ngumu, hata mashine ya kusagia tu unga, hakuna. Kwa hio mtu unavyo kuja huku unaona sasa, hawa ni vijana ndio, wanalalamika lakini ni haki yao walalamike, lakini kwa wakati huo, tuseme kwanza zamani zile kwanza ukakumbana, zamani zile ilikua nao ajira ya kazi, kulikua na mikataba migumu (mhhh) kwa hio kabla ya kule kuhama, ilikua ni ngumu nak kupata ile… utahama mpaka uambiwe ubadilishe kitu sijui ufanye nini, kwa hio ulikua, kwa hioo watu walikua wanatumia aina mbili, uondoke kwenda kusoma au mwinine aachomoke tu apotee hata usijue ame enda kwa heri au kwa haja hii na ile. (mmhhh) ndio

IQ Na wewe kama wewe, unafikiri kwasababu sasa hivi hatuoni tatizo la watu wamehama, lakini kesho linaweza likaokea (kesho litatokea, linatokea) sasa unafikiri nini kifanyike ili watu wakija hapa hawahami?

RR kwa hio kinacho takiwa kufanyika ambacho nimesha nani, tuseme ambacho nimesha anza cha kwanza, hio ya kwanza ilikua ni nani… ni umeme, mtu akiwa amefunga umeme, (sauti ya piki piki) angalao kidogo anaweza akafunga vitu vyake, kuchaji simu yake, kuanalia TvV yake pamoja na matumizi mengine. (ndio) hio tulisha upata. (ndio) kitu kitakacho fuata ni uwezekano wa kupata maji na majengo, ukiwa na majengo, maji (mhhhh) barabara zipo vizuri, mawasiliano magari yapo, tunaona kwamba barabara sio mbaya sana (ndio). Kwa hivyo tukifanya namna hio, na halmashauri sasa kama halmashauri (mhhh) wakati inapo kwa uajiri, hasa wale watu wa kwanza, ile mafao yao ya kujikimu (ndio) wayatoe kwa wakati (okay), eeeeh wayatoe kwa wakti, mtu akiyapata kwa wakati hata ona usumbufu anaweza akaifanyia chochote au akapanga mahali popote, kwa sababu hata kwa wananchi kuna nyumba, sio ndio? (ndio) huko mtaani kuna nyumba watu hawakai (okay), sio lazima akakae hapa, kuna nyumba, lakini ataenda kupanga nini, kwa sababu hana hela ataenda kupanga na nini? Kwa sababu halmashauri inachelewesha sana, mtu anakuja, ana repoti, hapati hela wiki, anamaliza hela yake iliokua mfukoni, inabidi arudi nyumbani, so inakua ni usumbufu.

IQ mhhhh …… na zipi ni changamoto sasa unazo ziona katika hio mikakati ulio itaja?

RR changamoto ninazo ziona sasa, mikakati, tuseme ni jiji. Kwa sababu hivi vitou vyetu vipo chini ya kijiji (ndio) na vijiji ndio watu, sasa wale watakapo kua, tuseme, ni lazima kuibua, kwasababu kama muweze kuimudu wakielekezwa, watakubali kujenga hivi? Waingie tena mfukoni wajenge na nyumba za watumishi (ndio), kwasababu huwezi kusema serikali ita gharamikia, najua serikali inaweza ikajenga, ni kwamba wao waanze kwanza na serikali ifanye. Hilo hatuwezi kufanya bila hio, kama waki elimishwa kwenye mikutano nanii, hasa wanapo kuja kwenye serikali zao, wanaweza kuona kwamba ni kitu cha kufaa, kwasababu wana ambiwa kwamba mtapata huduma ilio nini? Ilio mbovu kwa hio jitahidini tujenge nyumba za watumishi, kama ni pesa ichangwe tuka vute mabomba sehemu yako maji au tuweke matenki ya kutosha, kwasababu , mambo yanaweza kwenda nanii

IQ naa…. Kama unavyo sema wanakijiji wakihamasishwa, unawaonaje wanakijiji, wana utayari wa kujenga nyumba?

RR wanakijiji wakipata uongozi mzuri kwa sababu kitu kinacho leta shida kwenye kijiji, uongozi ukiwa vizuri tu, wananchi wanakubali kwasababu ukimwabia, mchango wake na hakuona kile kitu kikifanyika sehemu yoyote, anakua na swali la kuuliza kwenye kichwa, (ndio) lakini kama uongozi ukiwa mzuri, hamna kitu kinacho mshinda mwananchi, mwananchi akiongozwa vizuri anatoa (mhhh). Lakini sasa kwa sasa hivi, viongozi sasa ndo tupo leo ni mwezi wa ngapi? (mwezi wa saba) mwezi was aba, sijui mwezi wa ngapi wanachagua (mwezi wa kumi na mbili) mwezi wa kui na mbili, bado hata hawaja, sasa hivi wapo kwenye harakati za kampeini (simu inaita) niendelee? (endelea tu) afadhali

IP Kwa hio shida unayo ona kubwa ni uongozi?

RR uongozi, uongozi kwa kweli kwenye vijiji unakua, uongozi unakua ni mzuri kwasababu watu, watu wanakua, yani uongozi unaopatikana kwenye vijiji hua, hua si kwamba, yani ni kwamba wanachagua tu, yani siasa, kwenye siasa unachagua mtu sio kwamba anajua sana, unaweza kuta kiongozi mwingine hana ufahamu wa kweli, lakini anachaguliwa,

IP kwasababu ya tofauti za kisiasa?

RR yeah, kwasababu tu ya tofauti za kisiasa lakini kiongozi huyu unakuta hana maadili ya uongozi. (sauti ya kitu kudondoka) kwa hio unakuta …

IQ mhhh, na wewe umekaa kwenye sehemu tofauti kabla ya hapa (ndio) na umeishi mazingira tofauti pia (ndio), unautazamaje utamaduni wa wakazi wa hapa unakua kivutio kwa wageni wanapokuja kufanya kazi kwamba wataweza kubaki hapa, maana siku zote hatutapata wafanyakazi wanaotoka kutoka kwenye eneo letu

RR ni kweli (mhhh) kwa uzoefu nafikiri kwakweli wananchi wanawapenda wananii, wapo, labda labda, wanawapenda watumishi, labda mtumishi ungejua hapendwi bado

IP Okay, kwa hio huoni kama utamaduni wa hapa ni tatizo?

RR sio tatizo.

IQ na vipi pengine wakija hapa wageni, wakitaka kununua ardhi, wanaweza kuuziwa ardhi na wananchi?

RR wanaweza

IP au ardhi ni shida, mtu akitaka kuuziwa ardhi ana ambiwa, ahaaa haya mashamba yangu ni ya urithi, hayawezi kuuzwa (anacheka)

RR ardhi, ardhi, inategemea mtu alivyo kaa, kila sehemu anapa kaa mtu, unaweza ukapata, kwasababu kuna watu sasahivi wamekuja, na wameshapata ardhi na leseni wameshakata

IQ, kwasababu mantiki ya swali langu ni kwamba, ili mtumishi akae mahali, adumu hapo, sio tu kwa kumapatia nyumba kuapnga, hakuna mtu anehitaji kua mpangaji maisha yake yote (maisha yake yote ndio) kwa hio atahitaji kununua ardhi waweze kujianzisha, pengine ukitengeneza mkakati wa kuhamasisha serikali za mitaa zika washawishi wanapokuja, kua kuna ardhi za bei nafuu, waka nunua zile ardhi, matokeo yake wakapata ugumu wakuondoka kwasababu wata acha rasilimali walio kwisha zianzisha. Sijui unalionaje hilo?

RR ahaa, hio ni ngumu, hio ni ngumu dokta. (anacheka)

IP kwa maana ya kwamba kidogo kuuza ardhi huku ni ngumu?

RR kwanza ardhi yenyewe unaielewa ni inyu, watu wamebanana kama nini, yani kupata ardhi, sio ardhi yani sema ni kiwanja kama kiwanja tu, kama kipisi tu, lakini kusema kwamba unapata ardhi kabisa heka (na kujenga) na kujenga, inapatikana lakini hata bei yenyewe utaiogopa.

IP sasa unatazamaje hilo kwasababu (hilo nalo ni shida pia) kwasababu leo, tunauhaba huu unao uona, lakini population haija ongezeka sana, kesho utakua na ongezeko la watu kubwa(I kweli) mahitaji ya wafanyakazi (yatakua makubwa) yatakua makubwa, nahata mahitaji ya kituo kupanuka yatakua makubwa, sas je tuamini kua ongezeka la watu lia kwend sambamba sawa na sisi wenyewe kuzaa watumishi wa afya ambao watahitaji kufanya kazi ndani ya jamii yetu, au tuta hitaji watumishi kutoka kigoma, lindi na mtwara? Ambao tunataka waje wakae hapa kwetu lakini tuna waambia nyinyi ni wapangaji wa kudumu

RR kwa kweli hio itakua ngumu, yani tuseme, tuseme miji ya rombo inapanuka, (ndio) kuna tarakea, kuna holili, kuna kule njia panda (ndio) mtu kaipata hela yake, anaweza kwenda kupata kule visehemu kule (ndio) akajenga. Lakini inabidi mtu alivyo kuja, labda kaoa (mhhh) au kavipi naweza kupata huko huko kwa wakwe itakua ni kitu cha aina hiyo, lakini ukisema kwamba ardhi inakusanywa ni ngumu sana.

IP okay, hii nayo ni changamoto ia.

RR hii ni changamoto.

IQ ahaaaa, hapa kuna kamati ya afya? (ipo) unaitazamaje nguvu yake ?(nguvu yake) ina msaada kwenye kutatua matatizo yanayo kabili kituo?

RR hapana, nguvu yak ni kidogo kwa sbabu walipo ianzisha kwenye ODC, sio ya kijiji kimoja, unakuta wako, yani kamati imetoka kwenye kila kijiji. Kwa hio ukiwatuma sasa kijiji kama kijiji kwenye ule muundo wao wa serikali za halmashauri maanake (ndio) ile kamti ile haiwekwi, sijui kunakua na kamati ya nini kwenye kijiji, yani wale hawapo, unakuta hawapo. Sasa kama hawapo ukienda kuzungumzia kwenye halmashauri, hio serikali ya inakua nayo ni ndogo (mhhh). Lakini kama wangekua wameingizwa, mjumbe huyo ameingizwa kwenye zile kamati za serikali, mjumbe anaweza ku save, kwasababu ni moja wapo wa dira itakayo zungumziwa kule. Lakini kwamba hii nafasi yetu ni ya kwenda kuzungumza kwenye mikutano ya kawaida ya kijiji ambayo sio ajabu wakati mwingine asipewe jibu (asitoe) asipewe nafasi. Kwahio mnakuta kwamba mnazungumza mnamua kitu, mnatuma labda title kule kwa serikali ya kijiji au title kule kwa afisa mtendaji, wanasema tutaizugumzia, lakini unakuta wai..

IQ Na ingekua vipi sasa nyie kama kituo mnashirikiana na srikali ya kijiji katika kuhakikisha kwamba utoaji enu wa huduma unakwenda sawasawa kwasababu tunfahamu kuna baadhi ya maeneo, mwenyekiti wa kijiji ana kwenda anasimama ana waambia wananchi “pale kituoni dawa zipo za kutosha nendeni mkapate huduma” lakini wakifika kituoni hakuna dawa, yeye ameenda kuzungumza kule kwa wananchi, kwa hio wananchi wakija, ukiwaambia hakuna dawa, wanasema hao wameficha dawa ili wapeleke kwenye maduka yao watuuzie. Hali hii ikoje hapa?

RR hali ninavyo ijua ni kwamba, ukisha pata dawa, dawa yani wamekwisha kutangazia, wamekupigia simu kutoka kule wilayani famasia, wamekwambia dawa zitaingia labda leo, unahusisha mwenyekiti wa kijiji, mwenyekiti wa kitongoji, na kuna mtu mmoja wa kamati yeye karibu kati waajiri wake. Sasa dawa nitafungua wakiziona na wakijiridhisha kabisa kwamba dawa zimeingia (mhhh) basi.

IQ Mbali na hilo la dawa, kuna mengine ambayo mnashirikiana kwa pamoja, kwa mfano unaweza unakuta kwamba, trend ya minyoo sasa hivi imekua kubwa, malaria imekua kubwa, pneumonia imekua kubwa (ndio), au trend ya magonjwa ya kuhara imekua kubwa, ambayo unajua kabisa tatizo, kwamba tatizo halite isha t kwa kuwapa dawa, tatizo litaisha kwa kuondoa mazingira yanayo waletea shida huko, mnafanyaje katika kushirikiana na jamii kukabiliana na haya?

RR ile ukishaona tatizo la namna hio limejitokeza, unaelekea moja kwa moja kwenye zile nanili, kwanza una toa taarifa kwenye zile serikali za vijiji, (mhhh) ka hio kama kutakua na mikutano ya kitngoji mikutano ya nini, unashirikiana na bwana afya, ambae hayupo akini anatoka kwenye kituo kingine (ndio) lazima wapewe taarifa kwamba kuna kitu flani ambacho kimezuka, kama sasa ivi kuna kipindu pinu tena, kwa hio tumeshatoa taarifa kwamba kuna mtu alihara. Pamoja akiwepo karibu, kuna kipindu pindu walishatangaza, na kuna kipindu pindu kule kwa wakimbizi kigoma, (ndio) na watu unajua mtu anaweza kutoka sehemu moja hadi (nyingine) nyingine. Alafu kuna nanii, vitu vingine tena ni kuhusu chanjo, chanjo sisi hapa tupo kijijini, unapo fika kule kwenye ofisi ya nanilii, ulipo kuta ofisi pale kwenye barabara ile, unajua ni kama kilometa kama 3 uingie nchi ya jirani Kenya, (mhhh, okay) na kwasababu hatujui utaratibu wao walivyo wafikia wananchi wakwao, unaweza kukuta labda chanjo iko chini au iko juu (ndio), kwa hio lengo letu sisi ni kuhakikisha kua kila mtoto ana (anapata chanjo) anafikiwa,

IP kwahio, mnawafikia hadi hao walioko huko kwenye nchi jirani?

RR wanapo taka kuja wanakuja, (okay) sio kwamba akija mtoto wa jirani, (utamwambia hapana wewe mkenya) wanakuja, kwasababu hata kuna wengine wa kwetu wanaweza wakaenda huko.

IQ kuna kituo cha afya, au zahanati ipo jirani hapa inayo hudumia watu wa Kenya?

RR hapana, kuna nanii, ambayo wanaweza kwenda watu wa hapa (eheee) huko kote kuna naniii, kuna zahanati, kuna zahanati ya mission (okay)na vituo vya afya sehemu nyingine lakini mbali kidogo, lakini watu wanaenda, alafu wale wale jamii ya Kenya wakiona kwamba ni vipi, wakitaka labda kupata huduma, wanakuja clinic hapa, watu wana wandikia barua, anakubali kwamba ni mkenya, akifika kwenye hosipitali ya wilaya kama huku, inabidi alipie, mwingine unamwandikia anaenda hospitali ya private, lakini wanakua na ule ushirikiano na serikali, ni vile unashirikishana labda, hata semina za utendaji, tunafanya hivyo. (hio nimeipenda zaidi) ehee tunafanya hivyo (mnajenga, ni muhimu kua na mahusiano, hata kukiwa na matatizo) n ahata juzi juzi ni hivi, kuna mfadhili alitoa mashine ya ultra sound (yeah) (sauti ya mlango) ….. ya ultra sound, wilaya moja inaitwa nanii, wilaya ya … (ndio), imepakana na, walipata kama mashine tatu au nne, na sisi tukapata taarifa tukapata hapo mashine (okay) kwa hio lakini watu walifanya field mbali mbali waka mwambia kua unaweza ukafanyia hospitali ya wilaya mambo yakaenda. Kwa hivo ushirikiano huo.

IQ Mimi nakushukuru sana, pengine kama kuna kitu hatujakizungumza, ambacho unaona kua ni mkakati mzuri wa kuhakikisha kua kuna kua na rasilimali watu

RR wakati mwingine Dokta ni nanii, halmashauri nafikiri kweli zina chelewesha vitu vingi sana (ndio), haijali kumpandishia mtu nanii, apate mafao yake miaka yake inapo fika, apate idara nyingine hajui (ndio) unakuta amesimama mahali pamoja na kwa mda mrefu (ndio) hili linaweza likamfanya mtu akate tamaa. Unaweza kuta umeajiriwa katika wilaya moja na wilaya nyingine mkawa mko tofauti (ndio) unona? Ile interaction kwahio nayo ijali, kwasababu utakuta kule wizara kama kule halmashauri ndo kuna makatibu, kuna walimu, ambao wangesimamia ule utumishi wa ugao, unakuta nae amekuja kukaa pale ofisini, wala haangaiki wala hajui, mpaka mtu anaenda labda … huonii kwamba hilo ni tatizo? (okay)

IP Kwamba watu wamekaa, hawahangaiki, hawafikirii kwamba mtu flani mda flani atatakiwa kupanda

RR hawafikirii, mtu hata akipeleka malalamiko yake kwenye barua, haifanyiwi kazi. Yani kuna watu saa nyingine unaweza kuta labda amehamishwa (mhhh) amehamishwa labda kwenda tarafa nyingine, lakini pesa yake haijaletwa. Kuna wengine labda anaenda shule, halmashauri kwa kweli ukiomba kwa njia nzuri ya kwenda kuwa ambia kwamba naenda shule, ina haki ya kukulipia nusu malipo (nusu gharama) nusu gharama, ambayo halmashauri inawezekana isifanye, au ikalipwa hata usilipwe, maana mtu mwingine unakuta madai hata, madai yakishafika miaka mitatu si kwamba hayatoki tena? (mhhh) . kwa hio inawezekana ikawa ni kitu kinachoweza kufanya mtu aka fikiri hhii wilaya hapa hakuna kutu kinacho weza kujiongoza au mtumishi mwingine aka amua kwenda kuji endeleza akanyimwa kwa sababu kuna upungufu, ndio kuna upngufu, lakini hata kama kuna upungufu (haukiuki haki zako) haukiuki haki zake (ndio, mhhh)

I1 basi mimi nakushukuru, pengine labda kama una swali ambalo ungependa kuniuliza.

RQ nikuulize tuu, labda nijue tu, baada ya kufanya utafiti huu utaleta matekelezo mazuri.

IR ehee, kwakua kazi ya tafiti ni kutoa majibu na kushauri, (ndio) hilo naomba niku hakikishie ka tuta shauri (ndio) lakini inapo fika kwenye utekelezaji hapo inabaki kwao sasa (mhhh) kwasababu wanasiasa wao ndo wanamaamuzi. Lakini nina Imani siasa hazitakua hivi zilivyo wakati wote, dunia inabadilika (dunia inabadilika) kwahio tunapokua tuna majibu hata asipoyatumia alioko leo, kesho atakuja mtu atasema hili jamani likoje? Tutasema majibu haya hapa, atafanya. Kwahio kwa kiasi kikubwa mimi naimani kabisa kuna siku tu lazima haya mambo yatabadilika kwasababu

R yatabadilika, kwasababu kuna wakati

HF-2

Kwa kusimamia wafanya kazi ambao ninao hapa kwenye kituo chetu , na kuhakikisha huduma inatolewa kama ipasavyo, na kuboresha huduma kama iwezekanavyo.

IQ na vipi sasa katika kuhakikisha wafanyakazi wako wana morali, ni nini hasa unafanya waweze kua na morali, kwasababu mtu anapo kua hana morali anaweza hata kama hudua zimepangwa vizuri, vifaa vipo, lakini kama hana morali hawezi kufanya kazi vizuri.

RR nadhani ambavyo tunafanya, tunawapa wafanyakazi wetu vtu, waone kua wamekua appreciated, kwanza anafanya kazi kulingana na barua yake ya ajira imemtaka afanye nini, yani anafanya vile vinavyo husika na cadre yake yeye kama yeye. Na pia zinatolewagwa zawadi kwa wale ambao wanafanya vizuri mwisho wa siku tunatoa zawadi, na kituo kama kituo tuna pokeaga wageni na kuwaaga wastaafu wetu, tuna andaa sherehe ya kwetu binafsi ya kituo kuachana maeneo yale, kwa hio watu wanakua na ile mood ya kwamba ngoja ni fanye vizuri ili ya kwamba mwisho wa siku name nitapata.

IQ na hapo kwenye zawadi kwamfano mnawapa zawadi gani?

RR inadepend sana, tunatengeneza ka mchango, ni kamchango tu kadogo ambayo tunachangishana, kwa hio zawadi ita depend na ule mchango wetu ulifika shilingi ngapi.

IP mhh, kwa uzoefu wako, mara nyingi inafika shilingi ngapi?

RR zawadi? (eheee) mara nyingi hamsini… laki haizidi sana hapo (kelele za karatasi)

IQ na vipi kwa wale wafanyakazi ambao wanarepoti tu kwa mara ya kwana, mmesema mnawafanyia sherehe, mnawakaribisha (mhhh) na vipi mnavyo fanya jambo kama hili, jamii inawatu ambao tunawategemea, wanahusika vipi?

RR katika jamii tuna wakaribishaga majirani, kwamba (kelele) tunapo wafanyia sherehe, kwenye vituo vyetu dispensary tunawakaribisha atleast watu wawili, incharge na nesi wake na wana jamii wa karibu wale wanao tuzunguka tunawakaribishaga mmoja mmoja. Kaya zile za karibu (mtu anacheka)

IQ na vipi kuhusu malazi ya hawa watu wanao repoti kwa mara ya kwanza? Kuna nyumba hapa ambazo mnawapa?

RR kwa karume, tuna nyumba pamoja na sasa hivi uhaba umetokea wachache wamekosa nyumba za hapa ndani kwa hio tuna wachache ambao tuna watafutia nyumba za kupanga maeneo ya karibu na vituoni (naa) na kwa wengine wana kaa hapa hapa

IP wengi wanakaa hapa hapa

RR yeah, wengi wanakaa hapa hapa.

IQ na kwa wale ambao wanapanga nyumba za karibu, nani ambae anawalipia kodi?

RR wenyewe

IQ, wanalipa wenyewe, na kwa wale ambao wamepata nyumba hapa ndani?

RR sasa kuna mgawanyiko, kuna sehemu ambako kuna umeme, unatumika huu wa hosipitalini wao hawachangii chochote, (ndio), ila kuna quarters nyingine wao wanachangia gharama ya umeme pekee (yeaah) kwasababu sasa hivi ni luku

IP Okay, kwa hio wale wanaokua hapa ndani wanapata unafuu wa hio pango la nyumba hawalipi, (ndio) okay, na wale ambao wanapata umeme wwa hosipitali hawalipi chochote (ndio)

IQ na hua ni cadre zipi ambazo zina, hizo nyumba zinatolewa kwa mgawanyo wa cadre kwa mfano waganga wasaidizi, matabibu, wauuguzi au ni utaratibu gani unatumika kua nani awe allocated kwenye nyumba gani?

RR kwa karume, (ndio) nyumba alikua anapewa mfanyakazi yoyote ambae alikuja akakuta nyumba (okay) hai depend wewe ni mganga, wewe ni nesi (ehee) labaratoy, tulikua tunawaapa nyumba watu wote hadi bwana afya alikua anapata nyumba hapa ndani. (okay) kwahio ilikua ni nani aliowahi kufika (okay)akakuta nyumba ipo, anapewa bila kuangalia cheo chake

IQ okay, okay. Na kwa sasa hivi unasema kuna wachache wamekosa, unakadiria ni kama watu wangapi wamekosa nyumba?

RR wapo kama wane.

IP kwa hio kati ya wafanyakazi 28 hao wane t undo hawana nyumba?

RR hao ambao wanakaa huku, maana kuna wengine wanafamilia zao wanakaa mbali na kituo (okay) huja na huondoka kuendelea na familia zao.

IQ ahaaa, kwa hio wenye familia zao, wanakaa mbali kabisa na kituo, hawa vipi, hawapati motisha yoyote kwa ajili ya nyumba?

RR hapana.

IQ hapana, na vipi, halmshauri haina utaratibu wowote labda wa kuwapa incentives kidogo kwa ajili ya nyumba?

RR kwa kweli hatuja pata jipya, san asana wanatuambia kama itakua hosipitali ya wilaya, wanampango wa kuongeza nyumba za wafanyakazi. Lakini mpaka sasa hivi hakuna kilicho fanyika.

IQ oka, na vipi, idadi ya wafanyakazi iliopo hapa sasa hivi inatosheleza? Inakizi mahitaji?

RR haitoshelezi kwa kweli

IP kwa vipi?

RR tuna uhaba, tuna uhaba wasababu kwamfano matabibu hadi sasa hivi tuna matabibu kama watatu (simu inaita)

IP okay, kati ya wangapi wanao hitajika?

RR atleast hata wangekua saba au wanapi, ingesaidia kwasababu wagonjwa ni weni tunawahudumia, per day mnaweza kuhudimia hata wagonjwa mia na (okay) aheee, na hata nurses nao bado ni wachache kwasababu tuna wodi tatu, pediatrics, kuna female ambazo tumezitenga tu general, surgical na maternity lakini unakuta hata wanaingia nesi wawili tu

IP kwenye wodi zote tatu?

RR ehee, nab ado OPD nahitajika nesi wa kutoa damu kwa hio bado kuna uhaba

IQ okay, na pengie kwa kumbukumbu zako, kwa muongozo wa mujibu wa wafanyakazi wanaotakiwa, kituo hiki kilikua kinatakiwa kua na wafanyakazi wangapi?

RR hilo sasa swali gumu, hilo lingekua swali la incharge. (waote wanacheka)

IQ sawa, na hua unakaa kutengeneza mahitaji, kwamba tuna uhaba huu, kila mwaka tunatengeneza kiasi kadhaa na mnatengeneza maombi kwenda juu (mhhh). Unakumbuka kwa mwaka huu mmepeleka maombi ya wafanyakazi wa ngapi?

RR kwa mwaka huu sijui kwa kweli, kwasababu mimi mwenyewe nilikua maternity leave, kwahio kikao cha hapa katikati quarter hii kilinipita.

IQ asante sana, na umesema una mwaka mmoja na nusu, huo mwaka mmoja na nusu umeanzia kazi hapa na uko hapa?

RR kwa hapa nina mwaka mmoja na miwezi (mhhh) kama kadhaa, maana nime anza kazi mwaka jana (okay) mwaka juzi mwezi wane (okay) mhhh.

IQ na upi ni uzoefu wako wa watu kuhama kutoka kwenye hiki kituo na kwnda maeneo mengine?

RR kweli tangia nimefika (ndio) kwa kituo chetu (ndio) kwa mtu mwenyewe binafsi kuamua, haijawahi kutokea, otherwise wilaya tu iamue kua tuna muamisha mtu flani kwenda kituo kingine, lakini kwa mtu kuhama tu kwasababu ya nani, hamna.

IQ na katika kipindi hicho ni wangapi wamehamishwa?

RR katika kipindi hiki wamehamishwa watu wawili.

IQ unafahamu waliko pelekwa? (watatu) (mhhh) wamepelekwa wapi?

RR mmoja amepelekwa ngariku, kuna mwingine amepelekwa mashati government na mwingine ameenda mengwe.

IP wote hao ni ndani ya wilaya hii hii ya rombo (mhhh), hakuna alie ondoka kwenda nje ya wilaya (mhhh)

IQ na pengine unafahamu sababu za wao kuhamishwa kupelekwa huko?

RR hapana, mmoja t undo nafahamu, walisema kwamba kuna uhaba pale mengwe; mashati government, aliekua pale ,tabibu hakuwepo alienda masomoni kwa hio kukakwa hamna mtu ndo wakampeleka. Ila hao wengine sijui, zililetwa tu taarifa kwamba flani ahamie sehemu flani.

I1 Hongereni sana kama hapa watu wanakaa, hawaondoki.

RR aasante.

IQ na katika hilo pia, pengine mwaka huu na mwaka jana hawajahama, lakini wanaweza wakahama mwakani au mwaka kesho kutwa, mmejipangaje kuhakikisha hio hali ya watu kuhama hama haijitokezi.

RR kikubwa kwa kweli kwa hapa kwetu, tunajitahidi tu watu wafanye majukumu yao usije ukamzidishia mtu majukumu yalio zidi na zile basic needs zake azipate. Kwa mfano nyumba, tu akisha fika kituoni akaona kuna nyumba ya kituo, anaona at east kuna nguvu kazi, hela zake za kupoteza kwenye kodi ya nyumba kila mwezi inapungua, unaweza uka mretain kwa mda mrefu

IP kwa hio unafikiri mkakati mmoja wapo wa kuretain watu ni kuongeza nyumba?

RR na za wafanya kazi.

IQ na vipi marupu rupu mengine? Kama marupu rupu hayo ya nyumba kwa wale ambao wamekosa nyumba, unafikiri inaweza ikasaidia? (sautii ya pikipiki)

RR kwa kweli marupurupu yanasaidia, kwa mfano kukiwa na nyumba au hizi hela za … (sauti ya pikipiki inaendelea) zikiwa zinatoka kwa mda unao stahili, inasaidia, inawa encourage hata watu kufanya ile kazi yao vizuri.

IQ posho za night hua zinatoka kwa kuchelewa?

RR sometimes hua zinatoka kwa kuchelewa.

IQ unaweza kwa uzoefu wako kukumbuka kipindi kipi ambacho zilichelewa sana ilikua mda gani?

RR ilikaa kama miezi mitano ndo ikatoka,

IP na ilipo kuja kulipwa, ililipwa yote na miezi yote ya nyuma au ililipwa ya mwezi mmoja?

RR sometimes unalipwa miezi yote, sometimes kuna miezi inarukwa, inakua imepotea hio hela.

IP na hamclaim?

RR hata mkiclaim, mnaclaim lakini hamna response inayo onekana.

IQ unafikiri hii, inashusha morali ya watu?

RR of course inashusha, kwasababu night yenyenwe, duty, ni duty ambayo ni ngumu kwa mfanyakazi kwa kweli kwasababu ameacha usingizi ameingia kazini, sasa ukija kumcheleweshea malengo yake, kwa kweli inashusha morali. (mtu anaongea)

IQ na kwa wastani ni shilingi ngapi kwa night moja?

RR kwa night moja tunatofautiana sasa, kwa nesi ni 5,000, kwa doctors ni 10,000

IQ okay, vipi, mnautaratibu wa kuwabadilisha watu idara kwamba ana rotate anatoka idara hii anakwenda idara hii au?

RR eeeh watu wana rotate, ila kuna wale incharge ndo wanabaki permanent kwa mda flani basi badae ndo wanakuja wana rotate na wao, ili kila mmoja awe na uzoefu, ili hata ikitokea kuna pungufu sehemu flani anaweza aka cover hio sehemu kwa kipindi.

IQ na kwa hao wanao rotate mara kwa mra, mna wafanyia rotation baada yam da gani?

RR tunawafanyiaga rotation baada ya miezi mitatu

IP miezi mitatu

RR mmhhhh

IQ na ma incharge wana rotate baada yam da gani?

RR ma incharge ndo wanakaa mda mrefu hivo, ila hawa wengine, hata wiki wiki (okay), wiki hii anaweza akwa general ward, wiki ijao akawa surgical ward wiki ile nyingine akawa maternity. Na ikioka kuna uhaba hata kabla ya rotation yake basi ana cover sehemu yoyote.

IQ okay, na unatazama changamoto gani kwa haraka haraka katika lengo nililo kuuliza pengine la kuhakikisha kua watu hawa hami hami kwa siku zijazo. Ipi inayo weza kuona kama changamoto kubwa inaypo weza kuathiri mkakati huo?

Okay tuendelee

RR mimi navyo ona ya kwanza, wafanyakazi waongezeke, kwa sababu ukiwa na wafanyakazi wa kutosha na kila mmoja akamiliki department yake, watu hawata ona kwamba kuna uzito wa kazi. Ujue kuna mwingine anahama kwasababu anaona kituo hiki, kazi ni nyingi tuko wachache, kitu kingine nyumba za wafanyakazi zikiongezwa na motisha ziki wekwa wekwa hizo.

IQ hapo kwenye motisha, unaziona motisha za aina gani ambazo zinaweza kuwekwa na zikatekelezeka?

RR motisha kwa sisi watu wa afya kwa kweli hatuna vitu vingi labda seminar, kuna semiar zinatokea tokea angalao mtu akienda analipwa kitu kidogo. Kwa hio kama seminar nazo zinakua ni nyingi na za kutosha, wafanyakazi wanaenda wakirudi , anapata hata morali ya kufanya kazi anajua nikienda Napata kitu hiki, kwasabab siku zote wafanyakazi wa afya wanategemea mshahara hasa asilimia kubwa ya maisha yake yuko kazini kwa hio hana kile kingine cha kumuingizia, n ahata akiwa nacho ni kidogo.

IQ mhhhh, na vipi kwa hapa kwenye kituo hiki, wafanyakazi wote wameajiriwa na halmashauri au kuna wafanyakazi wengine wameajiriwa na taasisi nyingine?

RR hapana, asilimia kubwa ni halmashauri

IP asilimia kubwa? Asilimia ndogo ni kina nani?

RR yani kwa mfano walinzi walivyo ajiriwa kwenye mambo ya halmashauri.

IP okay, lakini watuishi wengine wote wa afya ni wa halmashauri?

RR eheeee

IP kwa hio changamoto zao zinafanana?

RR mhhhhh

IQ na kwa kutazama, kwa mtazamo wako wewe nwenyewe unafikiri tutakapo kua tuna changamoto za kuongeza wafanyakazi na kubakisha wafanyakazi hapa ili wasihame, unafikiri ni kundi gani litaku gumu Zaidi? Ni maafisa tabibu au ni wauguzi au ni watu wa maabara?

RP kufanyaje?

IR kundi ambalokadiri siku zinavyo kwenda, kundi ambalo unaona kwamba kulibakisha, kulifanya wasihame (mmhhhh) itakua ni cgangamoto kubwa Zaidi kati ya makundi yaliopo. Maana kuna makundi ya wauguzi, watu wa maabara na matabibu, unafikiri ni kundi gani litakua na changamoto kubwa Zaidi?

RR kwa nurses, kote kuna changamoto kwasababu kuna uhaba, kwa matabibu kuna uhaba kwa manesi kuna uhaba, kwa hio kote kunaweza kukawa na shida hapo. Nurses na matabibu itakua kundi gumu kai ya hizo, maabara hawana shida.

IP hawana shida sana (mhhhh). Kwa mfano umsema kuna uhaba kila mahali, matabibu mko wangapi?

RR kwa asa hivi tuko matabibu watatu

IP kwa hio wewe, incharge na mwingine mmoja?

RR incharge yeye ni AMO.

IP ni AMO, kuna ma AMO wangapi hapa?

RR AMOwako wawili, ila mmoja sasa ni coordinator wa TB/HIV wilaya nzima, kwa hio asilimia kubwa anadili na mambo (ya TB) kwa hio hatumiki, kwa hio tuseme tuko wanne (okay) incharge mwenyewe ndo kama kazi za incharge unazijua sio kwamba mda wote anakua yupo kituoni, mara yuko wilayani mara afanyaje, kwa hio wafanyakazi tabibu wako watatu, yani active.

IP ambao wote ni waganga wasaidizi (mhh, clinical officers) wote ni afisa tabibu, Kiswahili nacho ni tabu.

IQ kwa hio hapa mko watatu, manesi wako wangapi?

RR manesi wako wengi, (wengi) sina namba yao kamili , lakini wapo wengi lakini hawa kizi,

IP wanaweza kuzidi kumi?

RR eheee, wanazidi.

IP kwa hio shida sana ipo kwenye waganga?

RR ndio ….. hii kwa mda huu , kipindi hiko hao wengine walivyo kwepo hao walio hamishwa hamishwa, (ndio) kazi zilikua zinaenda vizuri, ila tangia mwaka umeanza huu ni shida.

I1 Poleni, naifahamu, mkinimabia mko watatu, na mnaona Zaidi ya watu 100 kwa siku.

R1 Ni shida, tena imekua ni shida kweli sasa hivi sana kwasababu wagonjwa wengi wanakimbia dispel. Yani mtu tu anasema anataka kwenda karume kwasababu tu kuna maabara. Kwa hio unakuta tua attend hata wale waliokua wanatakiwa kwenda kutibiwa dispensary. Au wengine wanaruka kituo cha afya tarakea wanakuja karume, kwabababu wameshajijengea ile intake kua hospitali ya wilaya. Sasa mtu wa kijijini yeye anajua ilishakua hospitali ya wilaya

I1 na pengine mnafanya kazi nzuri ndo maana watu wanakuja. Unaapo ona wagonjwa wanakuja Zaidi inawezekana mnafanya kazi nzuri (inawezekana) hio ni complement. Hongereni sana. Mkiona wagonjwa wanakuja inamaana mnafanya kazi.

R1 sasa halmashauri hawajui hilo, hawalioni, wao wanawachukulia nyie kama kituo cha afya vile vile hawajui kwamba mnapata mzigo wa kutibu wengine wa nje

IQ si hua mnapeleka quarterly report?

RR eheee, si wanasifu tu, aise leo mmezalisha wengi, mmeattend wagonjwa wengi lakini hamna nani wanayo fanya yoyote.

IP hakuna motisha yoyote?

RR ehee, kwasababu inatokea kipindi hata dawa zinakata kabla yam da ….. kwasababu idadi ya dawa inayokuja, inakuja kwa ajili ya watu wenu ambao mlikua mmenanii, lakini wanakuja Zaidi ya hapo. Manapewa tu motisha ya kuambiwa hongereni sana , mnafanya kazi nzuri lakini hamna ile kusema tuongeze hiki na hiki. Ile hamna.

IQ na kituo kama kituo kina changamoto zipi, ukiacha uhaba wa wafanya kazi? (mhhhh) kituo kama kituo kina changamoto gani, kwa mfano dawa kuwepo, miundombinu

RR kwa dawa zinakuwep, pamoja na kuna kipindi kuna baadhi ya dawa tunakosa kwa mda (ndio) lakini tunajaribu kutafuta kwenye dispensary na vituo vingine, tuna cover hiko kipindi. Ila kwa dawa asilimia kubwa, tuna dawa kwa kweli. Miundombinu nayo sio mibaya sana, pamoja na kwamba inatakiwa irekebishwe, kwasababu unaona barabara zetu tukiwa na, wheelchair hatuna, nini hatuna, kwahio tukiwa na mgonjwa ambae yuko serious, watu wambebe mwenyewe wampeleke wodini. Kwahio kuna viu vingine ambavyo havipo.

IQ umesema kua mnaelekea kua hozpitali, kwa maana ya wilaya, (eheee) kwa maana hii itachukua nafasi sasa ya ile huruma ambayo sasa ni DDH. Je sasa hivi mna miundo mbinu kama theatre?

RR ehee, wamejenga pamoja na kuirekeisha rekebisha

IP Ahaa, okay. kwa hio sasa hivi haija anza kufanya kazi

RR haija anza kufanya kazi bado.

IP lakini imeshawekewa vifaa?

RR ehee, tulipata msaada wakatuwekea vifaa (mhhh) vifaa vipo wanarekebisha rekebisa.

IP hii ni ya serikali?

RR mhhh

IQ okay. na zipi ni changamoto za ziada unazo ziona ambazo baadae zinaweza zikachangia pengine kugoma, kudorora n ahata wafanyakazi kuhama….. katika mda mfupi ulio kaa hapa, pengine kuna vitu unaviona kama mhhh, hivi hata visipo, viki endelea hivi, hata mimi nipo njiani.

RR kwa kweli sijaona changamoto nyingi Zaidi ya uhaba wa watumishi, na kukosekana kwa dawa, ndo naona ni changamoto.

IQ okay, nakushukuru sana. Na ni vipi jamii inayo wazunguka inawapa motisha au kua kikwazo katika ufanya kazi kwenu katika kuwahudumia.

RR kama unavyo jua, kwenye jamii kuna watu tofauti, kuna mwingine anakua na ile shukrani kwamba ana appreciate tu kidogo ulicho fanya lakini kuna mwingine hata ukifanayaje hata appreciate, kwa hio wapo watu wa aina hio katika jamii . lakini asilimia kubwa wanathamini tunacho kifanya

IQ na pengine viongozi wa siasa, hawaingilia katika utekelezaji wa majukumu yenu? Kwa mfano kuna shemu unaenda unakuta diwani ndio anataka aamue kila kitu hata visivyo vya utalamu wake.

RR kwa hapa, hawaingilii sana, sema ila kipindi cha siasa kidogo wanasumbua sumbua wakati wanapita huko, mwingine aseme serikalini hamna dawa, karume hamna dawa, karume tukifika tunalipishwa kila kitu, kwa hio kuna mgangano inatokea, lakini kutokea mgongano wa kazi, hapana. Kwasababu tuna ile kamati ya afya, tatizo likitokea tuna washirikisha wao, inakua rahisi kuongea na wana jamii yao.

IQ na hii kamati ya afya muondo wake ukoje?

RR imeandikwa hapa ….. hii hapa. (okay) ambayo kuna waudumu wa afya , asimlimia kubwa ni wa nje

IP hii ni kamati ya afya ya kituo au ni kamati ya afya ya kata?

RR ya kituo.

IQ okay, kamati hio, inawasaidia kutatua matatizo madogo madogo na iko active inafanya kazi yoyote?

RR yeah, iko active na ina saidia kuatua matatizo.

IQ na vipi kwenye kata, kuna kamati za afya kwenye kata? Kwasababu hio ni ya kituo, je ile ya kata ipo?

RR kwa kweli, kwa kata sijui, siwezi nikajibia hilo, sina uhakika kama ipo au haipo?

IP hujawahi kuisikia?

RR sijawahi kuisikia.

IQ nah ii kamati ya kituo, huo mnakutana mara kwa mara?

RR ehee, wanakua na vikao vyao wanakutanaga,wana andaa vitabu, wakija wanakutana.

I1 pengine nitahitaji kufanya mdahalo na wao.

R1 hamna shida.

IQ na weza kuwapata?

RR nita mwambia tu incharge, wanapatikana. (wanapatikana) nadhani atakua na mawasiliano nao. Kwa hio ata wasiliana nao, utaweza kuwapata, kwasababu ni watu wa (hapa hapa) ehee (okay)

IQ na katika haya nilio kwisha zungumza hapa, na wewe umezungumza, kuna jambo linguine lolote ambalo unafikiri pengine ni muhimu tukalipata kusudi tukaondoka nalo. Ambalo unafikiri hujalisema lakini unafikiri ni muhimu katika kuhakikisha kuna kua na rasilimali watu tosha, wenye morali ya kufanya kazi ambao hawa wazi wazi kuondoka.

RR labda, ni kwa hao waajiriwa wapya. Wanachelewa sana kuingia kwenye payroll. Mtu anaweza akaja kazini, amerepoti, hajaingia kwenye payroll. Kwa hio hana kitu chochote hata cha kujikimu, ile hela ya kujikimu haijatoka kwa hio inamuwia ngumu yeye kuingia kazini. Anatoka alipo toka, makazi mapya hana chochote cha kumsaidia maisha kwahio mi nadhani pangetokea utaratibu kua watu wainge kwenye payroll mapema, na hizo hela za kujikumu zinatoka mapema ingesaidia.

IQ kwa uzoefu wako, wakati mwingine watu wanaingia kwenye payroll baada ya mda gani?

RR ahaa, wengine wanachukua hata miezi miwili mitatu, wakucheleweshwa wengine, wengingine wanawaishwa

IQ inapo tokea hivyo, kituo kama kituo hua mnafanyaje kuhakikisha kwamba huyu mtu ambae amesha fika hapa anaweza kuendelea kuishi na kutoa huduma?

RR kwa wale ambao wanakuja, maana mwingine akifika kule halmshauri akikuta bado hajaingia kwenye payroll, haji kabisa kituoni, anaenda kukaa nyumbani mpaka ahakikishe ameshaingia kwenye payroll ndo anakuja. Lakini kwa wale ambao wametoka mbali, lets say labda mtu ametoka mwanza au ametoka Mbeya hadi Kilimanjaro, hana tena fee ya kumrudisha nyumbani. Wakija kituoni, tuna 20% zinazotokana na zile nani za CHF kwa hio kama kituo tunakua tumeshapata hio 20% kuna asilimia flani tunampa na pia ule ubinadamu, sisi wafanyakazi wengine ambao tunapokea mshahara kila mwezi, kila tu anajitoleea, kuna mwingine atasema mimi kwa vile mkulima nitampa unga, nitampa maharage, wengine tunatoa hela. Basi for the time being tunakaa nae mpaka atakapo ingia kwenye payroll.

IQ unalitazamaje hio hali yaw engine, kuleta unga, mchele, unaiona pengine ingekua inafanyika katika mfumo endelevu kwa wafanyakazi kutokana na jamii zinazo wazunguka. Unauona kama ungekua mpango mzuri wa kuwapa motisha wafanya kazi?

RR sasa hio ni ngumu sana ujue, kila mtu anamoyo wake, kuna mwingine atatoa atatoa, ikifika kipindi atasema , ahaaa kituo kimezidi kua na michango (mhhh), unaona eheee, kwa hio sio motisha nzuri kwa sababu itakuja kuleta malalamiko badae. Lakini kama kungekua na fungu linatoka mtu anakua naendlea lakini anakua hadepend kwa mtu flani inasaidia Zaidi. Yani hii mtu tu natoa kwa vile mmeshaamua, kuna yale maaumuzi, kwa hivo mtu anatoa kwa vile mmeshamua kwa yale maamuzi kua fanya hivi lakini sio wote wanatoa kutoka moyoni.

IQ na hapa mnatumia CHF?

RR CHF, NHIF (zote zipo)

IQ na vipi coverage yake ni kubwa? Watu wengi wana NHIF, CHF hapa?

RR of course wapo, mwamko upo, wengi wanazo.

IP wale ambao hawana wanalipa kutoka mfukoni?

RR ehee, ila asilimia kubwa tuna wa encourage walipie CHF kwasababu CHF ni 10,000 kwa watu sita kwa mwaka mzima. Kwahio ni rahisi mtu akalipa hio 10,000 kuliko akalipa akakatwa katwa kila siku 5,000

IQ na hio 10,000 mwaka mzima kwa watu sita ni kwamba anapata dawa na kila kitu?

RR anapata dawa, anamuona daktari anaenda maabara kwa 10,000 kwa mwaka mzima. Kwa hio iko cheap sana.

IQ kwa kweli ipo cheap. Na pengine kwa wale ambao hawajajiunga, kuna kipindi mnafanya assessment kujua kwamba ni kwa nini?

RR sijawahi kuona assessment inafanyika. Pamoja na kwamba kuna wazee hawajajiunga CHF wao, kuna wachache wanakuja na barua za msamaha, wanapitia kwa afisa mtendaji wake, anathibitishwa basi anakuja kutibiwa free of charge, au kuna yule ambae sio mzee, ila uwezo wake upo chini sana, akiwa na barua ya msamaha, anahudumiwa. Ila asilimia kubwa wana CHF kwa kweli.

I1 mimi nakushukuru sana. (asante na wewe pia) pengine mtafiti msaidizi una swali?

I2 asante, naona mambo yote muhimu, umeyauliza.

I1 pengine unajambo unapenda kutu uliza sisi

RQ sijajua sasa nyie, baada ya kuju avitu vinavyo fanya wafanyakazi wasitulie, waka hama hama, mtakuja na Makati gani sasa baada ya kupata mlolongo sahihi?

I1R kazi yetu baada ya kupata majibu sahihi ni kuishauri serikali (okay) tukishaishauri serikali, kwasababu na sisi tupo ndani ya mfumo huo huo, na nikwambie hata kabla ya kuja huku, tayari wizara kama wizara ya afya na tamisemi wanajua kile tunacho kifanya. Kwa hio na wao wamekaa wanasubiria wasikie tutasema nini. (okay) ile tunavyo wapelekea sasa, mwisho wa siku wanatuuliza sasa mnashauri tufanye nini? Kwa hio mwisho wa siku inawahusisha wadau mbali mbali kuona kama inaweza kutumika wilaya chache za majaribio , kuona kama mipango mipya hii ambayo tutaipendekeza inaweza kufanya kazi, alafu ikiweza kufany akzi ndo inafanyika hio tunaita scaling up, kuhamisha kwenye wilaya nyingine, wilaya nyingine, then mwisho wa siku unaweza kukuta kwamba tumehama kabisa kwenye mfumo tuliokua nao zamani, tumekuja kwenye mfumo mpya. Kwasababu hizi ni tafiti za kisera, kwa hio lazima ufanye majaribio uone, tuna angalia na sehemu nyingine walivyo fanya ambazo zinafanana na zakwetu. Kwa mfano tukiangalia nchi tunazo … tunacho kifanya, kimefanyika Zaidi Asia, kwa hio hatuwezi kuchukua, tukaleeta kwetu kwasababu tuna tamaduni tofauti, zimefanyaka hapo pia ulaya kaskazini ambapo miaka 50 iliopita ilikua na matatizo kama haya tulionayo, kwa hio tunavyo fanya hivi sasa tuna shauri serikali, wao sasa ndo wana kauli ya mwisho, lakini sisi kama watafiti ambao tuna burrow kwenye sekta ya afya, ambao tunajua matatizo yaliopo, basi tunashauri kwamba serikali ikifanya hivi tunafikiri huduma zitakua bora, wafanyakazi wataendeleaa kujituma na mwisho wa siku tutayafika yale ya dira ya maendeleo ya mwaka 2025

RR nakushukuru sana kwa hilo (asante sana) naamini utafiti wenu utatuletea tija katika cadre yetu.

I1 tuombe mungu, na tuna amini hivyo. Maana kazi yetu ni kujibu maswali. Asante sana.

HFGC-1

I1 Kazi yenyewe imekua fupi mnoo, lakini kubana matumizi sivyo? (wanacheka) Lakini huku (hayawezi yakaisha kwa kusikilizana, mambo hayaishi, binadamu tulivyo, mama kuja …) aah, tutaweka sera badae

IQ sasa, sasa ndugu mwalimu, kuna mabo machache niitaka tuyajadili hapa, na nina imani kwamba (sauti ya viti), najua mna mkutano muhimu saa, (watu wanaongea nyuma). Nilitaka kufahamu mnashiriki vipi kama jamii, nyie jamii ya masoko, mnashiriki vipi katika kuhakikisha kua mnakua na wafanyakazi wa afya au wahudumu katika vituo vya afya wa kutosha? Labda tuanze kwa kuangalia mna vituo vingapi vya afya hapa masoko kwenye hii kata?

R1R hapa daktari, tuna kituo kimoja

IP mna kituo kimoja tu? (ndio)

IQ ahaaa, na mna shiriki vipi kama jamii katika kuhakikisha mnakua na wafanyakazi wa kutosha katika hiki kituo? …. Karibu mtu yoyote anaweza akasema, mtu yoyote

R1R naweza kusema, ushiriki haupo

IP kwa nini una ona hivyo? ….. mtu mwingine anaweza akajibu

R1R mimi naona kwamba, labda kwa kuchangia nyie wenzangu, ushirikishwaji haupo katika bodi ya afya (ndio) ya kata (ndio) pamoja na walio chini. Hii inakua vitu ambavyo, kila kitu kwetu sisi wananchi tunakiona kigeni tu (ndio) , na tunaweza tukaenda pale kituoni, lakini unakuta hata wanavyo kushangaa, hivi kweli hiki ndio kituo cha afya na kiko chini ya serikali? (ndio) kwa sababu hata wewe mwenyewe ukiondoka hapa ukaenda pae (ndio) unaweza ukasema kama unamaamuzi, kile kituo kifungwe, hakina sababu kuwepo kile kituo. Kwa sababu jinsi kilivyo, uchakavu wake, yae majengo na hali halisi halisia ya kile kituo cha afya ya kwanza kiko mjini, ambacho hakistahili kabisa.

IP je, wafanyakazi walioko pale wanatosha?

R1R eeh, tatizo la wafanyakazi bado ni tatizo sugu sana katika wilaya hii, sio kata ya masoko tu au kituo cha afya cha masoko tu. Ni tatizo sugu ambalo linatukabili, sielewi mimi serikali taratibu zake zikoje. Lakini ukienda pale, utakao wakuta ni watu ambao tunasema kwamba hawa ndio watu tunaowategemea (ndio) ni watu wawili au watatu, wengine wote ni watu wale wazamani tunasema wakabidhiwa wale, wale wote ndio sasa hivi wamekua manesi wakutuhudumia kituo cha afya, sasa hilo nalo tatizo linalo tukabili katika vituo vyetu vya afya pamoja hata na hospitali zetu za wilaya, utakuta hivyo hivyo, utaendaje wakubwa? Kwasababu sasa hivi hakuna hata kinacho jificha, unaambaiwa kabisa wewe ukimkuta amevaa nguo nyeupe, huyo ndo nesi ambae ameenda chuoni kabisa kusoma, lakini ukimkuta mtu tu ambae kava nguo tofati, unajua kwamba huyu ni mhudumu.

IQ ahaaa, na vipi kuhusu, namna ambavyo jamii, watu wa masoko mnavyo wapokea watumishi wapya, watumishi wapya wameajiriwa, wamekuja hapa, mnawapokeaje?

R1R wanapokelewa, kupokelewa kwa sababu sasa hivi serikali ni kwamba haina nyumba, nyumba zote ni za wananchi (ndio) kwa hio wanvyo fika kwetu wanapotaka nyumba ya kukodi (ndio) au kupanga (ndio), wanapangishwa na watu wanakaa nao vizuri tu, tunakaa nao vizuri tu kama wageni wetu

IQ ahaa, vipi kuhusu kuwaendeleza nyie kama jamii, mmekua na mipango gani au kuna mipango gani ya kuwaendeleza wale watumishi wapya au watumishi wa afya katika hivyo vituo?

RP kuwaendeleza vipi?

IR kuwaendelezaa, kistahamishi. kuna mikaati yoyote katika jamii inayo lenga katika kuwaendeleza

R1R jamii inatakiwa ishirikishwe

R2R tatizo ushirikishwaji, jamii ishirikishwe. Mfumo wa wizara ya afya (ndio) ya kuijenga bodi(ndio) na kuileta bodi (ndio)(sauti ya engine nyuma), hio mifuo ipo, (eeeh) sasa baadhi ya challenges ni, kwa mfano mimi nikiingia kwenye bodi ya afya (ndio) ya wilaya, tukakaa tukafikia wadhifa huu ….. sasa jamii haishirikishwi, sasa kama haishirikishwi, inaona tu kwamba alikuja huyu bwana, ni kituo chake (mhhh). Tatizo lake (mhhh) salamu mpya, sasa huwezi uka vifikia, kwa hio unajua tu kwamba huyu ni mtumishi wa serikali na hivyo ni vitu vyake (mhhh). Sasa kama ingekua wananchi wanashirikishwa, katika kushirikishwa uendeshaji wa, sio wa kila siku kama kuchoma sindano (ndio), lakini hata kwenye kuwapokea, hapo sisi indo tuna haki, nje yaw al watumishi, bwana unaona vipi? Aaaaah, mimi halija kasha…. Kwa sababu hali hii bwana, basi watumishi kwenye viwango mbali mbali ndio hayo, (ndio, diploma) diploma (ndio). Lakini mimi sasa sitawaita madaktari mpaka wale waliovaa nguo za kijani, (eeeeh) wote nawaita madaktari (anakohoa), ili sasa tukienda kisiwani (eheee) kwa mheshimiwa Benard, tumeondolewa mtu ambaye ameandikiwa na daktari (mhhh) sasa unampa hayo yote, mhhh, au we sema mwenyewe, (nasema kama kule kwa mkurugenzi…..) ahaaa, (watu wanongea nyuma)

IQ sawa, na vipi kuhusu kuwafunza, maana waajiriwa wengine, watumishi wengine wanatoka nje ya kilwa, hawajui tamaduni za hapa kilwa, vipi kuhusu mnawapokeaje, jamii inawapokeaje na kuwafunza kuhusu taaduni za kama kilwa? Kuna mikakati yoyote ile ambayo mnayo?

RR unajua swala hilo, linakua ni shida, ukimchukulia mtu wa kigoma (ehee) akihamia kilwa (ndio) mazingira ambayo aikua akiyaishi kule na akija hapa na akiwa anaendeleza maendelezo yale, jinsi sisi tutakavyo muona, hata kama wewe utamwambia kwamba, watu wanaishi hivi (watu wanaongea nyuma), kwa mfao labda mavazi, watu wa pwani wapo katika maisha haya,(ndio) haraka haraka inakua ni shida, lakini kama nakua ni mueewa ni kitu ambacho kinakua ni chepesi , inatakiwa twende hivi, twende hivi, twende hivi, tunakua pamoja. Na unapo fika sehemu wa kwei, kwanza unatakiwa usome mazingira ya pale yakoje, na hapo ndipo hata shughuli zako utaziendesha vizuri (sauti za watu nyuma)

IP lakini nilitaka kuangalia katika jamii, kuna utaratibu wa kuwafundisha, hawa wamekuja wapya, tuwafundishe tuwaelekeze utamaduni wa kwetu, na kama hako ka utaratibu kapo?

RR utaratibu huo haupo

IQ sawa, tuangalie jambo jingine, labda tuangalie (clears throat)

R2R hapo abda kabla hatujatoka (ndio), unajua, fumbo linakuja pale pale mwanachi kuachwa nje, kwasababu huwezi kuambiwa kwamba huyu ni mgeni kaja kwenye kitengo chochote cha afya, au labda kitengo cha nini, cha nini cha nini, unatakiwa ni lazima, halafu hukuja ku ripoti (mhhh), basi kama jamii angalao kujua inchi, naniii,mambo ya jamii (mhhh), jamani tumepata mgeni, (mhhh)kutoka mkoa flani, hapa yeye amekuja kwa shughuli zake hizi na hizi. Sasa pale unajua kwamba huyu mtu hata kama unatka kumshawishi, kumpata ili umrudishe kwenye ile mtiririko wa mila na desturi za kilwa, kwa hio unaweza ukamjua, lakini sasa huwezi ukamjua (ndio). Si unaona huyu bwana, bwana afya (ndio) wa mjumbe huyu, (ehee) lakini sasa tunaona mitahani tu kama hivyo, lakini hatuna utamaduni kama huo (mhhh). Sasa kama sisi wengine tuna onana sehemu mbali mbali kwenye vikao na nini, tunajua kwamba huyu bwana yupo hivi na hivi, sasa huyu mwingine hatuwezi kumtambua, tatizo bado linakuja ushirikishwaji unakua haupo katika wilaya yetu

IQ sawa, labda niulize swala hili, hili tatizo la miaka ya hivi karibuni, kuna shida ya watumishi wa afya haba, au wanapangiwa kuja kufanya kazi huku, hawaji,nyie kwa uzoefu wenu mna lionaje? (watu wanaongea nyuma)

RR mimi nafikiri labda bia kuzunguka sana, unajua taizo letu kubwa ni kwa hizi pembezoni (ndio) (sauti ya simu) bado tuna matatizo kama hayo, sio kilwa tu naamini, hata kigoma, eeeh, tabora, yani mikoa ya pembezoni tu karibu yote bado inamatatizo kama hayo (ndio) umenielewa vizuri? Lakini kikubwa, kama kuna mikoa mingine unaweza kusema labda wanahamasisha watumishi wake wanavyo kuja, lakini hizi taratibu ambazo zinashirikishwa kama halmashauri (ndio) au miji midogo, wanashirikishwa na jamii ili kujijenga kwamba hawa wageni wanapo kuja tuwafanyaje ili kuwapa motisha wasitukimbie. Lakini kituo kama kilwa, kilwa ni watu wanakuja kuchukulia ajira. Huyu mfano jamaa yangu, tuna miaka kweli tunaishi kilwa (eeeh), hatuwezi kupata appointment si chini ya miaka miwii, utasikia kwamba amekuja, amepata ajira hapa, ameenda kusoma hio ndio bye bye tayari tumeagana nae, hili ndio tatizo letu kilwa, wengi watumishi wanakuja hapa kupatia ajira tu. Kwa hio hii ndio tatizo

IP na ni kwa nini unafikiri wanaondoka? Kama inakua ni njia

RR unajua tatizo kubwa linalo patikana, kwanza naweza kusema kwamba wale wakuu wa idara wote (mhhh) si wazaliwa (eheee) ….. si wazaliwa, sasa mtu ambae kwa mfano ametoka musoma, mtu ametoka mwanza (ndio), huyu alietoka musoma ndio bossi, huyu alietoka mwanza ndie appointment ya ajira, anamwambia bwana, kilwa ngumu sana. Kwa hio wewe anza tukupe hii nafasi, ukishapata tayari mimi hapa ninakunyanyua.

IP lakini ni kweli mzee, kilwa hapa ni pagumu kuishi kwa wafanyakazi?

RR hamna, huyu bwana si amekuja (ndio) embu muone bwana kwanza alivyo, amekonda? (wanacheka) (aaaah mimi nimekonda bwana sikuja hivi) (wanacheka) unaona bwana huenda angekua mahali pengine … ukweli unabaki hapo, tatizo letu sis indo hilo (eeeh). Kwanza hatujajiandaa na tatizo kubwa la ushirikishwaji

R2R swala kubwa mnaongeleaa hapo ni motivation kwa kweli, unajua kuna kama wenzetu huko songea, wamefika wilaya wapya, walimu na nini , wamefika kwanza wamepewa nyumba, wamechinjiwa makuku, wamekaribishwa kwa shamra shamra, motivation kama hio anapata wilayani ya kufanya nini, kufanya kazi. Lakini hapa unakuja tu, kwanza una ripoti kwa shida yani, unaripoti alafu kwanza utumishi wenyeww wanakusumbua, hawakupatii hela zako, sijui mambo ya mjini yaani mtu kama sio mtumwingine unakua tayari kwenye akili yako unaona yani hii wilaya bwana ina kitu gani ? unana, kwa hio kuna vitu vingi kwa kweli

IQ hivi tujifunze kidogo, hili tatizo la kuhama hama ni kubwa kiasi gani? Ni kubwa sana, kiasi au liko chini?

RR unajua hili tatizo la kuhama inakuaje, (ndio) hali sio kwamba, kwa mfano mimi hapa sasa hivi ni mtalamu, n anime muita mtaalamu, na ametusaidia kidogo, ameangalia (……………) kwa watu waajiriwa wapya (ndio), akashirikisha akiwa kama yeye mtalamu. Lakini tatizo linakuja, kubwa sana linakuja pale pale hakuna mwenye uchungu wa kilwa. Ana sema mimi hapa nitafanya faidika na nitaondoka nitaenda kwetu au sehemu nyingine. Sijui unanielewa vizuri? (eeeeh) sasa kama hatuna ileee, maana hatuna watu walioweza kusoma, kwasababu hili tatizo kwamba tumesisitizwa kusoma, ni kitu sahihi, kama tungekua tumesoma, sisi watu wa kilwa (eeeh) pamoja tunge kua tunasikia uchungu. Kwasababu gani leo mtu anakuja hapa anajua kuna maslahi yake, allowance zake (eheee) na posho zake na nini na nini kitu ambacho ni kushughuikia ili kumjenga kwamba ili asitokee. Lakini kama mtu ametoka huko anajua humu kwa vyovyote hata akitoka hana hasara nay eye atapata tena nafasi nyingine na tena mtu mwingine atka kuja kuomba ile nafasi, atapata chochote, kwa hio hajali kitu. Yule mtu ananyanyaswa kweli, mtu mwenye hasira zake anaamua kuondoka , tuna walimu wangapi hapa wameondoka? Wameondoka wengi tu

IQ kwa hio kama jamii, mnaweza kusema labda mna mikakati gani ya kuhakikisha kwamba watumishi hawaondoki, kwa sababu ni kazi ngumu, awe ni wa afya au wa nini, lakini tunazungumzia wale wa afya, tunahitaji watumishi wawe wametoka wapi ili wabaki hapa watusaidiee? Ni mikakati gani jamii inayo ya kuhakikisha hawa watu hawa hami hami?

RR unajua, kama sisi kata ya masoko ni kata, lakini maswala yote yanayo patikana, yanapatikana katika hospitali ya wilaya, kwa DMO, (ndio) DMO ndio mwenye mikakati ya kujua ni watu gani ambao wametoka wizarani wameajiriwa katika afya. Lakini sasa hao wenzetu wahamiaji ni hapa hawa, wasimamizi hawa, hawa hata kama watakaa tu, watakwenda wapi? Tunacho zungumzia sisi ni wataalamu (mhhh)

IP mzee pale unasemaje? Kuhusiana na hili?

R2R ……………………………………………………………………………………………………………..15

IP kwa hio kwa hili tunalo sema kwamba tatizo la kuhama, ni kubwa au ni dogo au ni la wastani? Wewe unalionaje?

R2R ………………………………………………………………………………………………..

IQ je nikisema kwamba, wageni ardhi hamuwapi, ardhi hamuwapi wageni mnataka mna plough wenyewe hasa hii … kwa hio watu hawana mahali pa kujenga, nitakua nipo sahihi?

RR hata, haupo sahihi, kwasababu gani, ardhi ni mali ya serikali. Na watu ardhi, mipango yao ni mirefu (ndio) ardhi yao, unanieewa vizuri? Sasa wanawezekana wakaja watu kutoka mwanza, kutoka dar es salaam lakini jamii kama jamii, haijulishwi ….. utakuta tayari tu amesha pimiwa, utaambiwa hapa eneo hili la flani. Sasa hilo lenyewe ni tatizo maanake na lenyewe kama mengine ni tatizo vile vile. Lakini sisi wageni wilaya hii wapo wengi, pwani yote hii hamna hata siku moja unasema hamna mtu ukamkosa, yote imetembelwa na wageni tu, kwa hio ni ile hali kwamba, wageni sisi ni wakarimu sana, na ndio maana hata wewe umekuja leo ghafla tu lakinii umetupata watu wa kuonga na sisi, kwamba ni hiii kai yetu ya kuongea (wanacheka)

IQ Amesema pwani yote kuna wageni pia, kwa hio swala la ardhi sio ishu, ardhi ipo tu. Sawa, labda nizungumzie swala linalo husiana na .…. Ahaaa, .…. Ushirikishwaji kwenye kazi nyingine tofauti. Kwa mfano wafanyakazi wapya wamekuja wa afya hapa, tuna jua kuna shughuli za kijamii za misiba, za ndoa, hua wanashirikishwa? Mna wakaribisha? Watu kama hao au mnawaambia tu hizi shughuli …

RR aaah, tunawakaribisha, tunakuaga nao

R2R hapo mwanzoni watumishi wa serikali walikua watumishi wa serikali, na jamii ilikua kama jamii, (ndio) lakini kila tunavyo kwenda ninavyo wakuta wale watumishi wale wanavyo kaa (mhhh) kwa hio badae wenyewe wakaenda tu waka kaa, haya maisha haya sio maisha mazuri, kwa hio tuhame huku, tuhamie kwenye jamii (ahaaa) kwa hio ni vitu ya kawaida tu. Mtumishi akifiwa, watu wote wanaenda, na huku kwetu huku mjini watu wakifa watu wote wanakuja, tunashirikiana vizuri sasa hivi

IP na kuna jambo moja mmezungumza hapo kwamba sasa hivi uhamaji, rate ya kuhama imepungua (ndio) kwa sababu kwanza halmashauri nahisi imejaribu kwanza kuzuia kidogo maana haina fedha za kumuhamisha, labda mtu ajihamishe mwenyewe. Huu ni mkakati wa kwanza ambao umepunguza ile rate ya watu kuhama hama, kuna mkakati mwingine ambao unaufahamu, pengine mnao ambao unasaidia kupunguza lile wimbi la kuhama

RR unaja hapa tulipo kwepo, (ndio) tulikua katika mikakati ya ajira mpya, sasa hivi tunaingia katika eneo la halmashauri, la watumishi (mhhh). Yani mtumishi Yule ambae aliekusudia kuhama kwa kawaida, (ndio) anahama, na kuna Yule mtumishi ambae anasema kwamba mimi kilwa siitaki mimi hata pesa iwepo,isiwepo, inabaki vile vile kwamba kilwa haitaki. Sasa lile ndio wimbi kubwa tunalo likabili sisi, hawa watumishi wa ndani hawa (ndio) wa kuhama hama kutoka labda hapa masoko kwenda ngijo, yule ni mtumishi wa ndani. Laini wimbi kubwa tunalo sikitika sisi, la wataalamu wanao kuja moja kwa moja wameajiriwa, walimu, madaktari, manesi. Sasa wale sisi ndo tuna kipigo nacho kikubwa sana na ndo maana tunasema kwamba ukifanya research katika vituo vyetu hivi vya afya (ndio) labda kama ni wadogo sasa hivi unaweza kupata kama watatu au wanne (ndio) si ndio, lakini ukija huku kwenye zahanai zetu huku, huwezi ukakuta (mhhh) unakuta hizi nguo za kijani tu, sasa hiili ni hatari

IQ sasa hivi vituo au vituo vya afya hivi vya jamii, wanao kuja pale ambao wanatakiwa wahudumie. Ni kwa kiasi gani jamii ina hakikisha kwamba kule kwenye vituo kuna vifaa, kuna dawa? Au kuna vifaa … kwa ajili sasa ya hao watumishi sasa watumie kwa ajili ya kutoa huduma

RR mimi nafikiri kwamba bodi, kwasababu kituo cha afya kina bodi (mhhh), bodi ile ile ndio ambayo ingepaswa kujua (ndio). Lakini tatizo hio bodi yenyewe kushirikisha wananchi (ndio) angalao wakafanya labda hata miezi sita wakaita kikao, kwamba kwanzia mwezi wa kwanza hadi wa sita, bodi tulipokea kama kituo cha afya cha masoko, mnatakiwa mjue hiki na hiki na hiki. Hiko hakuna, na ile bodi tayarii inageuka sasa inakua iko kule kule (wanacheka). Na mkuu wa kituo na bodi, la kwao hili (moja) moja, wewe mwananchi ukienda pale sasa hivi ni aibu kubwa, wewe twende sasa hivi pale, utatoa shilingi elfu mbili, elfu mbile ile utaandikiwa cheti flani utaambiwa nenda duka flani ukanunue dawa, yani unatajiwa na jina kabisa, kama mwanaume manake umemsimamisha mwanaume (ndio) (wanacheka) au unaambiwa nenda kwa mama nyanda ambae amestaafu juzi tu hapo, kituo kipo hapo na duka hapo pembeni, kwa hio anakuambia tu nenda hapo kwa mama nyanda hapo. Kwa hio sasa hili nalo tatizo kwa jamii. (mhhh) unajua mimi nina bahati mbaya sana ya kukutana na nyinyi, na hata hawa ambao wanakuja kufanya ma research mambo ya nini na nini, nakua na bahati nao sana kupatana nao (ndio) na tunapata nao tabu sana, naamini wanapata shida kwa sababu gani kama vile hapa tunafanya hivi, (eheee) lakini marejesho haupo, nikiangalia hapa tunafanya, ni kama tunatwanga maji kwenye kinu tu, tunatoa mawazo yetu, mna andika andika, tayari mkiondoka, nyie mnatuachia shida zetu zile zile hakuna mrejesho, hili sio tatizo. Nafikiri nyie ni kama karibu nyie watano (ndio), wametoka wale watu wamorogoro sijui wapi kule, sijui kituo cha naniliu kilakala kule wamekuja tukaongea nao, wamekuja sijui wakutoka muhimbili kutoka sijui wapi, wakatoka sijui wapi. Sasa kila siku kazi yetu ni kutoa mawazo tu, lakini mawazo haya ni same, ni mawazo haya haya tunayo jadiliana, hamna jingine, ni haya haya, hivi kama sasa hivi nyie wenyewe navyo ni hoji, ingekua vizuri nyie wenyewe muende tu kwasababu hatutakiwi kuongea, cha muhimbili (ndio) sasa muende kituo cha afya masoko sio mbali, hiko hao mnakiona, muende mkaangalie kile kituo kina stahili kua kituo? (clears throat)

IR aah, tumeshafika pale tumeonana na incharge pale, onesmo , tumezungumza nae na watumishi wa afya pale tumezungumza nao pia …. Jumatatu, jumatatu ya wiki hii. Lakini pia tunaenda pale tena jumanne tunaenda pia hospitali ya kinyonga, lakini hii ni kuwa tizama tu. Kuna point ya msingi sana umezungumza ya mrejesho ….. hilo ni muhimu sana, kwasababu hilo unalosema kua nitakuja tena kesho kutwa utaniuliza tena mrejesho uko wapi? Mrejesho hukunipa kikweli uko wapi? Kwa hio ndo ivyo. Kwa hio hio point ya mrejesho kweli unahaki ya kuuliza. Na mimi napokea tu kwa masikitiko kwamba kama hamna mrejesho, kesho kutwa tukikwambia tunakuhitaji mzee, useme sitaki, kumbe ni mambo mengi mengi umesha changia, naomba niifanyie kazi (sawa)

IQ labda tu nizungumzie habari za mila na desturi , hizi mila na desturi za hapa kilwa, ni rafiki kwa wageni wanao kuja au sio rafiki? Ninasema hivi kwasababu nyie mnafahamu,pengine kumekua na watu ambao wanatoka au wanahama kwasababu wanaona kwamba wameshindwa mila na desturi zikiendelea, sio rafiki

RR sifahamu labda nitafute mkono mwingine.

IR karibu

R2R hilo linakua ni shida, kama nilivyo sema awamu ya kwanza pale kwasababu mzingira yanatofautiana, na tuwaeleze yale mazingira ya dar es salaam

RR Jamani mimi niwakatishe kidogoo tu kabla hamjaendelea, dakika tatu tu (dakika tatu tu tumalize) kimavazi, akija tena kilwa (watu wanaongea nyuma) yani akijua, kwamba unamuelekeza kwamba kilwa kuna mazingira haya (ndio) ni sawa. Laini inakua ni shida kwababu swala la mavazi hata serikali haija ipa kipaumble sana.

IP Nashukuru umezungumza swala la mavazi, mzee pale unazungumziaje hilo?

RR aaah, unajua …. Kujiongeza kwake kwasababu, yani jamii inategemea na wewe mwenyewe jinsi ulivyo, (naam) labda mimi nimetoka lindi, nimeenda kigoma. Najua lindi kuna mazingira yangu nilio nayo, lakini nikifika kigoma, naangalia watu wa kigoma wanaishije (ndio) wanakula nini (mhhh) inahitajika na mimi lazima nibadilike (mhhh). Mimi mwenyewe binafsi bila kuniambia mtu, alafu vile vile na mara nyingi mtu unapata shida kutokana na wewe mwenyewe tu unavyo jiweka (mhhh) swa, kwasababu sisi wwenyewe hapa jinsi tulivyo, tumesha pimana hapa (ndio) kwamba huyu bwana kwakua unaanza kukutana nae, yupo hivi, sawa (ehee) unajua kabisa huyu mtu nikikutana nae, inabidi nimuingie vipi (sawa), nimuanze vipi, nimsalimie vipi, sawa (mhhh) sasa ile inategemea wewe mwenyewe unavyo jiweka (mhhh) hapa hivi mimi hapa nilisha kusoma, kwamba wewe bwana kwa vyovyote vile ni mtu mzuri, hauna tatizo, umenielewa vizuri? (ndio) sasa , wewe unatakiwa unisome mimi, nikoje (eeeh),lakini mimi naweza nikasema kwamba nikakupa siku ya kwanza tunaweza tuka kutana popote tukasalimiana, kama huyu bwana si umemgusa (eeh) ukasema kafanana na mtu (eeeh), lakini kama ungekua ni mtu ambae una wasisi wasi usinge mgusa hyu bwana (ndio) lakini ni mtu ambae uko wazi, na huyu bwana akakwambia kwa kweli mimi nahisi tumefanana, samahani sio mimi peke yagu. Kwa hio kwa jinsi utakavyo ishi ndio mazingira yako ambavyo jamii itakupokea, na mila na desturi utajifundiha kwa wewe mwenyewe bila kufundishwa

IQ sasa mnapendekeza, kwa kumalizia sasa, mnapendekeza mikakati gani ambayo itasaidia kupunguza wimbi la kuhama, wale wanao pangiwa kwenye vituo vya kufanya kazi kilwa, wafanye kazi. Mikakati gani ambayo mnafikiri inaweza kutumika

RR miminaona kwa jamii tunakua hatuna sauti, hatuna sauti. Wenye wauti ni wale watawala wetu wanao tuwakilisha, madiwani (ndio) wao ndo wenye sauti, vikao vyao wanaweza kuanga kwamba sisi watumishi wawe hivi hivi hivi. Lakini sisi kama sisi, inawezekana sisi saa nyingine hata mtumishi tusimtake kwa maovu yake, (mhhh) lakini diwani kama haja hamua, bado tunakua hatuna nguvu sana.

IP wewe pendekeza tu alafu sisi tuta penyeza penyeza tu njia… wewe pendekeza unavyo fikiri

RR yani tatizo linakuja, unajua kitu bwana kama hushiriki toka mwanzoni (mhhh) inakua ni shida sana, eheeee, hio ndo hilo, lakini kama unakua unashirikishwa, toka mwanzoni, basi mchango wako mkubwa utakua unaweza kuutoa kikamilifu (labda, pengine hapo kabla hatujaama) labda nikupe mfano mmoja (ndio) kwenye kituo chetu cha afya (ndio) pale kimejngwa choo kwa ajili ya wagonjwa (mhh) lakini jamii au wananchi wenyewe pale hawakushirikishwa, cha kwanza ilitakiwa jamani kwamba watoe taarifa, kituo chetu tuna kususudia kujenga choo, na mtaalamu wetu sisi kalenga seheu flani kujenga choo (ndio).pale jamii nayo pia wakatoa mchango wao (mhh) lakini matokea yake chuo kimejengwa kwa mgonjwa wa kiwaida yani mgonjwa wan je, mgonjwa alielazwa hawezi kutoka wodini kwenda choo kilipo. Unaona kabisa hapa ushirikishwaji umekua ndo… bwana afya anafahamu , atasema ukweli, kama kwei mgonjwa kalazwa, atoke ndani, aende huko kilipo kua choo. Isipokua mgonjwa wa nje, kama nimekwenda pale, imenishika haja ninaweza kwenda (ndio). Kuna vitu kwa kweli, na wananchi wa kaawaida tumeona lakini hatuwezi kukemea, lakini kama diwani angekua na timu yake ya kimsingi na sisi, angetushirikisha

R2R labda nikusaidie kwanza, nilivyo waza (mhhh) kwanza kuna kushirikishwa na kuhusishwa, sawa eeh, wakati kile choo kinajengwa, madiwani walishirikishwa. Unajua tunaposema kushirikishwa, tuchukulie mfano wa kawaida labda tanesco wametangaza bwana leo myohi tunakata umeme kwa masaa kadhaa (ndio), imetushirikisha, yani imetoa taarifa, lakini kama wangetaka kuwahusisha wangekuja labda kwenye jamii wanaita kama watu tatu wanne, wanawaambia jamanii eeeh, ebu tusaidieni, maana hili tatizo ni kali, sisi bwana tumeona vyema kuwahusisha nyie kwenye mawazo, sasa pale madiwanii walishirikishwa kwamba tunataka tujenge choo, na kwa vyombo vya vyoo kama vile vinavyo itwa, VIP ventilated pit latrine, la kutoka eneo mpaka sehemu flani watu wanaishi inabidi kuwe na umbali na wale watu (eheee) na ndo mpango tulioutumia pale tulipo jenga kile choo (ehee). Haiwezekani ujenge kile choo karibu wakati kuna mawodi ya wazazi pale na hili, kuhakikisha kwamba una epusha ule, ule mwingiliano wa labda hewa au wadudu warukao na hivi vingine (eheee, haya) kwa hio pale walishirikishwa, lakini kuhusishwa, wamehusisha wataalamu, kwamba tunaomba mjenge choo umbali flani flani flani kutoka makazi ya watu (mhhh). Kwa hio sisi indo tumehusishwa lakini madiwani walishirikishwa

IP sijui kama tume elewana hapo?

RR anaendelea Yule mtaalamu pale

R2R aaah, mimi nimeshamaliza, nilikua nataka nimsihi tu kwamba yani kwa mfano kile choo kipo palae, kipo sawa kabisa kutokana na sheria za kiafya zinavyo taka.

RR mimi nafikiri tu hapa, bado wote tunalumbana, (mhhh) na tunachotetea ni kituo cha afya kuwa kwenye mazingira mazuri. Maana tumehamishwa kutoka kwenye mada nyingine iliyo kusudiwa tumerudishwa kwenye mada nyigin. Twendeni taratibu kwanza, na hapo napo pana msingi wake vie vile. Mimi kile kituo ni mwenyeji (mhhh) toka hospitali ile haija punguzwa kua kituo cha afya (ndio) toka jamii ya missionary,(ehee) mule kuna system ya vyoo vya ndani, na tumepiga kelele mpaka kikao cha mwisho cha halmashauri kuu, cha utengenezaji wa ilani, tumepiga kelele mpaka daktari tukafikia hatua ngumu sana, DMO ….. lakini juzi nimeenda shangazi yangu kalazwa pale (ndio) kuna mlango wa kufungua wodi ile ile ya kina mama, clinic iko hivi, wodi ya kina mama ya wazazi iko hivi, ya kina mama wa kawaida iko hivi, (mhhh) kuna mlango, ule mlango lakini ulipaswa kutengenezwa kile kitasa tu ili hawa watoke hapo waende chooni kirahisi, sio mpaka wazunguke (mhhh) sasa hili nalo ni tatizo (viti vinasogezwa) umenielewa vizuri? (ndio) ile wodi ya kina mama ile inahitaji, (mhhh) mbele kuna mlango (mhhh) lakini kule nyuma ndo kuna choo lakini kitengenezwe hiki kitasa ili wagonjwa wafungue na kufunga kwenda kule chooni kutoka kwenye wodi hii, hilo ni tatizo vile vile. Kwa ufupi tunakubaliana na mtaalamu alicho kieleza, tunakubaliana nae ni sahihi, unatakiwa umbali kwa ajili ya wadudu sijui na nini na nini, ndo maneno yao wataalmu nimeyakubali (mhhh) kwasababu mimi nakaa nao sana watalamu, najua sana mazingira yao, lakini ivi mlango huu, kutengeneza kitasa ina gharimu shilingi ngapi? Shilingi 4000 tu kitasa. Sasa hivi Tanzania is a ….. mtaalamu (mhhh) bwana afya, embu tuambie, hili nalo tatizo la shilingi 4000, mji mdogo unashindwa kutoa shilingi 4000 tukatengeneza kile kitasa, wale wakina mama wakawa wanafungua wanenda kujisaidia pale wanarudi?

IP mimi nafikiria amekuelewa, labda turudi kwenye mada yetu ya…

RR haya sasa tunajadili, eheee uhakikishaji wa wafanyakazi (Mikakati), mikakati inayo takiwa sasa hivi, kinacho takiwa mikakati, (ndio) kwenye jamii kama alivyo sema, lakini vile vile na wenzetu baraza la madiwani (ndio) bado hawajakaa attention kujua wajibu wao ni nini kujua kwanini watumishi wanaondoka, ila sis indo tunao ona sababu zake

IP eeh, hizo sababu

RR sababu za msingi, kwanza halmashauri yenyewe haijali wale watu wanaokuja (asalaam aleikum, aha mheshimiwa, maongezi yanaendelea nyuma) kama alivyo zungumzia mwezangu kwenye point yake ya kwanza alivyo kuja, kakueleza tabu aliopata mpaka kuweza kufanikiwa mambo yake (ndio), lakini hakufanikiwa kwa vile ilivyo takiwa (eeeh) inawezekana akapata kiduchu, kiduchu, kiduchu, lakini alicho zingatia ni mimi nimekuja hapa kutafuta ajira au nimepata ajira (mhh) kilwa ndo appointment yangu (mhhh) ngoja nitulie, nitazoea tu, sawa eeeeh (mhhh). Sasa pale kinachotakiwa, wale watu wathaminiwe, ile posho yao wanayo pewa, allowance yao, iwe kamili wanapo fika kilwa ili wajikimu (la kwanza hilo, itolewe kwa wakati) itolewe kwa wakati, lakini vile vile mazingira ya kuishi (kwa mfano) nyumba, kwasababu kama halmashauri inashindwa kuwa na nyumba, basi wafanye mawasiliano na watu wa mjini kuona kwamba nyumba zipo (mhhh) za mtu binafsi, hata za watu binafsi, (mhhh) lakini wao si watashindwa kuja moja kwa moja kwa mwenye nyumba, lakini kuna watu karibu, bwana tutafutie vyumba kama vitatu au vine huko mjini,

R2R hilo swala la nymba ni lamsingi sana, anaongelea swala ambalo mimi mwenyewe lilinisumbua sana, yani anacho ongea huyo vyote mvichukue, ni vya msingi sana

RR tuna wetu wanakuja, tuna kama vijana 6, 7 ajira mpya (ndio) kwa hio tunaomba mtusaidie kutafuta vyumba sawa, (ndio) nitatoa mfano flani hapo, nitahama, samahani nitasimama kidogo lakini huo ni mfano hai, wenzetu jeshi la polisi (ndio0 hivi mapolisi wanvyo kuja, hata kama watakuja 60 wale wote wanatunzwa, ndani ya wiki mbili wiki tatu wana tunzwa, wakipata mmoja, mmoja, mmoja, mmoja mpaka wanamalizika (ndio) sasa wale hawahangaiki, halmashauri haina taratibu hizo. Sasa tunaomba sana ili hayo kama mnaweza kuyapenyeza, basi myapenyeze hata makusudio kwamba halmashauri ishawishiwe hata kujenga hizi nyumba rahisi za national hizi, wangezijenga ili watumishi waweze kutulia wapate mahali pakukaa. Kwasababu leo hapa ndo mjini, alafu mtumishi anapata tabu, sasa umempeleka huko ngalanzi huko nafanyaje? (wanacheka)

IQ sasa mazungumzo yako mengi yalikua yana angalia halmashauri idfanye nin. Kwa hio sasa jamii yenyewe ifanye nini?

RR jamii kama jamii ndo tunarudi pale pale kama awali alivyo zungumza mwenzangu, hatuwezi kujua chochote sisi jamiii, sisi jaii tumekaa tu sisi jamii, sijui unanielewa vizuri, tatizo kubwa lipo kwenye ushirikishwaji, haupoo. Washirkishwe kwenye majukumu wajue wafanye nini.

IP leo hii mmeshirikishwa, mngependekeza kuwe na mikakati gani? Ya kuhakikisha wafanyakazi wa afya wanabaki walipo pangiwa hapa kilwa

RR eeeeeh, unajua mimi nafikiria kwamba, kama mapendekezo sisi hatuna, ni kuahidi tu, na namna kule kuahidi, ni kupata vile vitu vyao vinavyo stahiki kwa wakati …..

R2R basi labda kwa hapo kwa watumishi, unajua licha ya anapo tokea, anaanzia sehemu nyingi (mhhh), anaanzia kwanza, achana na chekechea, darasa la kwanza mpaka la saba, form one mpaka form four, five six alafu chuo, mote mule anaangalia mlolongo mzima, alafu sasa (mhhh) ndo ana anza kazi, amerepoti kilwa, anatakiwa aende labda kituo cha afya labda [mnazi…] (mhhh), anakwenda kule, mazingira akijumlisha taswira, haviendani kabisa. Anakuta labda nyumba …. Ya kule, yai akilini mwake anaona bado, anarepoti kule, anarudi wilayani, anafanya mishe mishe, kule hakumfai tena, anaondoka, kulingana na mazingira. Ingekua halmashauri yetu ingeandaa mazingira, vituo vyote vya afya wangejenga numba hata za familia mbili mbili, kwasababu haiwezekani halmsashauri hio hio inajeng nyumba ya milioni 250 au 300 kukaa mkurugenzi mmoja tu, kiasi ambacho milioni 200-300 hizo zingejengwa nyumba za kawaida hata tano, au hata zaidi ya tano, kwasababu mtu kama mimi hapa, milioni 15 kwa nyumba ya kulala tu ya kawaida (mhhh), yani ninaweza nikajenga, yakulala. Lakini mtu mmoja tu ndo anagharimu nyumba yote hio, watumishi wanaenda kulala nyumba ndogo ndogo, akirudi akirudisha taarifa zake, elimu zake na shida zake alizo zipata anatupa huko anakotaka, lakini ndo imesha punguzia ajira kilwa. Ndo alivyo sema kaka, kwamba kilwa imekua sehemu tu ya kuchukulia ajira na kuondoka

IQ sasa mpango huu wa kujenga nyumba naona unatizamwa serikali, kutoka serikali kuu kuja chini na unaelekea kushindwa, maana miaka mingi tumekua tukifikiria na tukitegemea hivyo, sawa wamejenga hizo, lakini jamii na halmashauri wamejitahidi kupitia mashirika ya maendeleo wamepata marupurupu,wamejenga nyumba. Sasa hivi ujenzi wa nyumba tunawezaje tukaufanya ukaweza kujengwa katika ngazi ya jamii, tukawa na uhakika kwamba wenye jamii, tuna uwezo wa kufanya hiki, tukafanya hiki, watumishi wakaletwa hapa na wakae hapa.

RR Au halmashauri yenyewe (ndio) ingefanya jambo moja, ingewashawishi wafanya biashara kila eneo husika, kila kata kuna mfanya biashara maarufu ambae anauwezo wa kujenga nyumba hata nne (mhhh) wangetumia mbinu hio kuwashawishi ili wafanyakazi wao wakawa, kuwaweka pale. Hakuna kata ambayo hakuna mfanyabishara mkubwa mwenye uwezo, anaesshindwa kujenga nyumba mbili tatu za kukaa mtumishi wa kawaida. Ingekua advantage kubwa yani unamshauri, bwana wewe halmashauri (clears throat) sisi tunahitaji utujengee nyumba hizi kwa manufaa yako mwenyewe, na yeye anahamasika kwasababu kitu kinakua ni cha kwake. Lakini ukitegemea yani serikali ifanye hivyo (eeeh) yani inakua ni shida

R2R okay, now, mimi naona vile vile gawanyo. Unajua mgawanyo nao una tu athiri sana. Kama alicho kiongea hapa, ni sahihi kabisa. Ziejengwa nyumba tatu, nyumba tatu zinagharimu karibu milioni 600 (mhhh) sasa hizo nyumba tatu unajengaje kwa milioni 600? Wakati watumishi wengine wanatafuta mahali pa kulala? Sasa hivi ni vitu ambavyo navyo vinatuathiri. nyumba moja imejengwa milioni 300 ya mkurugenzi alafu nyumba nyingine imejengwa milioni 130, wakati wilaya hii, kuna nyumba nyingine za quarter zimejengwa na zinakaa familia mbili mbili, uongo kaka yangu? (ni kweli)sasa ni mgawanyo wa rasili mali amabyo kwa halmashari yetu ni ndogo mno. Kwa hio mtu sasa hivi anjitengenezea mazingira yake yeye mwenyewe, hizo nyumba za milioni 300 embu niambie mtaalamu, embu niambie kwasababu mimi ile ni fani yangu (nimechukua kiwanja hapa beach hapa) ahaaa, imejengwa pale, nyumba ya mkurugenzi, imevunjwa nyumba, yani imevunjwa nyumba sijui unanielewa vizuri, imevunjwa nyumba (ikajengwa nyingine) ambayo ilikua ina lalaliwa na safi (mhhh) sijui unanielewa vizuri, imejengwa nyumba ambayo ina thamani ya kama milioni 300, nyumba hii, moja. Hii nyumba ilio vunjwa ni ya milioni ngapi? Kwahio inamaana kwamba kila mkurugnzi anaekuja anaringa kwamba sasa mimi hapa nitaata wapi tena kwa mradi gani, mkubwa (mhhh) kwa hio anamlaki. Kwasababu ile nyumba imejengwa na marehemu Cornel, kaja mapunda , alafu wote ni ndugu zangu mimi, kaja mapunda anasema hapana, cornel alikosea plan, ngoja nijenge ya kwangu mimi, kajenga nyumba mbili kwa milioni 260. Sasa ukijumlisha hapa mgawanyo wa hali hii, kama ingekua nyumba hizi utaratibu unapangwa vizuri, watumishi wangekua na sehemu ya kukaa , tukapata watumishi wa kuishi. Katika hio milioni 560, tungepata quarter ngapi? Sasa bado tuna tatizo la ugawanyaji wa rasilimali katika wilaya ya kilwa na halmshauri yetu, lakini bado tunarudi pale pale kama alivyo sema kaka angu, tatizo ni madiwani ambao hawajui wajibu wao,

I1 basi tunashukuru, sisi tumefikia mwisho lakini kama una lolote la ziada ambalo litasaidi kuhakikisha watumishi wa afya wa kilwa wanabaki kilwa

RR eeeh, mimi nafkiri kwa mawazo yangu mimi, ni kubwa sana, ushurukishwaji tu. Wakishirikishwa vizuri, raia na watumishi au wakuu wa idara, mzee aliesema mkuu wa idara ya afya ameletewa mtu, basi yeye agalao ashirikishe jamii, jamani tumeletewa mtu huyu hapa, basi mnaweza mkaenda pale mkasalimiana nae, makajadiliana nae, bwana vipi, umepata nyumba? Mimi nyumba sijapata, usipate tabu,kesho utapata nyumba. Tuitwe sisi wanajamii tutakuja kumtafutia nyumba, inakua ina jenga vile vile. Lakini sasa kama kaka nalo kama kaka yangu alivyo sema hapa, kahangaika hapa siku ile tukamkuta anahangaika, kaja pae ofisini kwetu anahangaika, anasema sijui nitaishije, nyumba ndio amepata nyumba kubwa. Sasa ni vitu ambavyo asingekua mtu wa tabu huyu, kama tunashirikishwa

I1 mimi nawashukuru sana

I1 samahani, nitakukatisha, naomba nichukue maelezo kwaababu itatusaidia kwenda haraka halafu tutasikiliza badae tutaweka kwenye… nahisi kwamba hayawezi yakaenda sambamba na spidi yetu ya kuzungumza. Sasa baada ya kuomba idhini, nitarecodi alafu atahamisha ataweka kwenye daftari yake. Labda kwa kusikia kutoka kwako mwenyewe, aah, wewe ni nani na wadhifa wako?

RR aaaah, mimi naitwa Onesmo Vicent Mbinga, mimi ni mganga mfawadhi, kituo cha afya

IP afadhali umenikumbusha, mganga mfawidhi kwanini inakua tofauti na mganga, na umekua hapo kazini kwa mda gani?

RR hapa nimekua kwa miaaaka, tokea 2011

IQ 2011, umewahi kufanya kazi eneo jingine kama mganga wa kiafya?

RR eeeh, nimewahi kufanya

IQ Kwa hio kwa ujumla, wewe kama, kwa kazi yako ya uganga, una mda gani hapa?

RR tangu nimekua mganga, nina miaka kumi na moja, tuseme miaka kumi na moja, mimi nimemaliza chuo mwaka 1998 (ndio) kwa hio na miaka …..

IQ **Lipi ni jukumu lako katika hospitali/Kituo hiki? (Dodosa: Kuhusiana na kudumisha rsilimali watu.**

RR Huwa tunakuwa na mkutano wa wilaya wa wakuu wa vituo vya afya na dispensary wajibu wetu ni kutoa taarifa juu ya uhitaji wa wafanyakazi katika vituo vyetu kwa kadiri ya utaratibu wa kitaalamu na uwiano wa wagonjwa tunao wahudumia lakini mahitaji hayatekelezwi ipasavyo.

IQ Je, kwa jinsi gani unahakikisha kuwa hospiali yako inawafanyakazi wa kutosha? (Dodosa upembuzi yakinifu kubaini mahitaji, vipaumbele, changamoto za malazi na makazi)

RR Upungufu: Tunapohudhuria vikao au kutembelewa na viongozi wa afya wa wilaya tunaweka bayana uhitaji wa wafanyakazi na upungufu uliopo mfano Mfano Chumo zahanati ilitakiwa kuwa na wahudumu 9 lakini tuko wa 4 sawa na upungufu wa wahudumu kwasilimia 56%

**Vipaumbele:** Katika kituo cha zahanati cha Chumo hakuna mganga, hivyo kama tukisema vipaumbele ni kuhakikisha tunakuwa na kada tofauti mfano Hatuna mganga, mimi msimamizi(dispensansary incharge) ni muuguzi wa ukunga, na mwenzangu mmoja na wengine wawili ni wahudumu wa afya(Health attendants) lakini tunapima maabara, tunasikiliza mgonjwa, na kutoa dawa lakini si wajibu wetu kiutaratibu na kiutaalamu.

**Changamoto za Malazi:** kwa sasa tunanyumba mbili, zinatusaidia tofauti na wakati tunaanzaa tulilazimaka kufikia kwa wenyeji maisha yalikua magumu sana kuchangamana na jamii zenye tamaduni tofauti na usizozizoea, ila kwa kuwa zahanati yetu inahudumia vijiji vine(4) uhitaji unaongezeka tunahitaji wafanyakazi, tunahitaji makazi yaongezwe kwa ajili ya wahudumu pindi watakapofika.

IQ Kwa maneno yako mwenyewe upi ni uzoefu wako juu ya uhamaji wa mara kwa mara wa wafanyakazi wa afya, halmashauri na wadau wengine wa afaya. (Dodosa: Ukubwa wa tatizo,wanahamia wapi, na omba takwimu ya wafanyakazi waliohama hivi karibuni)

**RR.** Mimi nilihamia hapa chumo dispensary mwaka 1990 wakati huo nilimkuta msimamizi wa kituo mmoja ambaye aliondoka kwa kustafu na sio kwa kuhama, Ndipo nilipolazimika kupewa usimaamizi wa kituo (Nina miaka 5 kwenye nafasi hii), wafanyakazi wote walioletwa baadae wamekaa isipokua mmoja tu alihama kutokana na matatizo ya afya alirudi kwao ili kupata uangalizi wa ndugu zake, Hivyo muda mrefu tumefanya kazi watu wawili, hawa wengine wamekuja wana miaka miwili. Na mmoja anasiku 4 kazini.

IQ Ipi ni mikakati ya hospitali yako/zahanati/ kituo cha afya katika kuhakikisha hakuna uhamaji wa mara kwa mara wa wafanyakazi wa afya kutoka katika eneo lako na kwenda maeneo mengine? (Dodosa : Kubadilisha idara mara kwa mara,mfumo wa usimamizi, posho Marupurupu, yasiyo ya kifedha n.k)

RR Hatuna tatizo la kuhahama linalohitaji mikakati badala yake tunahitaji wafanyakazi waje tunauhaba mkubwa wa wataalamu, hatuna mganga, hatuna mtu wa maabara, licha ya kuwa na jitihada za kuweka makazi kama kivutio cha wafanyakazi kuishi. Kwa sasa tuna nyumba mbili.

IQ Nichangamoto zipi huikabili mikakati hii ulioitaja (Namba nne) (Dodosa: ukomo wa bajeti, uwepo wa wafanyakazi wa kada tofauti zenye elimu tofauti, na mahitaji tofauti)

RR“Ukomo wa badgeti: Tunauhaba wa fedha za ujezi wa hizo nyumba, kama nlitangulia kusema tuna nyumba mbili na kwa sasa zimeenea kwa maana akiongeeka tu mtumishi mmoja tunakua na uhaba inabidi akapange mtaani ambako makazi yake hayana ubora kabisa.

Uwepo wa kada tofauti: Hapo tupo wauguzi wa wili(2) na wahudumu wa afya wa (2) sote tunashirikiana kusogeza gurudumu la jukumu la kuwahudumia wateja wetu kulingana na uwezo wetu maana changamoto ni nyingi hakuna hatupati motisha ya kazi licha ya kujitolea sana pengine kuliko wenzetu walio mjini.

IQ Je ipo mikakati tofauti na changamoto tofauti katika kukabiliana na uhamaji, wa wafanyakazi wa afya kutoka eneo lako kwenda maeneo mengine (Dodosa ; Utofauti kati ya wale walioajiriwa na wizara ya afya, halmashauri na wadau wengine wa maendeleo; mikakati hiyo na changamoto zake na namna zinavyotatuliwa n.k)

RR Kama nilivyosema awali hatuna watu walio hama kutokana changamoto za kimazingira hivyo hatuna mikakati ya moja kwa moja.

1Q Kwa maneno yako mwenyewe lipi ni kundi gumu zaidi kukaa katika eneo hili? (Dodosa: Kwa kila kada ya wafanyakazi na sababu zake)

RR Tangia niahamie huku chumo mwaka 1990 kada mbili tu zimewahi kupangiwa kutoa huduma huku wauguzi na wahudumu wa afya ambao wote wamejitolea kwa moyo kukaa kazini, maana aliyewahi kuhama ni mmoja tu kwa ajili ya matatizo ya kiafya binafsi.

IQ Ni kwa namna gani jamii ya eneo hili inakuwa kivutio cha wafanyakazi wa afya kuwepo hapa na ni kwa namna gani jamii hii inakuwa kichocheo cha wao kuondoka? (Dodosa: Uwepo wa kamati za afya na uimara wake).

RR Hamna manufaa au hasara ya moja kwa moja juu ya kivutio cha wanajamii kukaa huku au hasara, tunaishi vizuri na wanajamii ila jamii zetu ni za watu wa vipato duni sana maranyingine baada ya wao kukupa motisha wewe sasa wew mfanyakazi inabidi uwasaidie hata katika mambo yao binafsi.

Jambo moja tu ambalo kutokana na uhaba wa wafanyakazi sisi wafanya kazi hatupumziki, hatuna (weekend) na kutokana na jamii za huku ni wakulima wanapendelea Zaidi kupata matibabu weekend, lakini ukijaribu kuwaelimaisha juu ya hilo viongozi wa kisiasa wanaingili mfano marakadhaa diwani ametujia juu ya kututaka tuwahudumia watu jumapili hata kama sio (emergency case) lakini ndo tumesha zoea sasa kufanya kazi hadi sa moja usiku, na hamna mapumziko.

IQ Je, kuna jambo lolote ambalo ungependa kulizungumzia kuhusiana na mikakati ya kutunza rasilimali watu wa afya katika eneo lako? (tafadhari karibu)

RR Kiukweli serikali iangalie sana sisi tuliokubali kujitolea kuishi vijijini kama huku kuwe basi na hata risk allowance kama motisha na watulipe kweli sio maneno tu.

Kuwepo basi na kupandishwa madaraja kwa maana, mfano mimi nafanya kazi zote kama mkunga, kama clinical officer , kama daktari na saizi karibu nishazoea lakini silipwi na kazi zingine wala sio kazi zangu, ningelipwa ningefanya Zaidi bila shaka, lakini hamna kuzingatiwa kwokwote kimaslahi (Classmates) zangu niliomaliza nao na walipangiwa mijiinikwenye fursa saizi wako mbali san ala ona mimi sina kitu.

**FNT-SZ-F1-F3**

1. **Lipi ni jukumu lako katika hospitali/Kituo hiki? (Dodosa: Kuhusiana na kudumisha rsilimali watu.**

**Majibu:**

Sisi huwa tukitembelewa na waakubwa hapa huwa tunasema tunashida ya watoaji huduma hapa wana tuahidi lakini hawatekelezi mfano hapa tupo wawili tu mimi na mwenzangu mfano, saizi ameenda msibani basi nimebaki peke yangu nitakaa hapa bila kupumzika hadi join na usiku ikitokea dharula nipo nayo.

1. **Je, kwa jinsi gani unahakikisha kuwa hospiali yako inawafanyakazi wa kutosha? (Dodosa upembuzi yakinifu kubaini mahitaji, vipaumbele, changamoto za malazi na makazi)**

**Majibu:**

1. **Upungufu**: kiutaratibu hapa tulipaswa kuwa watumishi kati ya (6-9) lakini tupo wa wili(2) tuna upungufu wa 78% na hapa tunahudumia vitongoji vine(4)
2. **Vipaumbele:** Kuwepo na kada zingine ili tugawane majukumu na kila mtu afanye kazi kulingana na utaalamu wake, mfano sisi sote hapa ni wauguzi na tunafanya kazi zote.
3. **Changamoto za Malazi:** Hapa tunanyumba moja na tunaishi wote wawili kwa hiyo kama kwa kadiri mahitaji ya watumishi yalivvyo na malazi pia yako sambamba.
4. **Kwa maneno yako mwenyewe upi ni uzoefu wako juu ya uhamaji wa mara kwa mara wa wafanyakazi wa afya, halmashauri na wadau wengine wa afaya. (Dodosa: Ukubwa wa tatizo,wanahamia wapi, na omba takwimu ya wafanyakazi waliohama hivi karibuni)**

Tangu nifike hapa kituoni nina uzoefu wa miaka Zaidi ya kumi na nimekuwa msimamizi tangu nilipofika, kunawafanyakazi wawili walioletwa na kuondoka mmoja aliolewa hivyo akahamia aliko mumewe yeye sababu ilikua ni ndoa, na mwingine ni clinical officer yeye alikuja akariport alipoondoka hakuwa kurudi tena yeye sijui hakika nini kilitokea ila nilisikia alienda shule licha ya kuwa sijui nini hasa kilichomrudisha nyuma, ni hiyo shule yake au mazingira hakuyapenda.

1. **Ipi ni mikakati ya hospitali yako/zahanati/ kituo cha afya katika kuhakikisha hakuna uhamaji wa mara kwa mara wa wafanyakazi wa afya kutoka katika eneo lako na kwenda maeneo mengine? (Dodosa : Kubadilisha idara mara kwa mara,mfumo wa usimamizi, posho Marupurupu, yasiyo ya kifedha n.k)**

Hatuna watu waliohama kwa sababu nje ya ndoa ambapo kama kituo hatuna wajibu wa moja kwa moja kulifanyia kazi, na huyo mwingine hakukaa kabisa, labda mazingira hakuyapenda, labda ni kweli shule lakini kiukweli hatuna mikakati kama kituo kuzuia watu kuhama. Kwa sababu hawajawahi kuwepo.

1. **Nichangamoto zipi huikabili mikakati hii ulioitaja (Namba nne) (Dodosa: ukomo wa bajeti, i. Uwepo wa wafanyakazi wa kada tofauti zenye elimu tofauti, na mahitaji tofauti)**

Hatuna mikakati hivyo hatuna changamoto zidi ya mikakati kama kituo changamoto Zilizopo ni za jumla kama wilaya na kama taifa.

1. **Je ipo mikakati tofauti na changamoto tofauti katika kukabiliana na uhamaji, wa wafanyakazi wa afya kutoka eneo lako kwenda maeneo mengine (Dodosa ; Utofauti kati ya wale walioajiriwa na wizara ya afya, halmashauri na wadau wengine wa maendeleo; mikakati hiyo na changamoto zake na namna zinavyotatuliwa n.k)**

Hapana,tangia mim niamie hapa hajawi kuja mafanyakazi wala kuhama tofauti na huyu wa kuolewa, na alieripoti na kuondoka jumla bila ya kurudi tena kuanza kazi hivyo sina mtu alieondoka kwa changamoto kuhamia kituo kingine

1. **Kwa maneno yako mwenyewe lipi ni kundi gumu zaidi kukaa katika eneo hili? (Dodosa: Kwa kila kada ya wafanyakazi na sababu zake)**

Hapa kwangu kwa kweli labda huko nyuma hajawihi kuwepo muhudumu wa kundi jingine Zaidi ya muhudumu wa afya na muuguzi, maana aliyeondoka ni muuguzi, labda huyo clinical officer alieondoka baada tu ya kuriport naye siwezi kumsemea maana hakufanya kazi kabisa.

1. **Ni kwa namna gani jamii ya eneo hili inakuwa kivutio cha wafanyakazi wa afya kuwepo hapa na ni kwa namna gani jamii hii inakuwa kichocheo cha wao kuondoka? (Dodosa: Uwepo wa kamati za afya na uimara wake).**

Jamii haina shida kiukweli tunaishi nayo vizuri tu, hatuja wahi kuwa na vikesi kesi na wanajamii kwa muda wote niliokuwepo labda mambo ya kawaida tu ya kibinadamu ambayo huwezi kuzuia.

1. **Je, kuna jambo lolote ambalo ungependa kulizungumzia kuhusiana na mikakati ya kutunza rasilimali watu wa afya katika eneo lako? (tafadhari karibu)**
2. Hapa tunapokaa hakuna mahitaji ya kila siku hadi upande gari umbari mrefu labda uende Nangurukuru, au Masoko ndo upate mboga, mafuta n.k sasa hii inatutesa kwa mtu mgeni ni rahisi kurudi nyuma kutoka na hili.
3. Kuwepo na nafuu ya masharti ya kujiendeleza kwa sisi tuliokuwa tayari tukasome turudi kukaa vituoni. Mfano mimi nikipelekwa kusoma narudi na ujuzi nakuja kuhudumia kituo kwa ufanisi sasa nimeomba kusoma lakini masharti magumu hadi nimegairi.
4. Maslahi hayafanani na kazi haiwezekani kituo kizima tupo wawili tu lakini maslahi duni, hatupumziki lakina hakuna motisha sasa kwa nyinyi vijana wa kizazi kipya hamuwezi kukubali kusulubwshwa namna hii, kwa hiyo uhaba wa wafanyakazi unaendelee.
5. Maendeleo ya jamii kwa ujumla jamii ziendelezwe zijikwamue mfano sisi hata sehemu ya kununua mboga shida, hadi usafiri mbali shida sana kwa kweli.

**STN-2**

1. **ipi ni jukumu lako katika hospitali/Kituo hiki? (Dodosa: Kuhusiana na kudumisha rsilimali watu.**

**Majibu:** Huwa tunakua na vikao vya wilaya mwezi January kila mwaka vya wakuu/wasimamizi wa hospitai/Vituo vya afya na Zahanati huwa tuna toa taarif za upungufu wa wafanyakazi lakini hamana mafanikiao huwa wanasema kuna maeneo yanashida Zaidi kuliko sisi.

1. **Je, kwa jinsi gani unahakikisha kuwa hospiali yako inawafanyakazi wa kutosha? (Dodosa upembuzi yakinifu kubaini mahitaji, vipaumbele, changamoto za malazi na makazi)**

**Majibu:**

1. **Upungufu**: kiutaratibu hapa tulipaswa kuwa watumishi kati ya (6-9) lakini tupo wa wili(2) tuna upungufu wa 78% .
2. **Changamoto za Malazi:** Hapa tunanyumba moja na tunaishi wote wawili. Tatizo kutokana na uchache wetu hatuna shiftitunafanya kazi muda wote si halali kiukweli.
3. **Kwa maneno yako mwenyewe upi ni uzoefu wako juu ya uhamaji wa mara kwa mara wa wafanyakazi wa afya, halmashauri na wadau wengine wa afaya. (Dodosa: Ukubwa wa tatizo,wanahamia wapi, na omba takwimu ya wafanyakazi waliohama hivi karibuni)**

Mimi nilifika hapa mwaka 1997 nilimkuta mganaga, tukawa wawili yeye akahamishwa na serikali nikabaki peke yangu, akaletwa mtumishi mmoja alika miaka miwili akahama kumfuata mume wake, ndo akaletwa huyu sasa tuko wawili tena. Sisi sote ni wahudumu wa afya. Hivyo sina uzoefu mzuri kwani wote waliotoka ni kuhamishwa na kufuata mume.

1. **Ipi ni mikakati ya hospitali yako/zahanati/ kituo cha afya katika kuhakikisha hakuna uhamaji wa mara kwa mara wa wafanyakazi wa afya kutoka katika eneo lako na kwenda maeneo mengine? (Dodosa : Kubadilisha idara mara kwa mara,mfumo wa usimamizi, posho Marupurupu, yasiyo ya kifedha n.k)**

Kiukweli hatuna mikakati kama kituo maana kama serikali ndo ilimuhamisha mganga sisi tutafanyeje, ukiuliza wanasema kuna maeneo yanashida Zaidi kuliko sisi, na huyu mwingine anasema anamfuata mumewe sasa utazuiaje au utaweka mkakati gani kama kituo na hatujawahi kuwa watumishi wengi Zaidi ya wawili, au tuwe wawili au pekeyangu ndo tunaenda hivyo hivyo tu.

1. **Nichangamoto zipi huikabili mikakati hii ulioitaja (Namba nne) (Dodosa: ukomo wa bajeti, i. Uwepo wa wafanyakazi wa kada tofauti zenye elimu tofauti, na mahitaji tofauti)**

Hatuna mikakati wowote hapa alishawahi kuwepo mganga mmoja t undo aliehamishwa na serikali hakuhama kwa kupenda kwake, hivyo huwezi kumsemea na tangia hapo ni wahudumu wa afya tu tumebaki .

1. **Je ipo mikakati tofauti na changamoto tofauti katika kukabiliana na uhamaji, wa wafanyakazi wa afya kutoka eneo lako kwenda maeneo mengine (Dodosa ; Utofauti kati ya wale walioajiriwa na wizara ya afya, halmashauri na wadau wengine wa maendeleo; mikakati hiyo na changamoto zake na namna zinavyotatuliwa n.k)**

Mikakati ya kuzuia kuhama wafanyakazi wenyewe wako wapi, hawaja wahi kuwepo sasa unazuia kuhama nani? Mimi ndo nipo hapa muda mrefu tangu 1997 labda huyu mgeni lakini hamna mkakati wowote akitaka kuhama akipata ruhusa atahama.

1. **Kwa maneno yako mwenyewe lipi ni kundi gumu zaidi kukaa katika eneo hili? (Dodosa: Kwa kila kada ya wafanyakazi na sababu zake)**

Ni ngumu kusema kwa sababu ile ile, hapa tupo wahudumu wa afya tu, na mganga ndo alihamishwa kwa hiyo sijui kwakweli.

1. **Ni kwa namna gani jamii ya eneo hili inakuwa kivutio cha wafanyakazi wa afya kuwepo hapa na ni kwa namna gani jamii hii inakuwa kichocheo cha wao kuondoka? (Dodosa: Uwepo wa kamati za afya na uimara wake).**

Jamii haina shida kwa maana mimi niko hapa muda mrefu naona tunaelewana tu matatizo ni ya kawaida tu ya kibinadamu.

1. **Je, kuna jambo lolote ambalo ungependa kulizungumzia kuhusiana na mikakati ya kutunza rasilimali watu wa afya katika eneo lako? (tafadhari karibu)**
2. Serikali ituangalie kwenye maslhi tunafanya kazi sana tena na muda wa ziada lakini kwa mwaka wanalipa labada mara moja nayo hawalipi kwa ukmilifu, call allowance haziji kama unavyoomba na zinachukua muda mrefu.
3. Iweke motisha maalumu kwa sisi tulio katika mazingira ya vijijini kwani wenzetu wanaunafuu wa mazingira kuliko sisi.
4. Kama hamna motisha watu watapunguza morali wa kufanya kazi.

LG-1

IQ kwa nafasi yako, unashiriki vipi katika kuhakikisha kwamba kituo cha afya na zahanati ilizopo ndani ya eneo lako, zinakua na rasilimali watu wa sekta ya afya wa kutosha? (sauti ya pikipiki)

RR kwanza pal e tuna ushirikiano mkubwa baina, kwasababu kile kituo kipo chini ya kata (okay), kwa hio kila wanacho kifanya tunashirikiana. Na mganga wa kituo pia ni mualikwa katika vikao vyetu vya ODC, kwahio matatizo yanayo tokea kule kwenye kituo cha afya yanaletwa kwenye ODC na yanajadiliwa. Kwa hio ni kama ni swala labda tuseme la usafi, au kuna sehemu labda ambayo ni ya kurekebisha wanaanchi, tukisha ambiwa sisi, tuna waamba wananchi na wanaenda kufanya kazi pale. Swala jingine ni kuhusu bima ya afya, kwenye swala la bima ya afya tunahamasisha kwenye mikutano ya wananchi ndani ya vijiji. Kwa hio wananchi pia na wenyewe wame elimika

IQMhhh, asante sana. Na pengine hua mnashiriki katika kufanya tathini ya idadi ya wafanya kazi wanao itajika katika maeneo haya?

RR tulilifanya hilo mwezi huu wa tano, wezi wa tano tumefanya. Walikuja watu kutoka Dodoma, wakawa wamtuita, tukapeleka wale wanakijiji wa hili eneo la hapa, wakatupa madodoso, tukajaza, kukawa na… walitutenga, kundi la wanakijiji, kundi la viongozi, kundi la atumishi, wakawa wametoa hayo maswali yenyewe yanahusika kuonyesha idadi ya watumishi pale. Kwa hio wakati yanajibiwa, tulijua pale watumishi wapo wa kiasi gani,. Ndio

IQ na, vipi tamaduni za watu wa hapa, zina kidhi, zinasaidia katika kupokea wageni wanaotoka maeneo mengine ambao sio wa hapa? Au zina athiri, zinachangia watu wanao toka maeneo mengine wanapokuja hapa wajisikie kuondoka kwamba tamaduni hazifanani na za kwao.

RR yani tamaduni za hapa, watu ni wacheshi sana, wanapenda sana binadamu wenzao, bila kujali wametokea eneo gani (sauti ya pikipiki) kwa hio hata wale madaktari wanao kaa pale, mtu akija hawezi kujua ametoka mbali kwasababu hap ahata majirani wakiwa na sherehe wana alikwa vizuri tu na kama hayuko zamu anashiriki na sisi (okay) eheee, iwe kwenye misiba, iwe kwenye sherehe tupo nao kabisa vizuri. Na pale si unajua tena, nyumba za kuwalaza waganga hazitoshi, kwa hio raia nao wamekubali kuwapangishia kwao, kwahio wanaishi huku na inaonyesha kabisa kwamba moja kwa moja tayari kuna mahusiano mazuri.

IQ mhhh, na pengine wanapo kuja wafanyakazi wapya, nyinyi kama serikali, mnachukua jukumu la kuwatambulisha kwa wananchi au inakuaje mpaka baadae wanakuta wameingilina na kufahamika na wananchi?

RR wakati, hilo, bado halijafanyika. Ila lakini wakisha julikana ni wafanyakazi wa pale kituoni basi wananchi wana wakubali wanajua huyu ni daktari, kwanza mwingine kifika pale mgonjwa anapo onwa, akija kumuona tena badae anajua huyu ni mfanyakazi wa pale pale

IQ mhh, unafikiri kuwa tambulisha wanavyo fika inaweza kuongeza thamani na pengine maingiliano na jamii yakawa ni marahisi Zaidi tofauti na wanavyo kuta pale kituoni?

RR ehee, ni tofauti, hasa kwa sasa hivi, baada ya kuja, kuna maswala mengine yamekuja ya kiserikali pia, kwa mfano haya maswala ya TASAF ya kunusuru kaya masikini, yanawzshwa na madaktari. Kwa hio unakuta watumishi wengine wanapangwa kwenye vijiji wanashiriki kwenye ile mikutano na wananchi, kwa hio na yenyewe nimeona hilo swala linaletaga picha nzuri sana. Kwahio wakitambulishwa inaleta maana nzuri Zaidi.

IQ mhhh, na vipi kwa hapa kwenye hii nafisi yako una muda gani ?

RP nani mimi?

IR ndio

RR mimi kwenye hii nafasi, mimi ni mtendaji wa kijiji (ndio) kwenye kata nimekainishwa, hakuna mtendaji wa kata, ni mmoja (ndio) ndio

IP kwa hio una muda gani kwenye

RR huu ukaimishwaji miaka mine

IQ miakaminne, (ndio) na kwenye kijiji?

RR kwenye kijiji ni miaka kumi (anacheka), na ni ndani ya jiji nimewakaimu, ninajua mambo mengi

IQ okay (ndio), na sasa kataika huo mda ambao umekaa unatazamaje kasi na idadi ya wafanyakazi wa afya wanao hama kutoka eneo hili kwenda eneo jingine?

RR kasi sio kubwa (sio kubwa), maana naona wakija wanakaa kwa mda. (okay) ndio. Na kukiwa na tatizo labda mtu ameenda likizo yake labda ya uzazi labda kwenda kujifungua au matatizo, lakini kwa ambao nina waonaga, wakija wanakaa walao miaka miwili mitatu, hawaondokagi haraka (okay) hawezi mtu akaja kuripoti na kuondoka (okay)

IQ na hao wanao ondoka, mmeshawahi kujiuliza sababu za kuondoka kwao ni zipi?

RR hawajawai kuondoka kwa haraka, yaani mtu anaweza akakaa miaka mitatu, kwa hio naona tu ni uamisho wa kawaida, hatujawai kuuliza. (okay)

IQ ….. na ikiwa ni hivyo inamaana ina onyesha kwamba wafanyakazi wanakaa hapa kwa mda mrefu (wanakaa). Unafikiri ni kitu gani cha ziada ambacho mmefanya kinacho wafanya watu wasiondoke wafanye kazi?

RR mazingira ya hapa pia ni mazuri, kwa sababu huduma muhimu zinapatikana, kama maji, umeme, huduma hata ya kiroho, makanisa yapo karibu,misikiti iko karibu (okay), soka liko karibu. Kwa hio zile huduma muhiu, yani mahali ambapo kituo kipo, kiko center nzuri sana kiasi kwamba mtu akifika, huduma zote mtu anazipata

IQ (sauti ya piki piki) na vipi, umesema hudua za kijamii zipo, shule zipo pia?

RR eheee, shule ya msingi ipo kwa ajili ya watoto wao, sekondari ipo, za kata zipo mbili ndani ya hii, hapa kwetu (ndio), shule za mingi zipo Zaidi ya 10. Kwa hio yani kila kitu kipo. Kumi na moja, Zaidi ya kumi na moja.

IQ okay (ndio). Na vipi hao wafanyakazi wanao kuja hapa, wanao toka maeneo mengine, pengine hua wanajaribu kununua ardhi? Na wanapo jaribu pengine hua wanapata ushirikiano wa kuuziwa ardhi kwa ajili ya kuendeleza makazi yao?

RR huku kweu, swala la ardhi ni gumu sana, maeneo yani ni madogo (ndio) kiasi kwamba ukitaka mtu wa kukuuzia, ni kazi ngumu sana. (ndio) mashamba haya ni ya kurithishwa tu tangu enzi za mababu , kwa hio maeneo ni kidogo. (mhhh) ndio

IP kwa hio uwezekano wa mfanyakazi kukaa hapa, kuuziwa ardhi ..

RR atauziwa kipande kidogo. Kama robo (robo eka) ndio, lakini sijawahi, ni mmoja, kwenye hili eneo, hajatokea hata mmoja akawa amenunua, akawa yuko huku moja kwa moja, hapana.

IP okay, kwa maana nyingine wote ni wakazi wa mda?

RR eeeeh, ni wakazi wa mda.

IQ unatazamaje hio kwa siku zijazo?

RR sio nzuri, kwa sababu wengi wanavyo kaa huku, naona wanayapenda haya maeneo, ndo maana hawaondoki. Kwa hio wangeweza kupata eneo huku, naona wangeweza kuishi vizuri kabisa.

IP kwa maana ya kwamba wasipo kua na maeneo, ni hatari kidogo kwenu. Kuna siku, mtu huwezi kukaa tangu kijana mpaka uzee ukawa (umekaa tu eneo moja wala huna mji) umepanga tu.

RR alafu unajua tena mtu ukiwa mbali na kule uliko zaliwa, kufanya maendeleo kule kwako ni shida. Mwisho wa siku ukija kurudishwa kule kwenu ni shida (anacheka) ni kazi ngumu kwa kweli.

IQ unafikir kama serikali mtafanyaje katika pengine kuona, ni kweli unasema ardhi ni ya kurithi ni ya kufanya nini, lakini wakati mwingine tuking’ang’ana tu tukisema ardhi yetu ni ya kurithi baadae ika tu athiri kwenye kupata huduma muhimu, kwa mfano sasa hivi tuna wafanyakazi wengi ambao ni wa kada ya kati na kada ya chin indo wako hapa. Lakini mnafahamu kua naenda kua hospitali ya wilaya. Mtakapo kua hospitali ya wilaya mtahitajikua na wafanyakazi wa kada ya juu, mtahitaji kua na daktari sasa (ndio) sio tena waganga wasaidizi (ndio). Mtakapo kua na daktari, unafikiri kwa mazingira yalioko sasa ambap ni kupangisha na kupewa nyumba zile zilizopo pale (ambazo zinatosha) na chance ndogo ya kupata ardhi a kununua (sauti ya pikipiki) mnafikiri mtaweza kukaa na madakatari ambao hata mjini tu wanahama?

RR (sauti ya pikipiki) hilo ni tatizo, ila nilikua nafikiria labda halmashauri ya wilaya iangalie, kama kuna maeneo ambayo, wanagawa maeneo, walao na wao wapewe kipaumbele. Kwasababu kuna sehemu kama kule holily walikua wanagawa maeneo. Kweli watumishi wangepewa kipaumbele ili na wao wapate maeneo ya kuishi huku. Alafu kwa mfano kituo chetu cha afya hapa, kuna kipindi tulitafuta eneo, niliona tulipata kirahisi, kwa ajili ya ujenzi wa mortuary na kujenga OPD (okay) kama nyie mmeona kuna eneo ambalo lina msingi. Hili eneo, hata mimi nilikua na mchango mkubwa sana wakuitafutia halmashauri, tukalichukua kwa wananchi. Na kuna jirani mwingine pia nay eye yuko tayari kutoa eneo kwa ajili ya kuiuzia serikali, kama kuna kitu kama hicho ulicho sema la kuja daktari. Na alikua mesha tuonyesha hili eneo kabisa. Tukasema basi, wakija mission hapa, tutafanya tathmini, sisi tutawapa. Nani eneo kubwa pia. Kwa hio mimi naamini ikija kutokea, tunaweza tukapata eneo.

I1 na ni muhimu. Nasema hivyo ni muhimu kulitazama kwa mipango. Kwasababu kama mnakwenda kua hospitali ya wilaya, lazima mtapata madaktari ambao wanangazi ya shahada kwenda mbele (mhhh). Ambae atakuja hapa amemaliza chuoni ana miaka 29-30, huyu ni mtu anakwenda kuanza maisha (ni kweli) umri wake mbeleni, tuseme ana miaka 30 ya kufanya kazi, sasa hawezi kuwa mpangaji kwa miaka 30, atahitaji kununua ardhi (ndio). Kama kuakua hakuna miundo mbinu kama hio mtashangaa kila wakati anaekuja, baada yam waka miaka miwii anaondoka, kwa hio haitakua tena hospitali ya wilaya, itakua ina jina tu la hospitali ya wilaya lakini huduma zitabaki kama ziivyo kua kwenye kituo cha afya.

IQ na kwa hawa waliopo, mnafikiri kwamba, ni kwa kiasi gani jamii ambayo mnakaa nayo ina wathamini? Na ina wafanya wajione kwamba ni sehemu ya jamii? ili kusudi waweze kuendelea kubakia

RR inawathamini sana. Kwa ninavyo ona mimi, inawathamini sana. Na kwasababu wakati mwingine unakuta masaa labda ya jioni wamepumzika kwenye lile eneo la kwao hata kama mtu alikua anakunywa tu soda unasikia “karibu dokta” yeye mwenyewe tu pale anakataa kwamba “hapana niko busy”. Lakini kama angekua ana nafasi anaweza akakaa na aka nanii, kwa hio tuna wathamini sana kwa kweli hata kama anamkuta barabarani, vijana hawa wa pikipiki, wakimkuta dokta anapanda kwa miguu, wanasimamisha wana mwambia “dokta twende”. Wanawathamini sana.

IQ asante sana, pengine swali jingine dogo, pamoja na kwamba sasa hivi tatizo la watu kuhama sio kubwa, lakini kesho linaweza likawa kubwa sana, hatujui ya kesho. Una pendekeza ni vitu gani vifanyike ili kuhakikisha kwamba wafanyakazi wanapo letwa hapa, wana bakia, kwa kua eneo hili, idadi ya watu wanaongezeka, na idadi ya watu inapo ongezeka tunahitaji idadi ya wafanyakazi iongezeke, na si kupungua, ili huduma ziendelee kua bora.

RR mimi nafikiria, kwasababu hata pale kuna uhaba, wa nyumba za kulala hao madaktari au wafanyakazi wa pale, wengine wanalala nje ya kituo pale. Basi pale paboreshwe, angalao wapate nyumba, tena nyumba ambazo ni nyumba haswa, sio nyumba, yani wajengewe nyumba za kisasa kama ilivyo hai, angalao mtu apende yale mazingira. Kwa sababu mtu anapewa chumba kimoja, na ana familia, anashindwa hata aiweke wapi. Kwa hio wangejengewa tu nyumba nzuri, kwa kutumia hata lile eneo lilioko pale na hilo ambalo tumeshanunua nay ale ambayo tutaendelea kutafuta yaaendelee kutumika

IQ ukizungumzia kujenga nyumba, uazungumzia gharama. (eeeh gharama) unafikiri ni mikakati gani ya gharama nafuu?

RR kutusaidia ama kuomba. Mhhh hapo ni pagumu.

IQ na unafikiri kwenye ujenzi wa nyumba, tutaweza kutumia utaratibu kama tuliotumia kwenye ujenzi wa shule za sekondari, kwa maana ya kuwa wananchi watachangia. Unauonaje uhalisia wa kutekelezeka mpango kama huo? Wananchi watakua tayari kuchangia ujenzi wa nyumba hizo? Na kama hawata kua tayari, unauonaje sasa ule mzigo wa gharama kwenda kwa serikai kuu na halmashauri ambayo mara nyingi imekua ikijibu kwamba hatuna fedha, hatuna fedha. Mradi huo unaona kama utawezekana?

RR hapana utakua mgumu. Kwasababu wananchi wenyewe ninavyo ona, kua tayari kujenga nyumba kama nyumba kwa michango yao wenyewe kama wenyewe itakua ni kazi ngumu sana. kwasababu hata pale ukianza kuangalia kuna vitu vingine wataanza kusema, hata kazi ya kujitolea, watakuambia, “sisi tunalipia bima ya afya, kwa nini hio bima ya afya, hio hela yetu tunayo itoa isifanye kazi?” hawajui kama ile hela inaenda kwenye madawa. Kwa hio itakua kazi ngumu

IQ sasa unashauri nini kifanyike? Ambacho kitakua ni cha gharama nafua lakini wakati huo huo kitatusaidia sisi kama wananchikuendelea kupata huduma kutoka kwa wataalamu wetu hawa?

RR mimi nafikiria basi hata yale mananii, zile nyumba ambazo zipo, walao ziboreshwe kwasababu walao gharama yake itakua ni kidogo. Zifanyiwe walao ukarabati wa mara kwa mara.

I1 Pengine nikushukuru, lakini nikuambie, kama unajambo lolote la mwisho ambalo unafikiri ni muhimu ukalisema, ambalo litachangia kuhakikisha tunakua na wafanyakazi wa kutosha kweye sekta hii ya afya hapa kwetu, ambalo unafikiri hatuja lizungumza katika mazungumzo haya, ni wakatii wake ukalisema.

RR mimi nilikua nafikiria tu nikwamba….. hawa wafanyakazi wetu wapewe walao motisha kwenye kazi zao, ili waweze kuipenda kazi. Kwasababu ile kazi unaiona kabisa, ile kazi ni wito, kazi ile ni wito. Kwa hio serikali na wao, iwaboreshee walao hayo mazingira na kuawapa walo allowance kidogo kwenye hizi shughuli zao.

IP mmmh, motisha, kama zipi?

RR kama kuna stahiki zao, ambazo wengine wanadai labda walao wapewe kwa wakati, (mmh) na kingine basi sehemu kama hii, hata pale ambapo kuna mtumishi kwa mfano dereva. Dereva wa kituo aajiriwe permanently, asiwe anasumbuliwa sumbuliwa au kama ni mshara wake, maana unajua ile kazi pia, usiku mtu kuhangaika barabarani na wagonjwa na nini, inabidi dereva na awe anapewa sana kipaumbele, akae Zaidi.

IQ asante sana, kwa hio hapo hapo ulipo sema waangaliwe motisha, unatazamaje nafasi ya wanasiasa kwenye eneo hili, na maingiliano yao na watumishi wa afya, wana wapa motisha? Au wanachangia kuwa vunja moyo?

RR wana wapa motisha. Wanasiasa wa hapa (ndio), kwa mimi ninavyo waona (ndio) wana mahusiano mazuri sana na watumishi. Hata daktari akiwa labda na tatizo, akimpigia hata mheshimiwa diwani, faster unaona anaenda. (okay) eeeh. Hawana shida kabisa.

I1 Nashukuru sana. Pengine mtafiti msaidizi una swali la nyingeza?

I2 nashukuru sana, tumeona mambo yote ya msingi, umeuliza na ameyajibu vizuri. Hapo sina swali.

I1 pengine una swali kwetu?

RQ mimi labda swali, nikuwapa, kuna hapo sasa tunapo zungumzia kuhusu uboreshaji wa wale watumishi wa health centre. Mimi naomba tu basi mki … mtusaidie, ili na sisi kwasababu kuna, kwa mfano hili eneo nililokuambia, kwasababu huyu jirani wa hapa, yuko tayari kulitoa, walao lifanyiwe kazi mapema, walao hata tuwe na ile huduma.

IP kwa maana nyingine ni kwamba, tunapo tengeneza ripoti yetu, tuonyeshe kua kuna watu wako tayari kutoa maeneo (mmhhh), ili serikali itazame hilo.

RR sawasawa

I1 asante. basi nashukuru kwa mda wako na kwa utayari wako
