# Supplementary material for: It is beyond remuneration: Bottom-up health workers’ retention strategies at the primary health care system in Tanzania
Source: PLoS One. 2021 Apr 8;16(4):e0246262. doi: 10.1371/journal.pone.0246262 (PMC8031416; doi:10.1371/journal.pone.0246262)
Supplement: Attachment — Submitted filename: Response-PlosOne-R.doc [file pone.0246262.s002.doc]

**SCHOOL OF PUBLIC HEALTH AND SOCIAL SCIENCES**

***DEPARTMENT OF DEVELOPMENT STUDIES***

P.O. Box 65001

DAR ES SALAAM

TANZANIA

Web: http://www.muhas.ac.tz

Telephone: +255-22-2150302/6 Ext: 1230

Mob. no: +255-713-730179

Telefax: +255-22-2151238

E-mail: nsirili@yahoo.co.uk

**MMUHIMBILI UNIVERSITY OF HEALTH AND ALLIED SCIENCES**

14^th^ October 2020

Editor in Chief,

Plos One,

**RE: Resubmission of a manuscript titled*;* It is beyond remuneration: Bottom-up health workers’ retention strategies at the primary health care system in Tanzania**

Kindly refer to the caption above.

I am writing to resubmit a manuscript titled as above after addressing all issues raised by the reviewers for consideration to be published in your journal. This article is original and has not been published in any journal.

I consider your journal to be suitable for this manuscript as it directly focusses on the issues for improving health care delivery specifically clinical services.

The article is suitable to academicians, policymakers, planners and the general scientific community.

We hope that our article will be considered in your journal. I am attaching the detailed response matrix with this letter.

Yours sincerely,

Nathanael Sirili

Corresponding Author.

**Response to reviewers’ comments**

| **S/No** | **Comment** | Response | **Pg. no** |
| --- | --- | --- | --- |
| **Reviewer 1** | | | |
|  | This is an interesting article, and important contribution to the literature on the substantial human resources problems encountered in LMICs, and particularly in sub-Saharan Africa. | Thank you for the compliment |  |
|  | The authors include important information on “bottom up” strategies to improve health care worker (HCW) retention in rural areas, a problem with significant urgency in Tanzania, with such a large rural population and far too few health workers | Thank you very much |  |
|  | Because of this emphasis, the authors focus on ways to recruit and retain more HCWs identified by health system officers (DMOs, etc) and facility executive officers, who are “closer to the ground” on these issues and can offer important insights | Thank you very much |  |
|  | I think this summary and description of their suggestions is important, and am very supportive of this article being published, with the revisions I suggest below to strengthen this article: | Thank you very much |  |
|  | I think the biggest weakness of this article is the lack of much background, explanation, and description of the larger HR problems in the country, which I believe would strengthen this article and make it more widely relevant to the journal’s readership | Introduction section has been improved based on your very relevant comments. | Pg 2 to 5 |
|  | In a background section perhaps, a bit more information on the HR crisis in TZ, and I think more clearly and expansively explaining why it’s important to survey health workers on retention strategies in a “bottom up” way is important. Because the authors themselves have already contributed a significant amount of excellent scholarship on this topic, I believe this is possibly somewhat easily done. Several of my comments relate to this overarching weakness. | The background has been enriched by highlighting the development partners support and their challenges. | Pg 2 to 5 |
|  | The title is “beyond remuneration” but the authors don’t explain this – was this a quote? Does this reference that the TZ MoHSW doesn’t have funding to provide more adequate remuneration, meaning facilities/districts need to find money elsewhere to provide to HCWs? Do the HCWs themselves consider their current remuneration inadequate? Some more explanation about this, perhaps in a “background” section that more fully outlines the problems of HCW staffing and retention would help to strengthen the article. | Information on how the district incentives varies have been included in the background. The role of central government in provision of salaries to the health workers at the districts has been explained. | Pg 4 to Pg 5 |
|  | Are there any “perks” given by the central gov’t for working in rural areas (outlined briefly on. P. 3) found to be useful or working to retain HCWs? Or is much of the problem because the gov’t doesn't have the funding to support the strategies it tries to implement, as the authors sort of suggest (“often financial incentives have failed to materialize…”? Since the argument is that centralized, top-down strategies are inadequate, a bit more description of what those top-down strategies are would help clarify (related to the description in the intro, on p. 2). | More details have been added in the background on the role of central and local governments in the retention of health workers at the districts. Including the strategies that are proposed by the central governments and advocated to be implemented at the districts. | Pg 2 to 3 |
|  | More explanation about the historical process of decentralization and the 2006 creation of a partially centralized system (p. 2- 3) – particularly as all the data presented here sort of toggles back and forth between centralized and decentralized issues (like top-down and bottom-up approaches, which I’m roughly mapping on to that distinction). It would also help to clarify how funding works in a decentralized process – to what degree are regions, districts, and facilities responsible for funding salaries, accommodations, and other incentives the authors describe? In some places the authors write about income generating activities, does this mean they don't receive enough from the gov’t, or need to generate funding from local sources? For some context, see Marten & Sullivan (2020) – Hospital Side-Hustles: Funding Conundrums and Perverse Incentives in the Publicly-Funded Tanzanian Health Sector. Social Science & Medicine 244: 112662. | The funding process has been explained. The central government is responsible for salaries while the districts are responsible for other incentives. | Pg 4 |
|  | Regarding the selection of Kilwa and Rombo – why do the two zones have different health worker shortages? Do they have different issues? Did Rombo experience fewer issues of HCW shortage, and why? Does Kilwa have bigger problems, and why? I realize the authors have previously written about this, but a very short description of the background here would help situate the results. | This has been explained. The variation in incentives, social services availability and cultural practices partly contribute to the different magnitude of shortages. | Pg 6 |
|  | P. 3, “in the partial centralized system…” unclear the meaning of this sentence | The partial centralized system has been explained as a system that combines some elements of the centralized system and those of the decentralized system | Pg 3 |
|  | -P. 3, “Most of these strategies…and …this has created challenges like poor understanding…” more explanation of this would help readers better understand the need for more bottom-up strategies like the authors propose | Thank you very much, details have been added on the existing strategies and their challenges | Pg 3 to pg 4 |
|  | -P. 4, unsure what Kilimanjaro’s “better health workers’ availability” means | Thank you, the latter has been explained to mean health workers’ density | Pg 6 |
|  | -P. 5, “natural settings of the informants” sounds awkward, perhaps just something like “the interviews were carried out in informants’ offices or in designated rooms at their facilities” ? | Thank you, the word natural setting has been removed |  |
|  | -P. 8, the subhead “enhancing good working relationship…” – more explanation of why this might work | Thank you very much, details have been included that good working relationship made the health workers feel a sense of belongingness. | Pg 9 to Pg 15 |
|  | P. 8, “fostering good cooperation” – this seems like an outcome, rather than a strategy that could be implemented – did the informants describe how trust was fostered? A bit more explanation here | The word fostering has been replaced by promoting. This was indeed a reported outcome, however, we feel that from this outcome a strategy can be devised towards promoting cooperation | Pg 9 to Pg 15 |
|  | P. 9, in “district-level” subhead “provision of career opportunities: “inclined to the cadres” is unclear; clarify what support means, as it is frequently mentioned but is a somewhat vague term that could likely mean a lot of things – | Thank you very much, the word:   1. Career opportunity has been qualified to be an education career opportunity 2. Inclined has been removed and replaced to show that priority was given to cadres with a severe shortage 3. Support improved to mean financial support | Pg 9 to Pg 15 |
|  | P. 10, somewhat unclear about the process of limiting migration of HCWs – how or why do HCWs migrate? Why would district authorities have the power to migrate HCWs outside a district or region? A bit more detail here | Thank you very much, as detailed in the improved introduction, HCWs do migrate from place to another in Tanzania due to high variation in economic development and thus living condition. The migration from one district to another must be approved by the permanent secretary responsible for the local government after receiving recommendations from the districts and regions authorities. The regional and district authority can therefore justify as to why the migration should not be allowed. | Pg 2 to Pg 5 |
|  | Not sure Table 1 is necessary? Not sure it adds much to the paper. | Thank you very much, table 1 intends to provide an understanding of the geographical zones in Tanzania |  |
|  | Some explanation either in the subheadings under Results, or in the discussion, of whether these strategies were deemed effective (or if informants thought they would be effective) at retaining HCWs, and perhaps scalable? | Thank you very much a statement has been added in each strategy. The statement is detailing the feelings of the study participants on the applicability of the strategy in retention of health workers. | Pg 9 to Pg |
|  | p. 11 – related to some of the points outlined above, regarding more background explanation of the HR for health crisis in Tanzania, more description about what subsistence allowances are, HCW salaries, etc. I know that some changes in salaries, pensions, health insurance, etc. have been implemented recently in TZ to address HR issues, it would be helpful to know more about these changes, (in a background section) and if HCWs themselves believe their salaries to be adequate? | Thank you very much, the background section has been improved to detail the responsibilities of the central government and local government. Furthermore, the challenge of salary and other incentives has been explained. | 15Pg 2 to pg 5 |
|  | p. 15 – In the discussion, it appears as though some of the burdensome consequences of decentralization (asking local communities to fund services) and neoliberalism/austerity are being supported by the authors (which is related to why they didn’t work effectively)? I disagree somewhat with the assessment that local communities need “to lessen the burden that the gov’t must carry”; as a newly lower middle-income country, the TZ gov’t has more money than the rural poor to pay user fees, etc. Or is the argument that NGOs should support this more? Unclear which stakeholders are asked to shoulder this burden… | Thank you very much. Clarity has been added. Not all strategies require money. Some of the strategies like the good relationship between community and health workers require just a commitment of the local leaders. Therefore, the role of the stakeholders have been detailed. | Pg 15 to Pg 19 |
|  | p. 15 – “this strategy might work better in this era where there are many graduates…” – can the authors clarify this a bit – some more information about changes in the numbers of students graduating, hiring practices by the central gov’t, salary lines available, etc. to reduce the HR crisis? Why aren’t all grads employed if there is a HR crisis? Some reference to financial constraints here would be helpful, or maybe added to a background section | Thank you very much. Additional information on the increased graduates and limited employment due to financial constraint has been added at the background section. | Pg 15 to Pg 19 |
|  | p. 16 – “the provision of career opportunities” – provide an example to clarify what kind(s) of career opportunities are promoted | Thank you, it has been explained as an opportunity for further education and financial support | Pg 15 to Pg 19 |
|  | p. 16 – unclear the two sentences beginning with “however, a study by Sirili et al. 2018…” – not sure what staffing levels at specialized cadres means…some clarification | It has been explained that there is no defined arrangement for employing specialists at the district level. Therefore, allowing the existing cadres to go for further education to become like an organized exit plan for health workers. | Pg 18 |
|  | p. 16 – study trustworthiness should probably go in the methods section | This part has been renamed methodological consideration and study limitations added and discussed together. We feel that it should remain after the discussion as it discusses methodological considerations concerning the whole study to include the findings. | Pg 19 to pg 20 |
|  | There are some typos and some awkwardly worded sections, like at the very bottom of p. 15 ("although"), p. 2, "poor working condition" should be "conditions", p. 2 "Tanzania, an East African country as for many other countries" is awkward, p. 3, unsure what the "latter" is referencing, unclear, p. 10 "suffocation" should probably just be "the districts were seriously constrained", | Thank you very much, the document has copy-edited and typos and grammatical errors have been reduced. | Fully length document |
| **Reviewer 2** | | | |
|  | The topic for the study is relevant in current context where there is a problem of retention of health workforce specially in developing countries. I have few comments to offer: | Thank you |  |
|  | Methodology - Are the two jurisdictions selected randomly our of poor and well-off jurisdictions from North and South Zone. You may make a table showing different health workforce indicators of two jurisdictions. -Why only selected numbers of informant interviewed | Details have been added on site selections and why selected informants. | Pg 6 |
|  | Overall, the results are nicely presented | Thank you for the compliment |  |
|  |  |  |  |
|  | Discussion - The discussion can upfront mention the novel key findings in the beginning. -There is ample literature on retention of health workforce in developing nations, like India, South Africa etc. The author could have mentioned few of them - The limitations and strengths of the study could have been mentioned. | Thank you very much. Key findings have been summarized at the beginning of the discussion part.  References have been included.  Limitations have been included. | Pg 15 to Pg 19 |
|  |  |  |  |
| **Reviewer 3** | | | |
|  | Overall, the manuscript tackles an important area of public health significance looking at how to retain health workers in rural areas to provide the needed healthcare to people. However, the paper requires some major revisions to be acceptable for publication. | Thank you for the compliment |  |
|  | Abstract |  |  |
|  | 1.In the method section, it would be important for the authors to briefly state the type of study and also the sampling strategy used to select participants. | Thank you very much, study design and sampling strategy has been added. | Pg 1 |
|  | Main text |  |  |
|  | Introduction 1.It would be important to review literature and look at the effects of the problem. If it is difficult to retain health workers in rural areas, what are the effects in terms of health care delivery? | Thank you very much, the introduction section has been strengthened to include the effects of a shortage of health workers in rural areas. | Pg 2 to pg 5 |
|  | Study design 1.In the study design section, the authors need to give more explanation to justify why qualitative method was appropriate for this study. | The justification for why qualitative study design was appropriate has been included. | Pg 5 |
|  | The authors also need to state the category of people included in the study | Categories of people interviewed have been stated. | Pg 7 |
|  | Study site 1.It would be important that the authors provide information on the number of health facilities in the rural districts where the study was conducted and also the number of health workers in the two districts. | Thank you very much for this comment. As this was a qualitative study informed by the shortage of health workers based on workforce density of the zones and regions; and also, by the existence of proactive staff recruitment in the southern zone, we did not dwell much to get the individual numbers of the workers in the districts neither the total number of health workers in each district. However, we appreciate this comment for the future. |  |
|  | 2.Are they all located in rural areas or one is in rural area while the other is located in urban area? It is important to provide this information. | Both districts are rural. That was the focus of this study | Pg 6 |
|  | 3.Also what is the population in the study area? | This information can easily be extracted from the national census, however as we dwelt more into qualitative and retention strategies, we fell that adding the population in each district without quantitatively describing the health workforce availability will add confusion to this article. |  |
|  | Recruitment of participants and data collection 1.The statement that reads: “Therefore, District Medical Officers, District Health Secretaries, District Executive Directors, the District Hospital Medical Officers in charge, Health Centers medical in-charge, Ward Executive Officers, dispensaries Clinical Officer-in-charge and the Village Executive Officers” The sentence does not read well and needs some revision. | Thank you, this statement has been revised. | Pg 6 |
|  | 2.The authors only stated that purposive sampling was used in the study without describing processes or how this sampling strategy was used to select study participants. The authors need to tell the readers how the sampling strategy was used to select participants. | Sampling strategy has been explained including how the sites and facilities were selected. | Pg 6 |
|  | 3.Just stating that 3 FGDs were conducted is not enough. The authors need to describe how the FGDs were organized and also the KIIs. How many people constituted one FGD? Were males and female combined? | Details have been included, including table 2. Again, as the criteria was health facility governing committee members, the FGD was not disaggregated by sex. | Pg 7 |
|  | 4.In the data collection section, the authors need to state the number of KIIs and FGDs that were conducted before the table. |  |  |
|  | 5.The authors mentioned that the FGDs were conducted with 19 members of Health Facility Governing Committees. Who are these people and what role do they play at the health facility level and why was it important to include them in the study? | Thank you very much, the health facilities governing committees have been explained in the introduction part to include their roles. | Pg 3 |
|  | 6.The authors did not describe the experience and qualification of those who conducted the interviews? Were they trained and for how long before the interviews were conducted? Was pre-test done to test the interview guides? All these things need to be stated in the method section as well. | The experience of the research assistant has been added. The tool was not tested as this was an emergent design and thus the tool was enriched from the guiding questions as the study was progressing. | Pg 8 |
|  | Data analysis 1. What strategies did the authors use to arrive on the themes? This need to be stated clearly in the data analysis section. | Thank you very much. Qualitative content analysis as explained by Granheim and Lundman was used to arrive at the categories and subcategories. A reference has been added. | Pg 8 |
|  | 2.How many people were involved in the coding? | Two coders were involved with the first author as the main coder. This has been explained in the data analysis section. | Pg 8 |
|  | 3.Please include the ethics approval ID in the ethical consideration paragraph? | The ethical clearance reference number has been added. | Pg 8 |
|  | Results 1.I suggest all quotations should be in italic form. | Thank you for the suggestion. Point taken and adhered. | Pg 9 to 15 |
|  | 2.In each of the factors mentioned, it will be important to look at views expressed by study participants in the two study districts. Also, are all retention factors reported in the study mentioned in two districts or some were mentioned in one study district while others, also mentioned in the other district. It will be important to look at differences in views in the two districts concerning the retention factors reported. | The results section has been improved to show some unique factors in the two districts. However, most of the strategies were overlapping. | Pg 9 to 15 |
|  | 3.Limiting migration was reported as district level strategy to retain health workers in the study. Is the problem of retention of health workers limited to them moving from the rural area to the urban area or there are instances where some even leave the service and take up other opportunities elsewhere? If taking opportunities elsewhere is one factor, how will limiting migration solve the problem as reported in the study? | Thank you very much, leaving the sector is not a major problem at least in the health sector in Tanzania. That is why in this district, for instance, the management decision of limiting migration was sound feasible. |  |
|  | 4.Though, so many retention strategies were reported in the study, some of them might have been mentioned by only few participants meaning they are less important. I suggest the authors only pick the more important ones and report on them. Other strategies are only temporally e.g Active headhunting at the training institutions. | Thank you very much. These strategies may seem to be many, however, taking into account the nature and complexity of health workers retention, we have chosen to discuss these few strategies among many revealed. Again, the headhunting although seems to be a short-term strategy, its impact on retention was explained to be high. |  |
|  | 5.The authors mentioned that both FGDs and KIIs were conducted in the study. Reading through the quotation, I did not see quotations from the FGDs. If quotes from the FGDs were also used then it will be important to differentiate them by labeling the quotes appropriately using KIIs and FGDs throughout the results section. | Thank you very much, it was an oversight. A quote from the FGD has been included. | Pg 9 |
|  | 6.Was frequent visit by health authorities at the district office to health workers working in rural areas mentioned as one of the strategies to retain health workers in Tanzania? Check an article that was published | Thank you very much. This was mentioned as one of the top-bottom approaches to identify and solve challenges in rural areas. However, as our study focused on bottom-up approaches we felt that it was not appropriate to be included in this article. |  |
|  | Discussion 1.In the discussion section, so much emphasis was placed on reports from previous studies to the neglect of the implication of the study results. The authors need to take the key factors/findings of the study and discuss them thoroughly, point out the reasons for the findings and the implications of the findings of the study. What could happen if the situation is not addressed? All these need to come out in the discussion. | Thank you very much. In general, the discussion section has been strengthened. | Pg 15 to 19 |
|  | 2.There is need for proof reading of the manuscript | Proofreading of the manuscript has been done. | Fully length document |
