# Supplementary material for: It is beyond remuneration: Bottom-up health workers’ retention strategies at the primary health care system in Tanzania
Source: PLoS One. 2021 Apr 8;16(4):e0246262. doi: 10.1371/journal.pone.0246262 (PMC8031416; doi:10.1371/journal.pone.0246262)
Supplement: Attachment — Submitted filename: PONE PONE-D-20-09192R1.docx [file pone.0246262.s003.docx]

**PONE-D-20-09192R1**

**It is beyond remuneration: Bottom-up health workers’ retention strategies at the primary health care system in Tanzania**

**Further comments to the authors**

1. The authors need to explain in more details how the purposive sampling method was used to select study participants. Mere mention of purposive sampling is not enough. Please, state how participants were selected for the KIIs and the FGDs.
2. 3 GFDs were conducted with 19 participants.
a. How many people formed a group?
b. Were males and females brought together for the discussions?
3. In the analysis paragraph, there is a statement that reads “**The codebook was discussed with the second author and after the agreement, the codebook was then in NVIVO version 11 software before the coding”** is not clear. The authors need to revise this statement.
4. State the epistemological approach adopted for the work. Was it interpretative or phenomenological, inductive or deductive? The authors need to have a statement to clarify this.
5. Also clarify how socially desirable responses, confidentiality and privacy was ensured in this study.
a. How was the informed consent obtained for the FGDs? Was it a group or individual consent?
6. There is need for proof reading to correct grammatical errors.
