# Supplementary material for: It is beyond remuneration: Bottom-up health workers’ retention strategies at the primary health care system in Tanzania
Source: PLoS One. 2021 Apr 8;16(4):e0246262. doi: 10.1371/journal.pone.0246262 (PMC8031416; doi:10.1371/journal.pone.0246262)
Supplement: Attachment — Submitted filename: Response to Reviewers_.doc [file pone.0246262.s004.doc]

**SCHOOL OF PUBLIC HEALTH AND SOCIAL SCIENCES**

***DEPARTMENT OF DEVELOPMENT STUDIES***

P.O. Box 65001

DAR ES SALAAM

TANZANIA

Web: http://www.muhas.ac.tz

Telephone: +255-22-2150302/6 Ext: 1230

Mob. no: +255-713-730179

Telefax: +255-22-2151238

E-mail: nsirili@yahoo.co.uk

**MMUHIMBILI UNIVERSITY OF HEALTH AND ALLIED SCIENCES**

7^th^ January 2021

Editor in Chief,

Plos One,

**RE: Re-Resubmission of a manuscript titled*;* It is beyond remuneration: Bottom-up health workers’ retention strategies at the primary health care system in Tanzania**

Kindly refer to the caption above.

I am writing to resubmit a manuscript titled as above after addressing all issues raised by the reviewers for consideration to be published in your journal. This article is original and has not been published in any journal.

I consider your journal to be suitable for this manuscript as it directly focusses on the issues for improving health care delivery specifically clinical services.

The article is suitable to academicians, policymakers, planners and the general scientific community.

We hope that our article will be considered in your journal. I am attaching the detailed response matrix with this letter.

Yours sincerely,

Nathanael Sirili

Corresponding Author.

**Response to reviewers’ comments**

| **S/No** | **Comment** | **Response** | **Pg inserted** |
| --- | --- | --- | --- |
|  | **Editor’s comment** |  |  |
|  | Please check and correct the grammatical errors throughout the manuscript which is deemed necessary | Thank you very much. We have taken the article to a professional editor and now it reads better | Full lengthy article |
|  | **Reviewers’ comments** |  |  |
| 1 | The authors need to explain in more details how the purposive sampling method was used to select study participants. Mere mention of purposive sampling is not enough. Please, state how participants were selected for the KIIs and the FGDs. | Thank you for this wonderful comment. Details have been provided on how purposeful sampling was conducted for the KIIs and the FGDs | Pg 7 |
| 2 | 3 FGDs were conducted with 19 participants.   a. How many people formed a group? b. How were they obtained? With the assistance of VEO? Given names and invited them? c. Were males and females brought together for the discussions? | a. Each FGD was comprised of 6 to 7 members b. The FGD participants were obtained With the assistance of VEO and incharge of health facilities. They provided contacts and alerted them that they would be contacted by a researcher. c. Male and female participants were mixed because the HFGCs are formed based on role/position representation. See composition in the introduction section. Given the leadership position, members of the committee were self-assertive. This ensured full involvement of all participants in the discussions. Furthermore, the moderator ensured involvement by giving each participant opportunity to contribute. | Pg 8 |
| 3 | In the analysis paragraph, there is a statement that reads “**The codebook was discussed with the second author and after the agreement, the codebook was then in NVIVO version 11 software before the coding”** is not clear. The authors need to revise this statement. | **The statement has been revised; it now reads;**  The codebook was discussed with the second author and after agreement, the codebook was imported to NVIVO version 11 software for coding. |  |
| 4 | State the epistemological approach adopted for the work. Was it interpretative or phenomenological, inductive or deductive? The authors need to have a statement to clarify this. | The epistemological approach has been explained in the analysis subsection. “We adopted a hybrid approach in which both inductive and deductive analysis were conducted. A deductive approach was used to develop a preliminary codebook by the first author based on the objectives of the study and familiarity with the data. On the other hand, inductive approach was used to add new codes that emerged from the data during analysis.” | Pg 8 to pg 9 |
| 5 | Also clarify how socially desirable responses, confidentiality, and privacy were ensured in this study | Thank you very much, this has been explaind in the methodological considerations “… the fact that interviews were conducted by a medical doctor (the first author) might have influenced informants to respond in a socially desirable manner. However, this was offset by having research assistants with social sciences background.  With regards to confidentiality and privacy we have added the following information “… and they were encouraged to respond transparently as information from them will only be used for research purpose … their participation was purely voluntary and they can withdraw from the interview or discussion any time they wish to do so with no repurcussion.” “Confidentiality of the information was ensured by avoiding mentioning names or titles the key informants or FGD participants during recording. FGDs participants were assigned numbers that were kept separately from from the audio record descriptions. To ensure privacy typed notes and audio records were kept in a pined folder in a computer of the first author that was only accessible to him. Only the transcripts were shared with the second author.” | Pg 9 to pg 10 and pg 21 |
| 6 | How was the informed consent obtained for the FGDs? Was it a group or individual consent? | Thank you, we have explained that “Written informed consent was obtained individually from each study participant before the commencent of the interview or the discussion” | Pg 9 |
